# Synthesis of Veliparib Prodrugs and Determination of Drug-Release-Dependent PARP-1 Inhibition

**DOI:** 10.1021/acsmedchemlett.3c00065

**Published:** 2023-04-24

**Authors:** Matteo Borgini, Peter Wipf

**Affiliations:** Department of Chemistry, University of Pittsburgh, Pittsburgh, Pennsylvania 15260, USA

**Keywords:** PARP inhibitors, racemic veliparib, prodrugs, mitochondria-targeting, XJB-5-131

## Abstract

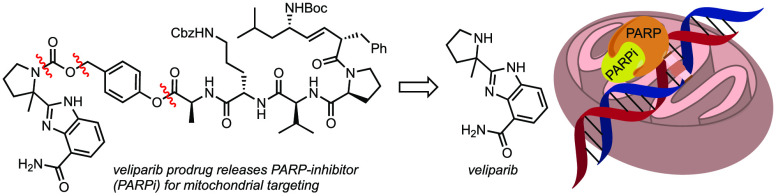

Poly(ADP-ribose) polymerase (PARP) plays a key role in repairing DNA damage, and several PARP inhibitors have been approved as treatments in BRCA1/2 mutated breast and ovarian cancers. Mounting evidence also supports their application as neuroprotective agents since PARP overactivation compromises the mitochondrial homeostasis by consumption of NAD^+^ reserves, leading to an increase in reactive oxygen and nitrogen species and a spike in intracellular Ca^2+^ levels. Herein, we present the synthesis and preliminary evaluation of new mitochondria-targeting PARP inhibitor prodrugs of (±)-veliparib, with the goal to advance potential neuroprotective properties without impairing the repair of damaged DNA in the nucleus.

Neuronal cell death manifests itself in cognitive dysfunction, motor impairment, and behavioral alteration, giving rise to neurodegenerative diseases and dementia.^1,2^ Alzheimer’s disease (AD), Parkinson’s disease (PD), Huntington’s disease (HD), amyotrophic lateral sclerosis (ALS), and traumatic brain injury (TBI) already affect millions of people worldwide, and dementia threatens to become the most common human disease at the time of death.^3,4^

Mitochondrial dysfunction is a shared hallmark of aging and neurodegeneration caused by persistent oxidative stress and increased mitochondrial Ca^2+^ uptake, leading to the impairment of the electron transport chain and a decrease in ATP production.^5,6^ Considerable efforts have been made to identify the key molecular determinants for these pathogenic events, and several candidates have been identified.^7−9^ We have previously shown that mitochondria-targeting electron and radical scavengers, e.g., nitroxides XJB-5-131 and JP4-039, are effective *in vivo* to mediate injury-induced neuronal death^10^ and delay the onset of neurodegenerative diseases such as HD, TBI, and progeria.^11−13^ These results inspired us to investigate the effect of mitochondria-targeting PARP-1 inhibitors.^14^ The poly(ADP-ribose) polymerase (PARP) family of enzymes plays an important role in DNA repair, genome maintenance, chromatin remodeling, transcription regulation, stress response, and regulation of cell death.^15,16^ PARPs catalyze the PARylation of target proteins by tagging them with polymers of ADP-ribose (PAR), using NAD^+^ as substrate, and releasing nicotinamide as a side-product. PARP-1, which is responsible for about 90% of the PARP functions,^17^ is primarily involved in the repair of DNA single-strand breaks (SSBs). Upon recognition of SSBs, PARP-1 PARylates itself, which triggers the recruitment of other proteins involved in the DNA repair machinery and, upon their PARylation, activates DNA repair.^16^ Most PARP inhibitors interfere with the catalytic cycle, preventing PARP-1 release from DNA, sidetracking DNA repair, and amplifying DNA damage.^18^

Several dual PARP-1/2 inhibitors (e.g., olaparib, niraparib, rucaparib, and talazoparib) have been approved by the U.S. Food and Drug Administration (FDA) and the European Medicines Agency (EMA) for the treatment of BRCA-deficient breast, ovarian, fallopian tube, and primary peritoneal cancers (Figure 1).^19^ Pamiparib and fuzuloparib were recently approved in China by the National Medical Products Administration (NMPA) for the treatment of germline BRCA mutation-associated recurrent advanced ovarian, fallopian tube, and primary peritoneal cancers.^20,21^ Veliparib (ABT-888) is another PARP-1/2 inhibitor under clinical investigation for the treatment of BRCA breast and ovarian cancers.^22,23^

**Figure 1:**
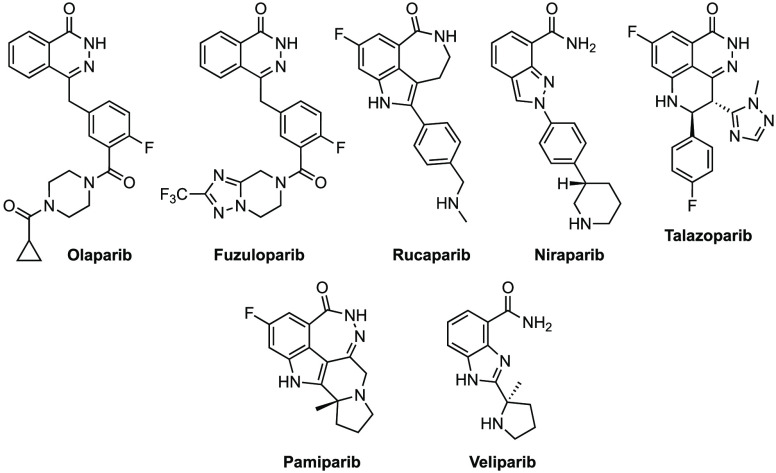
Representative dual PARP-1/2 inhibitors on the market or under late-stage clinical development.

Veliparib does not lock PARP-1 enzyme onto DNA as potently as other inhibitors,^24^ and its more favorable hematotoxicity profile makes it an attractive alternative for combination chemotherapies. This inhibitor received an FDA orphan drug designation for the treatment of epithelial ovarian cancer in combination with DNA-damaging agents as well as for advanced squamous non-small-cell lung cancer.^25^

Overactivation of PARP-1 causes the depletion of the NAD^+^ pool, thus inhibiting ATP production in mitochondria and leading to energy failure and cell death. In addition to NAD^+^/ATP depletion and bioenergetic collapse, PARP-1 overactivation increases intracellular Ca^2+^ levels by augmenting the influx of extracellular Ca^2+^ as well as the release of Ca^2+^ from the endoplasmic reticulum, leading to an uncontrolled increase of mitochondria-damaging reactive oxygen and nitrogen species (ROS/RNS).^26^ Due to the close link between PARP-1 activity and cellular energy failure, PARP-1 inhibition is therefore emerging as a potential treatment of neuroinflammation and neurological disorders.^27^ Several preclinical experiments with PARP-1/2 inhibitors showed beneficial effects for non-oncological conditions such as TBI,^28^ AD,^29^ HD,^30,31^ neuropathic pain,^32^ ischemic stroke,^33^ retinitis pigmentosa,^34^ depression,^35^ and chronic asthma,^36^ as well as in early stages of acute ischemic stroke^37^ and pulmonary arterial hypertension.^38^ As a follow-up of our preliminary analysis of a mitochondria-targeting veliparib analog, **1**,^14^ we now report the synthesis of a second generation of targeted analogs, **2**–**7**, exploring different linkers between the mitochondria-targeting unit and veliparib, and exploiting a prodrug approach (Figure 2). We also report the results of an assay to monitor the *in vitro* PARP-1 inhibition for **2**–**7**.

**Figure 2:**
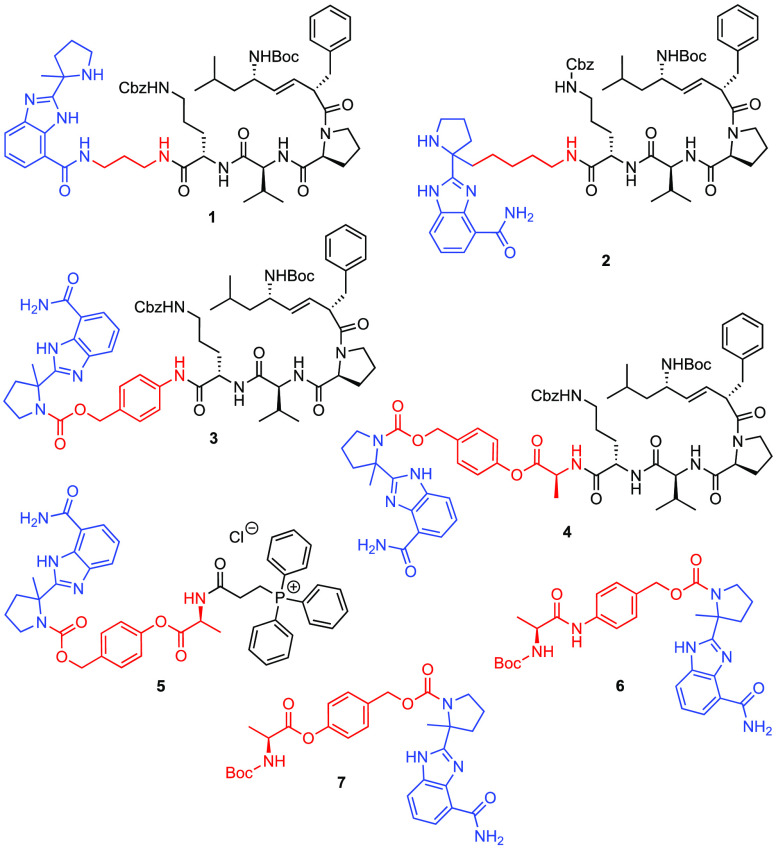
Mitochondria-targeting veliparib analogs and related prodrug derivatives. Color scheme: blue = veliparib or veliparib analog; red = linker or prodrug moiety; black = mitochondria-targeting moiety.

The X-ray structure of veliparib bound to PARP-1, PDB 2RD6,^39^ shows that the methyl group attached to the quaternary carbon in veliparib’s structure does not interact with the enzyme, and, in fact, (*R*)- and (*S*)-enantiomers of veliparib are equipotent.^22^ In light of this, we designed analog **2** linking the mitochondria-targeting moiety covalently at this position (Figure 2). This attachment maintains the quaternary center and avoids steric hindrance at the benzamide moiety, which is the key pharmacophore for PARP-1 interaction. The flexible 4-carbon linker to the mitochondria-targeting unit was introduced to allow for a reduction in the steric hindrance in the PARP-1 interaction of compound **2**. Alternatively, we designed pyrrolidine nitrogen-linked prodrugs **3** and **4**, which are capable of delivering structurally unmodified veliparib to mitochondria upon proteolytic cleavage of the prodrug by the unique set of mitochondrial proteases, i.e., mitoproteases.^40−43^ Prodrugs **3** and **4** are characterized by the use of self-immolative linkers *p*-aminobenzyl alcohol (PABA) and *p*-hydroxybenzyl alcohol (PHBA), respectively, to induce different metabolic susceptibilities (Figure 2). Upon cleavage of the *N*-phenyl amide or *O*-phenyl ester group in **3** or **4**, veliparib is released by a spontaneous vinylogous elimination cascade (Figure 3).^44−46^ In addition to the mitochondria-targeting sequence derived from XJB-5-131, the triphenylphosphonium (TPP) moiety has also been linked to veliparib through the PHBA group, generating analog **5**, to evaluate the effect of different mitochondria-targeting moieties on veliparib release and PARP-1 inhibition.^47^ Along with these targeted compounds, we prepared analogs **6** and **7** as non-specifically targeted veliparib derivatives and reference compounds.

**Figure 3:**
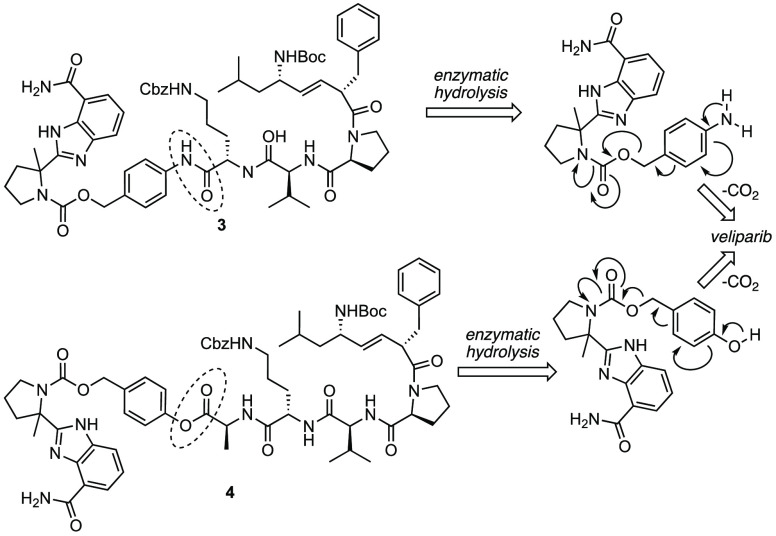
Mechanism of veliparib release from prodrugs **3** and **4** by enzymatic cleavage followed by a vinylogous elimination cascade.

## Analog Syntheses

**Scheme 1:**
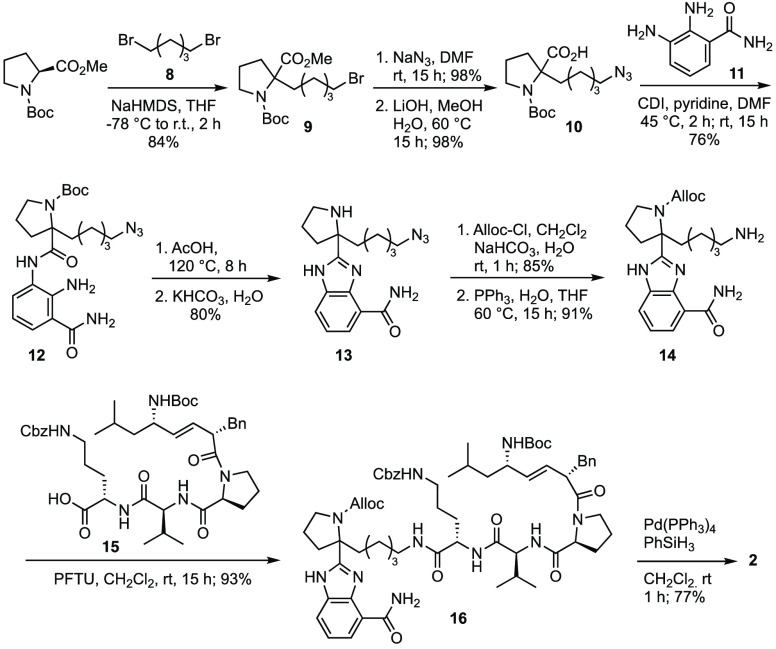
Synthesis of Covalently Modified Veliparib Analog **2**

After considerable experimentation, we identified and optimized a preparation of the covalently modified veliparib analog **2** that used an α-alkylation of *N*-Boc-l-proline methyl ester with 1,5-dibromopentane (**8**) to give bromide **9** in 84% yield (Scheme 1). Bromide displacement with sodium azide and subsequent saponification occurred in quantitative yield, giving intermediate **10**. Interestingly, the α-alkylation of *N*-Boc-l-proline methyl ester as well as *N*-Boc-l-proline with mesyl- or iodo-substituted azidoalkanes failed to give the corresponding alkylation products. Condensation with 2,3-diaminobenzamide (**11**) gave diamide **12** in good yield using 1,1′-carbonyldiimidazole (CDI) in a 1:1 mixture of DMF and pyridine. The benzimidazole was formed concomitantly with Boc-deprotection by dehydration of **12** in glacial acetic acid at reflux, followed by neutralization with saturated aq. KHCO_3_ to give secondary amine **13** in 80% yield. Subsequently, protection of the pyrrolidine with the allyloxycarbonyl (Alloc) group and reduction of the azide led to primary amine **14**. Coupling with the mitochondria-targeting unit, acid **15**,^48^ in the presence of pentafluorophenol tetramethyluronium hexafluorophosphate (PFTU) and Alloc removal provided **2** in high yield.

**Scheme 2:**
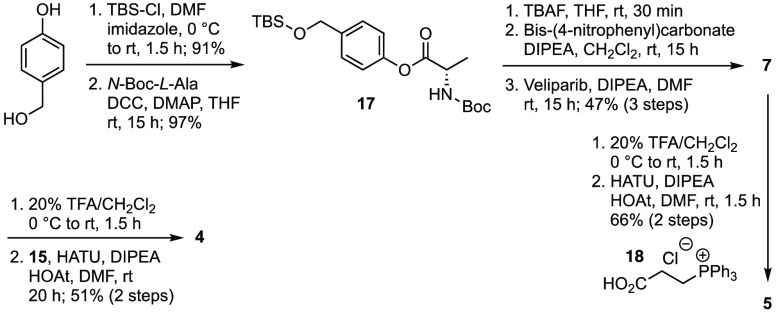
Synthesis of Veliparib Prodrugs **4** and **5** and Reference Compound **7**

For the preparation of prodrugs **4** and **5**, *p*-hydroxybenzyl alcohol was converted to the silyl ether with TBS-Cl in 91% yield and coupled to *N*-Boc-l-alanine with DCC to give ester **17** in 97% yield (Scheme 2). Removal of the silyl group with TBAF followed by carbonylation of the benzyl alcohol with bis(4-nitrophenyl)carbonate produced an unstable intermediate that was directly coupled to (±)-veliparib after removing the excess of bis(4-nitrophenyl)carbonate. The reference compound **7** was thus obtained in 47% yield over 3 steps and used for the further conversion to the desired prodrugs **4** and **5**. Boc-deprotection in 20% TFA in CH_2_Cl_2_, followed by a HATU-promoted coupling of the resulting TFA salt with acid **15**, led to **4** in 51% yield over 2 steps. Similarly, **5** was obtained in 66% yield after deprotection of **7** and coupling with (2-carboxyethyl)triphenylphosphonium chloride.

**Scheme 3:**
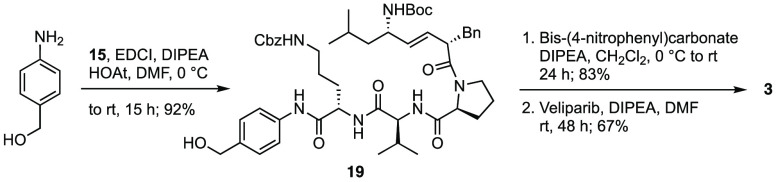
Synthesis of Veliparib Prodrug **3**

Veliparib prodrug **3** does not contain the additional alanine linker that was used for the attachment of the prodrug unit in **4** and **5**. The preparation of this compound from *p*-aminobenzyl alcohol and acid **15** with EDCI proceeded in 92% yield via amide **19** (Scheme 3). Treatment of the benzyl alcohol with bis(4-nitrophenyl)carbonate and DIPEA followed by condensation with veliparib provided carbamate **3** in 56% yield over 2 steps.

**Scheme 4:**
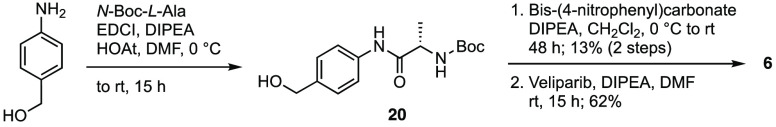
Synthesis of Reference Prodrug **6**

In a similar fashion, the non-targeted reference prodrug **6** was obtained from *N*-Boc-l-alanine, *p*-aminobenzyl alcohol, and veliparib in three steps and 8% overall yield (Scheme 4). Compound **6** represents a control that illustrates the significance of the more stable amide (in **6**) vs ester (in **7**) functional group linkage in the phenyl portion of the prodrug moiety.

## PARP-1 Inhibition Assays

**Figure 4:**
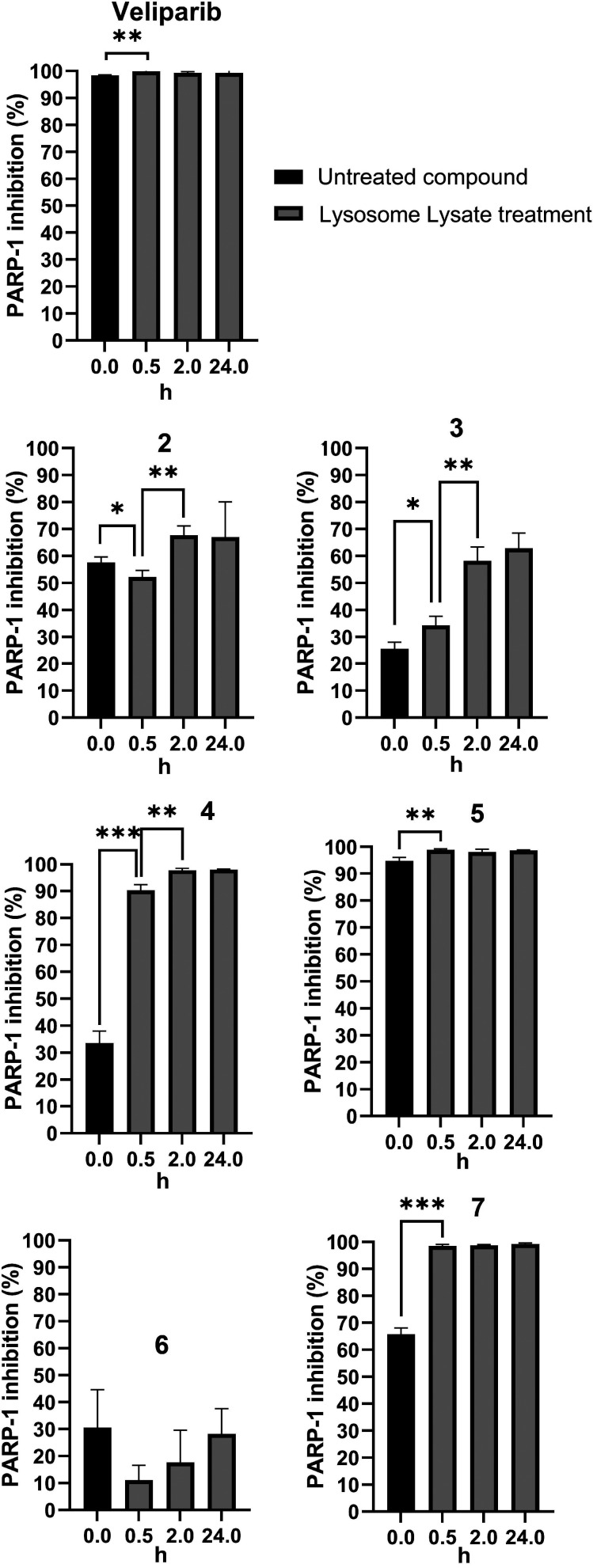
Graphical display of PARP-1 inhibitory activities of covalently linked derivative **2** and prodrugs **3**–**7** as well as veliparib before and after treatment with HLLLs after 30 min, 2 h, and 24 h. All analogs were tested at 0.5 μM concentration in the assay buffer. Veliparib was used as a positive control and displayed complete inhibition at 0.5 μM concentration. Statistically significant differences based on *P* value are indicated as follows: ns, *P* > 0.05; *P ≤ 0.05; **P ≤ 0.01; ***P ≤ 0.001.

In order to investigate the rate of release of veliparib and its correlation with the chemical nature of the prodrugs, we determined the PARP-1 inhibitory profiles of covalently linked derivative **2** and prodrugs **3**–**7** after 30-min, 2-h, and 24-h treatment with human liver lysosome lysate (HLLL) (Figure 4). Liver lysosomes contain several hydrolytic enzymes and were chosen as the metabolic matrix due to their compatibility with the assay. This assay protocol prevents any possible interference from lysosome residues for the evaluation of the PARP-1 inhibition (see SI). Veliparib (PARP-1 *K*_i_ = 5.2 nM; IC_50_ = 3.3 nM) was as used as a positive control and completely inhibited PARP-1 activity at 0.5 μM concentration.^49^ In contrast, prior to exposure to HLLL, compounds **2**–**7** showed only partial PARP-1 inhibition (30–60% inhibition at 0.5 μM), with the exception of TPP salt **5**, which was a strong inhibitor at all time points tested (95–99% inhibition). While this needs to be further investigated, the consistently high inhibition of PARP-1 suggests that the ester moiety in prodrug **5** is hydrolyzed at a similar rate or even faster compared to ester prodrugs **4** and **7** (Table 1). The linker and the nature of the moiety attached to veliparib play key roles in the PARP-1 inhibitory activity after HLLL exposure. In compounds **3**, **4**, and **6**, the high steric hindrance and/or lower linker flexibility are likely to prevent the benzimidazole amide moiety of veliparib from interacting closely with the enzyme, greatly reducing the PARP-1 inhibition of these analogs prior to cleavage of the linker moiety. The covalently linked analog **2**, in contrast, retained moderate inhibition (50–70%) at all time points, suggesting that linker cleavage was slow or absent under the assay conditions.

**Table 1:** Values and Standard Deviations for PARP-1 Inhibitory Activities of Covalently Linked Derivative **2** and Prodrugs **3**–**7** as well as Veliparib as a Function of HLLL Treatment at 0 min, 30 min, 2 h, and 24 h^a^

	PARP-1 inhibition (%)			
	before HLLL treatment	@ 30 min	@ 2 h	@ 24 h
veliparib	98.4 ± 0.2	99.9 ± 0.3	99.4 ± 0.4	99.3 ± 1.3
**2**	57.6 ± 2.1	52.3 ± 2.4	67.7 ± 3.5	67.0 ± 13.1
**3**	25.5 ± 2.6	34.3 ± 3.4	58.3 ± 5.1	62.9 ± 5.6
**4**	33.6 ± 4.4	90.3 ± 2.1	97.8 ± 0.7	98.0 ± 0.3
**5**	94.8 ± 1.3	98.9 ± 0.4	98.1 ± 1.0	98.7 ± 0.2
**6**	30.6 ± 14.0	11.1 ± 5.5	17.7 ± 11.9	28.3 ± 9.3
**7**	65.8 ± 2.3	98.5 ± 0.6	98.7 ± 0.3	99.2 ± 0.4

a All analogs were tested at 0.5 μM concentration in the assay buffer. Veliparib was used as a positive standard and displayed complete inhibition at 0.5 μM.

Interestingly, prodrugs bearing the cleavable ester linker, i.e., **4** and **7**, were quickly cleaved by the hydrolytic enzymes, resulting in a PARP-1 activity comparable to that of veliparib after 30 min of lysosome lysate treatment (for compound **4** from 33.6 ± 4.4% to 90.3 ± 2.1%; for compound **7** from 65.8 ± 2.3% to 98.5 ± 0.6%). Therefore, these analogs can be classified as fast-release prodrugs. As expected, however, the PARP-1 inhibition for the prodrug **3** connected with an amide group to the PABA linker, which renders it more resistant to enzymatic hydrolysis, showed a more gradual increase in PARP-1 inhibition even after several hours of HLLL treatment (from 25.5 ± 2.6% at 0 h to 58.3 ± 5.1% inhibition at 2 h). Compound **3** can therefore be classified as a slow-release prodrug. The amide-linked analog **6** showed the same trend as **3** but proved to be a substantially weaker inhibitor than the latter at the 30-min to 24-h time points.

## Conclusions

Although PARP is a well-established target for cancer treatment, evidence from *in vivo* models demonstrates a potential additional utility of PARP inhibitors as neuroprotective agents. The overactivation of PARP-1 caused by oxidative stress and genomic damage leads to NAD^+^ consumption and to an increase of intracellular Ca^2+^ concentration, impairing ATP production in mitochondria, and consequently leading to energy failure and cell death. Moreover, nuclear PARP-1 and mitochondrial (mtPARP-1) display opposite roles in maintaining the integrity of DNA and mitochondrial DNA (mtDNA), respectively. Hence, nuclear PARP-1 fosters the activation and recruitment of proteins involved in the DNA repair machinery, whereas mtPARP-1 has a pronounced inhibitory effect on mtDNA damage repair, negatively affecting mitochondrial biogenesis and mitochondrial function.^50^ Our previous work with mitochondria-targeting veliparib, **1**, demonstrated that the selective inhibition of mtPARP-1 can exhibit neuroprotective effects against various oxidative insults, allowing the repair of damaged mtDNA as well as nuclear DNA.^14^ In order to further investigate and optimize these effects, we prepared the second-generation PARP-1 inhibitor candidates **2**–**7**, characterized by a variation of the linker and prodrug units connecting veliparib and a mitochondria-targeting peptide isostere moiety. The prodrugs **3**–**7** were designed to release structurally unmodified veliparib upon cleavage by proteases. As a proof-of-concept study, we assayed the kinetics of PARP-1 inhibition and identified interesting trends in the rate and efficiency of drug release as functions of the linker structure and point of attachment on veliparib. The HLLL assay allowed us to identify prodrugs with slow- or fast-release profiles, as well as analogs that maintain their PARP-1 inhibitory activities upon exposure to hydrolytic enzymes. These results will be used in future studies to test the mitochondria-targeting PARP-1 inhibitors with the optimal PARP-1 inhibition kinetics in models of neurodegenerative diseases.

## Supporting Information Available

Experimental details, ^1^H and ^13^C NMR spectra for new synthetic products, and assay information (PDF)

## Abbreviations

ATP: adenosine triphosphate
Alloc: *N*-allyloxycarbonyl
DCC: *N,N*′-dicyclohexylcarbodiimide
DIPEA: *N,N*-diisopropylethylamine
DMF: dimethylformamide
DMSO: dimethyl sulfoxide
EDCI: 1-ethyl-3-(3-(dimethylamino)propyl)carbodiimide
HATU: 1-[bis(dimethylamino)methylene]-1*H*-1,2,3-triazolo[4,5-*b*]pyridinium 3-oxide hexafluorophosphate
HLLL: human liver lysosome lysate
HOAt: 1-hydroxy-7-azabenzotriazole
HOBt: hydroxybenzotriazole
*K*_i_: equilibrium constant of the inhibitor combining with the enzyme–substrate complex
NAD^+^: nicotinamide adenine dinucleotide
NaHMDS: sodium bis(trimethylsilyl)amide
ROS: reactive oxygen species
SAR: structure–activity relationship
T3P: propanephosphonic acid anhydride
TBAF: tetra-*n*-butylammonium fluoride
TBS-Cl: *tert*-butyldimethylsilyl chloride
TFA: trifluoroacetic acid
