# Supplementary material for: Synthesis of Veliparib Prodrugs and Determination of Drug-Release-Dependent PARP-1 Inhibition
Source: ACS Med Chem Lett. 2023 Apr 24;14(5):652–7. doi: 10.1021/acsmedchemlett.3c00065 (PMC10184315; doi:10.1021/acsmedchemlett.3c00065)
Supplement: Supplementary file 1 — ml3c00065_si_001.pdf [file ml3c00065_si_001.pdf]

Synthesis of Veliparib Prodrugs and Determination of Drug Release-Dependent PARP-1 Inhibition

Matteo Borgini and Peter Wipf*

Department of Chemistry, University of Pittsburgh, Pittsburgh, PA 15260, USA

pwipf@pitt.edu

Table of Contents

General Methods 2
Procedures 3
1-(*tert*-Butyl) 2-methyl 2-(5-bromopentyl)pyrrolidine-1,2-dicarboxylate (**9**) 3
2-(5-Azidopentyl)-1-(*tert*-butoxycarbonyl)pyrrolidine-2-carboxylic acid (**10**) 3
2,3-Diaminobenzamide dihydrochloride (**11**) 4
tert-Butyl 2-((2-amino-3-carbamoylphenyl)carbamoyl)-2-(5-azidopentyl)pyrrolidine-1-carboxylate (**12**) 4
2-(2-(5-Azidopentyl)pyrrolidin-2-yl)-1*H*-benzo[*d*]imidazole-4-carboxamide (**13**) 5
Allyl 2-(5-aminopentyl)-2-(4-carbamoyl-1*H*-benzo[*d*]imidazol-2-yl)pyrrolidine-1-carboxylate (**14**) 5
Allyl 2-(5-((*S*)-2-((*S*)-2-((*S*)-1-((2*S*,5*S*,*E*)-2-benzyl-5-((*tert*-butoxycarbonyl)amino)-7-methyloct-3-enoyl)pyrrolidine-2-carboxamido)-3-methylbutanamido)-5-(((benzyloxy)carbonyl)amino)pentanamido)pentyl)-2-(4-carbamoyl-1*H*-benzo[*d*]imidazol-2-yl)pyrrolidine-1-carboxylate (**16**) 6
Benzyl ((4*S*)-4-((*S*)-2-((*S*)-1-((2*S*,5*S*,*E*)-2-benzyl-5-((*tert*-butoxycarbonyl)amino)-7-methyloct-3-enoyl)pyrrolidine-2-carboxamido)-3-methylbutanamido)-5-((5-(2-(4-carbamoyl-1*H*-benzo[*d*]imidazol-2-yl)pyrrolidin-2-yl)pentyl)amino)-5-oxopentyl)carbamate (**2**) 7
4-(((*tert*-Butyldimethylsilyl)oxy)methyl)phenyl (*tert*-butoxycarbonyl)-*L*-alaninate (**17**) 8
4-(((*tert*-Butoxycarbonyl)-*L*-alanyl)oxy)benzyl 2-(7-carbamoyl-1*H*-benzo[*d*]imidazol-2-yl)-2-methylpyrrolidine-1-carboxylate (**7**) 8
4-((((*S*)-2-((*S*)-2-((*S*)-1-((2*S*,5*S*,*E*)-2-Benzyl-5-((*tert*-butoxycarbonyl)amino)-7-methyloct-3-enoyl)pyrrolidine-2-carboxamido)-3-methylbutanamido)-5-(((benzyloxy)carbonyl)amino)pentanoyl)-*L*-alanyl)oxy)benzyl 2-(4-carbamoyl-1*H*-benzo[*d*]imidazol-2-yl)-2-methylpyrrolidine-1-carboxylate (**4**)... 9
(3-(((2*S*)-1-(4-(((2-(7-Carbamoyl-1*H*-benzo[*d*]imidazol-2-yl)-2-methylpyrrolidine-1-carbonyl)oxy)methyl)phenoxy)-1-oxopropan-2-yl)amino)-3-oxopropyl)triphenylphosphonium chloride (**5**) 10
(*S*)-2-((*S*)-2-((*S*)-1-((2*S*,5*S*,*E*)-2-Benzyl-5-((*tert*-butoxycarbonyl)amino)-7-methyloct-3-enoyl)pyrrolidine-2-carboxamido)-3-methylbutanamido)-5-(((benzyloxy)carbonyl)amino)pentanoic acid (**19**)........ 11
4-((*S*)-2-((*S*)-2-((*S*)-1-((2*S*,5*S*,*E*)-2-Benzyl-5-((*tert*-butoxycarbonyl)amino)-7-methyloct-3-enoyl)pyrrolidine-2-carboxamido)-3-methylbutanamido)-5-(((benzyloxy)carbonyl)amino)pentanamido)benzyl 2-(7-carbamoyl-1*H*-benzo[*d*]imidazol-2-yl)-2-methylpyrrolidine-1-carboxylate (**3**) 12
4-((*S*)-2-((*tert*-Butoxycarbonyl)amino)propanamido)benzyl 2-(4-carbamoyl-1*H*-benzo[*d*]imidazol-2-yl)-2-methylpyrrolidine-1-carboxylate (**6**) 13
Assays 15
PARP-1 Inhibitory Activity 15
Metabolic Studies and Related PARP-1 Inhibitory Activity........ 15
Copies of NMR Spectra 17

General Methods. All the reaction solvents were dried prior to use. THF was freshly distilled from a sodium/benzophenone radical anion solution, CH_2Cl_2 and toluene were freshly distilled (CaH_2), and acetonitrile was stored for at least 24 h over activated 3 Å molecular sieves. Et_3N, DIPEA, and pyridine were distilled (CaH_2. When necessary, degassed solvents were prepared by freeze-pump-thaw techniques. Unless stated otherwise, all reactions were performed under an atmosphere of N_2 that was passed through a short cartridge (10 × 2 cm) of drierite®. All glassware and stir bars were flame-dried prior to use. Reactions were monitored by TLC analysis (pre-coated silica gel 60 F254) and spots were visualized (UV lamp 254 nm and 395 nm). IR spectra were obtained using a PerkinElmer 100 IR-ATR spectrometer. Melting points were obtained using a Mel-Temp instrument and are uncorrected. $^{1}H/^{13}C$ NMR spectra were recorded on Bruker Avance 300/76 MHz, Bruker Avance 400/100 MHz, Bruker Avance 500/126 MHz, Bruker Avance 601/151 MHz, or Bruker Avance 701 MHz instruments. High resolution mass spectra were obtained on a Micromass UK Limited, Q-TOF Ultima API or a Thermo Scientific Exactive Orbitrap LC-MS. Chemical shifts were reported in parts per million (ppm) with the residual solvent peak (CDCl3: 7.26 ppm for ^{1}H, 77.16 ppm for ^{13}C; DMSO-d6: 2.50 ppm for ^{1}H, 39.52 ppm for ^{13}C) used as the internal standard. Chemical shifts were tabulated as follows: chemical shift, multiplicity (s = singlet, d = doublet, t = triplet, q = quartet, p = pentet, h = sextet, dd = doublet of doublet, dt = doublet of triplet, ddd = doublet of doublet of doublet, tt = triplet of triplet, dtd = double of triplet of doublet, dddd = doublet of doublet of doublet of doublet, dddt = doublet of doublet of doublet of triplet, dtdd = doublet of triplet of doublet of doublet, ddtd = doublet of doublet of triplet of doublet, dtt = doublet of doublet of triplet, m = multiplet, br s = broad singlet), coupling constant(s), and integration.

The purity of final products was assessed using an Agilent Technologies 1260 Infinity II LC at 220 nm UV absorption (Waters XBridge BEH C_{18} 2.1 × 50 mm, 2.5 µm) or an Agilent Technologies 385-ELSD (Microsolv Cogent 2.0 Bidentate C_{18} 2.1 × 50 mm, 2.2 µm; ELSD conditions: evaporator and nebulizer set at 45 °C; gas flow set at 1.80 standard liter/min). All final assay samples showed a purity >95% by LCMS analysis with UV (220 nm) and ELS detection.

No unexpected or unusually high safety hazards were encountered.

Procedures

1-(*tert*-Butyl) 2-methyl 2-(5-bromopentyl)pyrrolidine-1,2-dicarboxylate (**9**). To a flame-dried 250-mL round bottom flask charged with a solution of N-Boc-*L*-proline methyl ester (10.3 g, 44.9 mmol, 1 eq) in THF (60 mL, 0.7 M), a solution of NaHMDS (9.53 g, 49.4 mmol, 1.1 eq) in THF (20 mL) was added dropwise at -78 °C. The reaction mixture was stirred for 30 min at -78°C under nitrogen. Then, a chilled (-78 °C) solution of 1,5-dibromopentane **8** (15.3 mL, 112 mmol, 2.5 eq) in THF (10 mL) was added dropwise via cannula. The reaction mixture was stirred for 30 min at -78 °C, warmed to ambient temperature (90 min, stirred for an additional 2 h, quenched by the addition of saturated aq. NH_4Cl and extracted with ethyl acetate (2x). The combined organic extracts were washed with brine, dried ($MgSO_4$), filtered and concentrated in vacuo. The crude residue was purified by chromatography on SiO_2 (25% ethyl acetate in hexanes) to give **9** (14.2 g, 37.6 mmol, 84%) as a colorless oil. **IR** (ATR, CH_2Cl_2) 2974.1, 1739.2, 1693.8, 1389.0, 1161.2 cm^{-1}; **^{1}H NMR** (300 MHz, Chloroform-*d*) δ 3.62–3.51 (m, 3.5 H), 3.47 (ddd, J = 10.4, 7.5, 5.7 Hz, 0.5 H), 3.27 (td, J = 6.9, 3.7 Hz, 3 H), 2.21–1.85 (m, 3 H), 1.83–1.59 (m, 5 H), 1.40–1.29 (m, 5 H), 1.27 (s, 6 H), 1.25–1.05 (m, 2 H); **^{13}C NMR** (76 MHz, Chloroform-*d*) δ 175.2, 175.0, 153.8, 153.5, 79.7, 79.2, 67.7, 67.2, 51.9, 48.4, 48.3, 37.3, 36.0, 34.8, 33.6, 33.5, 32.4, 32.3, 28.2, 28.2, 28.1, 28.0, 23.0, 22.6, 22.5, 22.4; ; **HRMS** (ESI^+) *m/z* calcd for $C_{16}H_{29}BrNO_4$ $[M+H]^+$, 378.1275; found, 378.1272.

2-(5-Azidopentyl)-1-(*tert*-butoxycarbonyl)pyrrolidine-2-carboxylic acid (**10**). To a flame-dried 50 mL round bottom flask charged with a solution of **9** (9.05 g, 23.9 mmol, 1 eq) in dimethylformamide (35 mL, 0.7 M) was added NaN_3 (4.66 g, 71.8 mmol, 3 eq). The reaction mixture was stirred at 25°C overnight under nitrogen. The reaction was quenched with water and extracted with ethyl acetate (3x). The combined organic layers were washed with water (3x), brine (3x), dried ($MgSO_4$, filtered, and concentrated to give 1-(*tert*-butyl) 2-methyl 2-(5-azidopentyl)pyrrolidine-1,2-dicarboxylate (7.97 g, 23.4 mmol, 98%) as a pale-yellow, oily mixture of rotamers that was used directly in the next step: **IR** (ATR, CH_2Cl_2) 2935, 2093, 1740, 1693, 1388, 1365, 1236, 1160, 1136 cm^{-1}; **^{1}H NMR** (300 MHz, Chloroform-*d*) δ 3.75–3.66 (m, 3.5 H), 3.60 (ddd, J = 10.5, 7.3, 5.4 Hz, 0.5 H), 3.39 (dt, J = 10.5, 7.2 Hz, 1 H), 3.26 (p, J = 6.6 Hz, 2 H), 2.28 (ddd, J = 16.2, 12.2, 5.0 Hz, 0.5 H), 2.20–1.95 (m, 2.5 H), 1.84 (ddt, J = 20.2, 12.8, 5.0 Hz, 3 H), 1.70–1.54 (m, 3 H), 1.51–1.42 (m, 3 H), 1.39 (br s, 8 H), 1.33–1.17 (m, 1 H); **^{13}C NMR** (76 MHz, Chloroform-*d*) δ 175.6, 175.4, 154.2, 153.9, 80.1, 79.6, 77.4, 68.0, 67.5, 52.2, 51.5, 51.4, 48.8, 48.7, 37.6, 36.3, 35.1, 34.0, 28.9, 28.8, 28.5, 28.5, 27.1, 26.9, 23.3, 23.2, 23.18, 22.9; **HRMS** (ESI^+) *m/z* calcd for $C_{16}H_{29}N_4O_4$ $[M+H]^+$, 341.2183; found, 341.2180.

To a mixture of 1-(*tert*-butyl) 2-methyl 2-(5-azidopentyl)pyrrolidine-1,2-dicarboxylate (7.45 g, 21.9 mmol, 1 eq) in methanol / water (1 : 1, 50 mL, 0.4 M), $LiOH{\cdot}H_2O$ (1.84 g, 43.8 mmol, 2 eq) was added.

The reaction mixture was stirred overnight at 60 °C, and extracted with diethyl ether (1x). The aqueous layer was acidified with 1 N HCl until pH 3 was achieved and extracted with ethyl acetate (3x). The combined organic layers were washed with brine, dried ($MgSO_4$), filtered, and evaporated in vacuo to give azide **10** (6.98 g, 27.4 mmol, 98%) as a pale-yellow, oily mixture of rotamers which solidified upon storage at -20 °C: **Mp** 55°C; **IR** (ATR, CH_2Cl_2) 2932, 2093, 1737, 1623, 1422, 1241, 1165 cm^{-1}; **^{1}H NMR** (300 MHz, Chloroform-*d*) δ 11.00 (s, 1 H), 3.68 (ddd, J = 12.2, 7.8, 4.8 Hz, 0.5 H), 3.50 (dt, J = 10.3, 5.3 Hz, 0.5 H), 3.42–3.25 (m, 1 H), 3.21 (app q, J = 6.3 Hz, 2 H), 2.43 (dt, J = 11.7, 4.9 Hz, 0.5 H), 2.21–1.94 (m, 2.5 H), 1.93–1.67 (m, 3 H), 1.56 (m, 2 H), 1.42 (br s, 4.5 H), 1.36 (br s, 6.5 H), 1.29–1.11 (m, 2 H); **^{13}C NMR** (76 MHz, Chloroform-*d*) δ 180.8, 177.0, 156.2, 154.0, 81.4, 80.5, 69.6, 67.4, 51.4, 51.3, 49.2, 48.7, 37.6, 35.6, 35.0, 34.0, 28.8, 28.7, 28.5, 28.4, 27.1, 26.8, 23.5, 23.1, 22.9, 22.8; **HRMS** (ESI^+) *m/z* calcd for $C_{15}H_{27}N_4O_4$ $[M+H]^+$, 327.2027; found, 327.2024.

2,3-Diaminobenzamide dihydrochloride (**11**).[1] To a flame-dried 100-mL round bottom flask charged with a solution of 2-amino-3-nitrobenzoic acid (2.00 g, 11.0 mmol, 1 eq) in dimethoxyethane (12 mL, 0.9 M), freshly distilled thionyl chloride (1.06 mL, 14.6 mmol, 1.3 eq) was added dropwise under a nitrogen atmosphere. The reaction mixture was warmed to 50 °C and stirred overnight under nitrogen. Subsequently, the mixture was concentrated under reduced pressure and the residue was added dropwise under vigorous stirring to a cold (10 °C) solution of 30% NH_4OH (9.4 mL, 22 eq). The reaction mixture was stirred at 50 °C for 1 h, diluted a third of the volume with water and stirred at room temperature for 20 min. An orange precipitate was filtered off, washed with water, and dried to give 2-amino-3-nitrobenzamide (2.00 g, 10.9 mmol, 99%) as an orange solid that was used in the next step without further purification: **^{1}H NMR** (300 MHz, DMSO-d_6) δ 8.47 (br s, 2 H), 8.18 (dd, J = 8.5, 1.5 Hz, 1 H), 8.14 (br s, 1 H), 7.95 (dd, J = 7.5, 1.6 Hz, 1 H), 7.61 (br s, 1 H), 6.68 (dd, J = 8.6, 7.5 Hz, 1 H).

A suspension of 2-amino-3-nitrobenzamide (3.80 g, 20.98 mmol, 1 eq) in MeOH (400 mL, 0.05 M) was treated portionwise with 10% (w/w) Pd-C catalyst (1.13 g, 5 mol%), stirred at room temperature for 12 h under an atmosphere of hydrogen gas, and filtered through celite. The filtrate was concentrated in vacuo and 3 N HCl in MeOH (30 mL) was added to the residue. The solvents were evaporated to afford **11** (4.4 g, 19.5 mmol, 93%) as an orange solid: **^{1}H NMR** (300 MHz, DMSO-d_6) δ 8.04 (br s, 1 H), 7.60 (dd, J = 8.0, 1.4 Hz, 1 H), 7.45 (br s, 1 H), 7.36 (dd, J = 7.8, 1.4 Hz, 1 H), 6.71 (t, J = 7.9 Hz, 1 H), 5.80 (br, 6 H).

***tert*-Butyl 2-((2-amino-3-carbamoylphenyl)carbamoyl)-2-(5-azidopentyl)pyrrolidine-1-carboxylate** (**12**). To a flame-dried 25-mL round bottom flask charged with a solution of **10** (0.80 g, 2.45 mmol, 1 eq) in dimethylformamide / pyridine (1 : 1, 4 mL, 0.6 M) was added at room temperature CDI (0.42 g, 2.57 mmol, 1.05 eq). The reaction mixture was stirred at 45 °C for 2 h under nitrogen, treated with amide **11**

[1] Barkalow, J. H.; Breting, J.; Gaede, B. J.; Haight, A. R.; Henry, R.; Kotecki, B.; Mei, J.; Pearl, K. B., Tedrow, J. S.; Viswanath, S. K. "Process Development for ABT-472, a Benzimidazole PARP Inhibitor." *Org. Proc. Res. Dev.* **2007**, *11*(4), 693-698.

(1.1 g, 4.90 mmol, 2 eq) and stirred at room temperature overnight under nitrogen. Brine was added and the mixture was extracted with ethyl acetate (3x). The combined organic layers were washed with brine (2x), dried ($MgSO_4$), filtered, and evaporated in vacuo. The crude residue was purified by chromatography on SiO_2 (20% hexanes in ethyl acetate to 100% ethyl acetate), affording amide **12** (0.86 g, 1.87 mmol, 76%) as a white, solid mixture of rotamers: **Mp** 170.5–172.3 °C; **IR** (ATR, CH_2Cl_2) 3435, 3320, 2933, 2866, 2090, 1677, 1642, 1392, 1366, 1162, 1142 cm^{-1}; **^{1}H NMR** (300 MHz, Chloroform-*d*) δ 9.11 (s, 1 H), 7.59 (d, *J* = 7.8 Hz, 1 H), 7.23 (d, *J* = 8.2 Hz, 1 H), 6.62 (t, *J* = 7.9 Hz, 1 H), 6.04 (br s, 3 H), 3.76–3.65 (m, 0.5 H), 3.61–3.48 (m, 0.5 H), 3.42–3.18 (m, 3 H), 2.79–2.66 (m, 0.5 H), 2.40–1.99 (m, 3.5 H), 1.89–1.75 (m, 2.5 H), 1.59 (app p, *J* = 7.0 Hz, 2.5 H), 1.49 (s, 9 H), 1.44–1.30 (m, 4 H); **^{13}C NMR** (76 MHz, Chloroform-*d*) δ 172.9, 171.9, 156.1, 143.4, 128.57, 125.3, 124.9, 115.7, 115.2, 81.2, 71.1, 51.4, 51.4, 49.8, 37.1, 35.3, 34.8, 28.8, 28.6, 28.5, 27.8, 26.9, 23.8, 22.8; **HRMS** (ESI$^+$) *m/z* calcd for $C_{22}H_{34}N_7O_4$ $[M+H]^+$, 460.2667; found, 460.2649.

2-(2-(5-Azidopentyl)pyrrolidin-2-yl)-1*H*-benzo[*d*]imidazole-4-carboxamide (**13**). A solution of amide **12** (0.86 g, 1.87 mmol) in glacial acetic acid (40 mL, 0.05 M) was heated at reflux for 8 h, concentrated to ½ the volume under reduced pressure, diluted with water and extracted with ethyl acetate. The water layer was basified using saturated aq. $KHCO_3$ and extracted with ethyl acetate (3x). The combined organic layers were dried ($MgSO_4$), filtered, and concentrated in vacuo. The residue was purified by chromatography on SiO_2 (5% methanol in dichloromethane) to yield **13** (0.51 g, 1.50 mmol, 80%) as a yellow solid: **Mp** 56.7–59.1 °C; **IR** (ATR, CH_2Cl_2) 3305, 3174, 2936, 2863, 2094, 1655, 1602, 1409, 1245 cm^{-1}; **^{1}H NMR** (500 MHz, Chloroform-*d*) δ 10.94 (br s, 1 H), 9.83 (s, 1 H), 8.11 (dd, *J* = 19.4, 12.5 Hz, 1 H), 7.65–7.57 (m, 1 H), 7.28 (t, *J* = 8.0 Hz, 1 H), 6.42 (s, 1 H), 3.17 (app q, *J* = 6.7 Hz, 3 H), 2.96 (ddd, *J* = 10.7, 7.7, 5.3 Hz, 1 H), 2.58 (ddd, *J* = 12.1, 7.8, 4.2 Hz, 1 H), 2.13 (ddd, *J* = 16.1, 12.0, 4.5 Hz, 1 H), 1.96–1.79 (m, 3 H), 1.76–1.67 (m, 1 H), 1.49 (h, *J* = 7.1 Hz, 2 H), 1.31 (qt, *J* = 17.1, 8.6 Hz, 4 H), 1.16–1.06 (m, 1 H); **^{13}C NMR** (126 MHz, Chloroform-*d*) δ 168.4, 161.4, 142.8, 134.3, 134.5, 123.6, 122.0, 115.1, 66.4, 51.4, 46.9, 41.4, 38.9, 28.7, 27.0, 25.9, 24.8; **HRMS** (ESI$^+$) *m/z* calcd for $C_{17}H_{24}N_7O$ $[M+H]^+$, 342.2037; found, 342.2030.

Allyl 2-(5-aminopentyl)-2-(4-carbamoyl-1*H*-benzo[*d*]imidazol-2-yl)pyrrolidine-1-carboxylate (**14**). A solution of **13** (0.20 g, 0.58 mmol, 1 eq) in dichloromethane / saturated aq. $NaHCO_3$ (1 : 1, 7 mL) was treated dropwise with allyl chloroformate (0.08 mL, 0.70 mmol, 1.2 eq) and stirred at room temperature for 1 h. The reaction mixture was partitioned between ethyl acetate and water and the water layer was extracted with ethyl acetate (2x). The combined organic layers were washed with brine, dried ($MgSO_4$), filtered, and concentrated in vacuo. The crude residue was purified by chromatography on SiO_2 (2% acetone in dichloromethane) to give the Alloc-protected pyrrolidine (0.21 g, 0.50 mmol, 85%) as a white, solid mixture of rotamers: **Mp** 166.1–167.8°C; **IR** (ATR, CH_2Cl_2) 3374, 2945, 2876, 2094, 1686, 1655,

1601, 1393, 1245 cm^{-1}; ^{1}**H NMR** (500 MHz, Chloroform-*d*) δ 11.48 (s, 1 H), 9.66 (d, *J* = 4.2 Hz, 1 H), 8.10 (dd, *J* = 7.6, 1.1 Hz, 1 H), 7.60 (dd, *J* = 8.1, 1.1 Hz, 1 H), 7.34 (t, *J* = 7.8 Hz, 1 H), 6.22 (d, *J* = 4.2 Hz, 1 H), 5.95 (ddt, *J* = 17.3, 10.7, 5.5 Hz, 1 H), 5.40–5.29 (m, 1 H), 5.24 (dd, *J* = 10.5, 1.4 Hz, 1 H), 4.73–4.60 (m, 2 H), 3.64 (ddd, *J* = 9.9, 6.1, 2.9 Hz, 1 H), 3.50 (td, *J* = 10.2, 7.3 Hz, 1 H), 3.44–3.34 (m, 1 H), 3.19 (t, *J* = 6.8 Hz, 2 H), 2.69–2.54 (m, 1 H), 2.15–2.01 (m, 2 H), 2.01–1.90 (m, 2 H), 1.53 (p, *J* = 7.1 Hz, 2 H), 1.44–1.22 (m, 3 H), 1.13–0.98 (m, 1 H); ^{13}**C NMR** (126 MHz, Chloroform-*d*) δ 167.9, 157.5, 156.3, 139.9, 134.6, 132.6, 123.9, 122.9, 122.5, 117.9, 115.2, 66.4, 66.4, 51.4, 49.4, 37.8, 35.8, 28.7, 26.8, 24.2, 23.1; **HRMS** (ESI^{+}) *m/z* calcd for $C_{21}H_{28}N_7O_3$ $[M+H]^+$, 426.2248; found, 426.2240.

To a solution of Alloc-protected pyrrolidine (0.17 g, 0.40 mmol) and PPh_3 (0.13 g, 0.48 mmol, 1.2 eq) in THF (4.5 mL, 0.09 M), was added H_2O (0.14 mL, 7.99 mmol, 20 eq). The reaction mixture was heated at 60 °C overnight and concentrated in vacuo. The crude residue was purified by chromatography on SiO_2 (20% MeOH in CH_2Cl_2 to a mixture of 19.7% MeOH / 1.6% NH_4OH in in CH_2Cl_2) to give **14** (0.15 g, 0.36 mmol, 91%) as a white, solid mixture of rotamers: **Mp** 87.0–89.1 °C; **IR** (ATR, CH_2Cl_2) 3297, 3165, 2930, 2861, 1665, 1603, 1399, 1335, 1245, 1118, 993 cm^{-1}; ^{1}**H NMR** (500 MHz, Chloroform-*d*) δ 11.54 (br s, 1 H), 9.67 (s, 1 H), 8.08 (d, *J* = 7.6 Hz, 1 H), 7.58 (d, *J* = 7.8 Hz, 1 H), 7.31 (t, *J* = 7.8 Hz, 1H), 6.32 (s, 1H), 5.93 (ddt, *J* = 16.3, 10.7, 5.5 Hz, 1H), 5.31 (d, *J* = 17.2 Hz, 1H), 5.22 (d, *J* = 10.5 Hz, 1 H), 4.69–4.58 (m, 2 H), 3.63 (ddd, *J* = 10.3, 6.9, 2.8 Hz, 1 H), 3.48 (td, *J* = 10.1, 6.9 Hz, 1 H), 3.32 (dt, *J* = 10.9, 4.1 Hz, 1 H), 2.63–2.58 (m, 2 H), 2.04 (td, *J* = 11.6, 7.6 Hz, 2 H), 2.00–1.81 (m, 3 H), 1.38 (m, 2 H), 1.27 (m, 4 H), 1.04 (ddt, *J* = 12.6, 9.1, 4.3 Hz, 1 H), 0.91–0.79 (m, 1 H); ^{13}**C NMR** (126 MHz, Chloroform-*d*) δ 168.0, 157.7, 156.2, 139.9, 134.6, 132.6, 123.8, 122.8, 122.4, 117.8, 115.2, 66.4, 66.3, 49.4, 42.0, 37.8, 35.8, 33.3, 31.7, 26.9, 24.4, 23.1, 22.7, 14.2; **HRMS** (ESI^{+}) *m/z* calcd for $C_{21}H_{30}N_5O_3$ $[M+H]^+$, 400.2343; found, 400.2335.

Allyl 2-(5-((*S*)-2-((*S*)-2-((*S*)-1-((2*S*,5*S*,*E*)-2-benzyl-5-((*tert*-butoxycarbonyl)amino)-7-methyloct-3-enoyl)pyrrolidine-2-carboxamido)-3-methylbutanamido)-5-(((benzyloxy)carbonyl)amino)pentanamido)pentyl)-2-(4-carbamoyl-1*H*-benzo[*d*]imidazol-2-yl)pyrrolidine-1-carboxylate (**16**). A flame-dried 10-mL round bottom flask was charged with a solution of acid **15** (30 mg, 0.04 mmol, 1 eq) in CH_2Cl_2 (0.4 mL, 0.1 M), and treated at 0°C with PFTU (18 mg, 0.041 mmol, 1.1 eq). The reaction mixture was stirred at room temperature for 30 min, treated with carbamate **14** (31 mg, 0.078 mmol, 2.1 eq), stirred overnight at room temperature, quenched with saturated aq. NH_4Cl and extracted with ethyl acetate (3x). The combined organic layers were washed with brine, dried ($MgSO_4$), filtered, and concentrated in vacuo. The crude residue was purified by chromatography on SiO_2 (5% MeOH in CH_2Cl_2) to afford **16** (41 mg, 0.03 mmol, 93%) as a white, solid mixture of rotamers: **Mp** 126.7–28.4 °C; **IR** (ATR, CH_2Cl_2) 3296, 2930, 1646, 1517, 1401, 1247, 1168 cm^{-1}; ^{1}**H NMR** (500 MHz, Chloroform-*d*) δ 11.50 (s, 1 H), 9.66 (s, 1 H), 8.07 (d, *J* = 7.6 Hz, 1 H), 7.85

(d, *J* = 17.1 Hz, 0.5 H), 7.57 (d, *J* = 7.9 Hz, 1 H), 7.40–7.12 (m, 10 H), 7.08 (d, *J* = 7.3 Hz, 2 H), 6.87-6.66 (m, 1 H), 6.60–6.44 (m, 1 H), 6.36-6.14 (m, 1 H), 5.93 (ddt, *J* = 16.4, 10.9, 5.5 Hz, 1 H), 5.68–5.53 (m, 1 H), 5.53–5.34 (m, 1.5 H), 5.30 (d, *J* = 17.1 Hz, 1 H), 5.22 (d, *J* = 10.4 Hz, 1 H), 5.02 (s, 2 H), 4.95-4.84 (m, 0.5 H), 4.71-4.55 (m, 2 H), 4.49–4.29 (m, 2 H), 4.23-4.14 (m, 0.5 H), 4.11-3.94 (m, 1 H), 3.68–3.58 (m, 1 H), 3.58–3.44 (m, 2 H), 3.35 (dd, *J* = 10.6, 5.6 Hz, 1 H), 3.31–2.94 (m, 6 H), 2.69 (dd, *J* = 13.6, 6.9 Hz, 1 H), 2.62-2.47 (m, 1 H), 2.32-2.09 (m, 2 H), 2.09–1.87 (m, 6.5 H), 1.86-1.68 (m, 3.5 H), 1.61 (dp, *J* = 17.6, 6.3, 5.7 Hz, 2 H), 1.55-1.33 (m, 12 H), 1.32-1.20 (m, 5 H), 1.16 (dt, *J* = 13.4, 6.8 Hz, 1 H), 1.08–0.98 (m, 1 H), 0.95-0.72 (m, 12 H); **^{13}C NMR** (126 MHz, Chloroform-*d*) δ 174.0, 173.9, 172.6, 172.5, 172.1, 171.8, 171.7, 171.6, 171.5, 171.3, 171.1, 170.8, 168.1, 168.0, 167.99, 157.7, 157.6, 156.8, 156.8, 156.6, 156.14, 156.11, 156.07, 155.2, 139.9, 139.4, 138.9, 137.0, 136.8, 136.0, 135.4, 134.6, 132.7, 129.3, 128.5, 128.1, 128.0, 127.4, 126.6, 126.4, 123.8, 122.7, 122.3, 117.8, 115.3, 79.7, 79.3, 66.6, 66.3, 61.4, 61.1, 61.1, 59.5, 59.5, 59.5, 57.4, 57.35, 50.7, 49.3, 47.4, 46.0, 44.1, 40.5, 38.7, 38.5, 35.9, 34.8, 34.6, 31.7, 29.8, 29.1, 28.5, 27.0, 26.8, 26.6, 26.0, 25.4, 25.0, 24.7, 24.6, 24.1, 23.1, 22.8, 22.6, 22.5, 22.0, 20.8, 19.4, 19.1, 17.8, 14.2; **HRMS** (ESI$^+$) *m/z* calcd for $C_{65}H_{91}N_{10}O_{11}$ [M+H]$^+$, 1187.6863; found, 1187.6828.

Benzyl ((4*S*)-4-((*S*)-2-((*S*)-1-((2*S*,5*S*,*E*)-2-benzyl-5-((*tert*-butoxycarbonyl)amino)-7-methyloct-3-enoyl)pyrrolidine-2-carboxamido)-3-methylbutanamido)-5-((5-(2-(4-carbamoyl-1*H*-benzo[*d*]imidazol-2-yl)pyrrolidin-2-yl)pentyl)amino)-5-oxopentyl)carbamate (**2**). A flame-dried 25-mL round bottom flask was charged with a solution of **16** (73 mg, 0.061 mmol, 1 eq) in CH_2Cl_2 (1.5 mL, 0.04 M) and treated with $PhSiH_3$ (0.18 mL, 1.47 mmol, 24 eq) under an argon atmosphere, followed by the dropwise addition of a solution of tetrakis(triphenylphosphine)palladium (0.60 mg, 0.66 μmol, 1 mol%) in CH_2Cl_2 (1 mL). The reaction mixture was stirred at room temperature for 1 h, filtered through a pad of celite® and concentrated in vacuo. The crude residue was purified by chromatography on SiO_2 (10% MeOH in CH_2Cl_2) to afford **2** (52 mg, 0.05 mmol, 77%) as a white, solid mixture of rotamers: **Mp** 138.5–141.2 °C; **IR** (ATR, CH_2Cl_2) 3288, 2958, 1646, 1526, 1392, 1366, 1246, 1166 cm^{-1}; **^{1}H NMR** (701 MHz, DMSO-*d*$_6$, 60 °C) δ 9.02 (s, 1 H), 7.93 (d, *J* = 8.5 Hz, 0.5 H), 7.83 (d, *J* = 7.5 Hz, 1 H), 7.76 (d, *J* = 7.8 Hz, 0.5 H), 7.67 (d, *J* = 8.0 Hz, 1.5 H), 7.59 (s, 0.5 H), 7.49 (s, 1 H), 7.42 (s, 1 H), 7.36–7.30 (m,, 4 H), 7.28 (app q, *J* = 7.8 Hz, 2 H), 7.23 (d, *J* = 7.5 Hz, 1 H), 7.17 (d, *J* = 7.8 Hz, 2.5 H), 7.11 (d, *J* = 7.3 Hz, 0.5 H), 7.00 (d, *J* = 7.7 Hz, 1.5 H), 6.49–6.30 (m, 1 H), 5.43 (qd, *J* = 15.7, 7.0 Hz, 1 H), 5.35 (dd, *J* = 15.7, 8.9 Hz, 0.5 H), 5.21–5.09 (m, 0.5 H), 5.00 (s, 2 H), 4.50 (d, *J* = 8.6 Hz, 0.5 H), 4.36 (d, *J* = 7.3 Hz, 0.5 H), 4.17 (q, *J* = 8.1 Hz, 1.5 H), 4.09 (t, *J* = 7.3 Hz, 0.5 H), 3.87 (t, *J* = 8.1 Hz, 0.5 H), 3.81–3.74 (m, 0.5 H), 3.45 (p, *J* = 7.8 Hz, 2 H), 3.37 (d, *J* = 10.1 Hz, 1 H), 2.97 (app dq, *J* = 13.3, 6.8 Hz, 6 H), 2.88 (d, *J* = 14.0 Hz, 1 H), 2.65 (dd, *J* = 13.9, 8.0 Hz, 1 H), 2.10 (q, *J* = 11.2, 10.6 Hz, 1 H), 2.03–1.92 (m, 3 H), 1.92–1.84 (m, 2 H), 1.77 (q, *J* = 11.6 Hz, 1 H), 1.74–1.57 (m, 4 H), 1.54–1.47 (m, 1 H), 1.46–1.40 (m, 2

H), 1.39–1.34 (m, 10 H), 1.32 (t, *J* = 8.0 Hz, 2.5 H), 1.28–1.23 (m, 2.5 H), 1.19 (t, *J* = 8.2 Hz, 3 H), 1.12 (dt, *J* = 13.8, 6.9 Hz, 1 H), 1.07–0.97 (m, 2 H), 0.89–0.72 (m, 13 H); **^{1}H NMR** (701 MHz, DMSO-d_6 + D_2O) δ 7.81 (d, *J* = 7.5 Hz, 1 H), 7.67 (d, *J* = 8.0 Hz, 1 H), 7.34–7.25 (m, 5 H), 7.21 (d, *J* = 7.4 Hz, 1 H), 7.17–7.11 (m, 3 H), 6.97 (d, *J* = 7.4 Hz, 1 H), 5.49–5.38 (m, 1 H), 5.32 (dd, *J* = 15.9, 9.1 Hz, 0.5 H), 5.18–5.04 (m, 0.5 H), 4.98 (s, 2 H), 4.47 (d, *J* = 8.8 Hz, 0.5 H), 4.30 (d, *J* = 7.5 Hz, 1 H), 4.18–4.09 (m, 1 H), 4.07–4.01 (m, 1 H), 3.84 (d, *J* = 8.6 Hz, 1 H), 3.78–3.70 (m, 0.5 H), 3.27–3.21 (m, 1 H), 3.21–3.14 (m, 1 H), 3.08–3.01 (m, 1 H), 3.02–2.90 (m, 5 H), 2.85 (d, *J* = 14.3 Hz, 0.5 H), 2.63 (t, *J* = 10.9 Hz, 1 H), 2.48–2.42 (m, 1 H), 2.14–2.05 (m, 2 H), 2.00–1.80 (m, 5 H), 1.80–1.57 (m, 5 H), 1.54–1.47 (m, 1 H), 1.44–1.26 (m, 14 H), 1.25–1.12 (m, 5 H), 1.13–1.05 (m, 1 H), 1.04–0.90 (m, 2 H), 0.88–0.68 (m, 12 H); **^{13}C NMR** (151 MHz, Chloroform-*d*) δ 173.9, 173.3, 172.8, 172.3, 172.0, 171.8, 171.5, 171.0, 168.1, 157.0, 156.9, 156.6, 155.3, 141.6, 139.3, 138.9, 136.8, 136.1, 135.4, 134.6, 129.3, 128.5, 128.4, 128.1, 128.0, 127.4, 126.9, 126.6, 126.4, 123.7, 122.3, 122.0, 115.6, 79.8, 79.3, 66.5, 61.4, 61.1, 59.8, 57.4, 53.4, 52.5, 50.8, 47.5, 47.1, 46.4, 46.0, 44.1, 40.5, 40.4, 39.2, 38.5, 38.22, 38.1, 34.8, 34.6, 32.0, 31.7, 29.8, 29.5, 28.8, 28.5, 26.6, 26.0, 25.4, 25.0, 24.8, 24.5, 24.4, 23.2, 22.8, 22.6, 22.2, 19.5, 19.2, 18.0, 14.3, 14.2, 11.6; **HRMS** (ESI^+) *m/z* calcd for $C_{61}H_{87}N_{10}O_9$ $[M+H]^+$, 1103.6652; found, 1103.6636.

4-(((*tert*-Butyldimethylsilyl)oxy)methyl)phenyl (*tert*-butoxycarbonyl)-*L*-alaninate (**17**). A solution of 4-(hydroxymethyl)phenol (2.00 g, 16.0 mmol, 1 eq) in dimethylformamide (7 mL, 2.3 M) was treated with imidazole (1.30 g, 19.2 mmol, 1.2 eq) and TBSCl (2.98 g, 19.2 mmol, 1.2 eq) at 0 °C. The reaction mixture was stirred at room temperature for 1.5 h, diluted with Et_2O and washed with saturated aq. NH_4Cl (1x) and brine (3x). The organic layer was dried (Na_2SO_4), filtered, and concentrated in vacuo. The crude residue was purified by chromatography on SiO_2 (5% ethyl acetate in hexanes) to give 4-(((*tert*-butyldimethylsilyl)oxy)methyl)phenol (3.50 g, 14.6 mmol, 91%) as a colorless oil: **^{1}H NMR** (300 MHz, Chloroform-*d*) δ 7.23–7.16 (m, 2H), 6.82–6.75 (m, 2 H), 4.68 (s, 1 H), 4.66 (s, 2 H), 0.93 (s, 9 H), 0.09 (s, 6 H).

A solution of 4-(((*tert*-butyldimethylsilyl)oxy)methyl)phenol (151 mg, 0.63 mmol, 1.2 eq), N-Boc-*L*-alanine (100 mg, 0.53 mmol, 1 eq), and DMAP (6.4 mg, 0.05 mmol, 10 mol%) in THF (1 mL, 0.5 M), was treated at room temperature with DCC (142 mg, 0.63 mmol, 1.2 eq), stirred overnight at room temperature, and filtered. The filtrate was diluted with Et_2O (50 mL) and washed with 0.3 M NaOH (3x), water (1x) and brine (1x). The organic layer was dried (Na_2SO_4), filtered, and concentrated in vacuo. The crude residue was purified by chromatography on SiO_2 (10% acetone in hexanes) to give **17** (0.20 g, 0.52 mmol, 97%) as a colorless oil: **^{1}H NMR** (300 MHz, Chloroform-*d*) δ 7.37–7.29 (m, 2 H), 7.09–7.02 (m, 2 H), 4.73 (s, 2 H), 5.15–5.02 (m, 0.5 H), 4.73 (s, 2 H), 4.60–4.47 (m, 0.5 H) 1.56 (s,, 1.5 H), 1.54 (s, 1.5 H), 1.46 (s, 9 H), 0.94 (s, 9 H), 0.10 (s, 6 H).

4-(((*tert*-Butoxycarbonyl)-*L*-alanyl)oxy)benzyl 2-(7-carbamoyl-1*H*-benzo[*d*]imidazol-2-yl)-2-methylpyrrolidine-1-carboxylate (**7**). A solution of **17** (0.21 g, 0.51 mmol, 1 eq) in THF (1 mL, 0.5 M) was treated dropwise with TBAF (1.02 mL, 1.02 mmol, 1 M solution in THF, 2 eq). The reaction mixture was stirred at room temperature for 30 min, quenched with H_2O, extracted with Et_2O (3x), washed with brine, dried (Na_2SO_4), filtered, and concentrated in vacuo. The resulting crude alcohol was directly used in the next step.

A flame-dried 25-mL flask was charged with a solution of the crude alcohol (0.12 g, 0.41 mmol, 1 eq) and DIPEA (134 µL, 0.81 mmol, 2 eq) in CH_2Cl_2 (3 mL, 0.14 M), and bis(4-nitrobenzene)carbonate (0.25g, 0.81 mmol, 2 eq) was added portion wise at 0 °C. The reaction mixture was allowed to warm up to room temperature and stirred overnight under nitrogen, concenctrated in vacuo, and purified by chromatography on SiO_2 (2% acetone in CH_2Cl_2) to yield 4-((((4-nitrophenoxy)carbonyl)oxy)methyl)phenyl (*tert*-butoxycarbonyl)-*L*-alaninate as a colorless oil containing p-nitrophenol as impurity: **^{1}H NMR** (601 MHz, Chloroform-*d*) δ 8.28 (d, J = 9.1 Hz, 2 H), 7.46 (d, J = 8.1 Hz, 2 H), 7.38 (d, J = 9.1 Hz, 2 H), 7.13 (d, J = 8.4 Hz, 2 H), 6.73 (s, 1 H), 5.28 (s, 2 H), 4.58–4.49 (m, 1 H), 1.58 (d, J = 7.2 Hz, 3 H), 1.48 (s, 9 H).

A flame-dried 10-mL flask was charged with a solution of crude 4-((((4-nitrophenoxy)carbonyl)oxy)methyl)phenyl (*tert*-butoxycarbonyl)-*L*-alaninate (35 mg, 0.08 mmol, 1 eq) in DMF (0.8 mL, 0.1 M), veliparib (28 mg, 0.11 mmol, 1.5 eq) and DIPEA (12 µL, 0.08 mmol, 1 eq). The reaction mixture was stirred at room temperature for 48 h under nitrogen, and partitioned between ethyl acetate and brine. The organic layer was washed with brine (4x), dried ($MgSO_4$), filtered, and concentrated in vacuo. The crude residue was purified by chromatography on SiO_2 (2% MeOH in CH_2Cl_2 to 4% MeOH in CH_2Cl_2) to give **7** (20 mg, 0.04 mmol, 47%) as a white, solid mixture of rotamers: **Mp** 115.0–118.6 °C; **IR** (ATR, CH_2Cl_2) 3284, 2979, 1686, 1606, 1509, 1414, 1164 cm^{-1}; **^{1}H NMR** (500 MHz, DMSO-d_6) δ 12.76–12.74 (m, 1 H), 9.27 (s, 1 H), 7.84–7.79 (m, 1 H), 7.68 (s, 1 H), 7.63 (d, J = 7.9 Hz, 0.5 H), 7.59–7.48 (m, 1 H), 7.42 (d, J = 8.1 Hz, 1 H), 7.28 (t, J = 7.8 Hz, 1 H), 7.08 (d, J = 8.4 Hz, 1 H), 6.73 (t, J = 6.9 Hz, 0.5 H), 6.62 (t, J = 6.9 Hz, 0.5 H), 5.09–4.79 (m, 2 H), 4.26–4.14 (m, 1 H), 3.79 (ddd, J = 16.9, 10.0, 7.2 Hz, 1 H), 3.70 – 3.60 (m, 1 H), 2.20 (app h, J = 6.4, 5.8 Hz, 1 H), 2.13 (dt, J = 12.8, 6.6 Hz, 0.5 H) 1.97 (dd, J = 13.6, 8.2 Hz, 2 H), 1.89-1.84 (m, 2.5 H), 1.54–1.46 (m, 0.5 H), 1.43-1.38 (m, 8 H), 1.37–1.33 (m, 1.5 H), 1.32–1.17 (m, 2 H), 1.15–1.00 (m, 0.5 H), 0.89–0.81 (m, 2 H); **^{13}C NMR** (126 MHz, DMSO-d_6) δ 172.1, 166.2, 160.0, 159.7, 155.4, 153.1, 153.0, 140.6, 140.5, 134.9, 134.8, 134.7, 134.5, 134.0, 131.7, 128.8, 127.8, 122.2, 122.1, 121.6, 121.56, 121.5, 120.8, 114.8, 78.4, 65.2, 62.1, 61.6, 54.9, 49.3, 49.2, 47.7, 42.9, 41.7, 34.2, 33.9, 30.9, 28.5, 28.2, 24.8, 23.9, 22.7, 22.6, 22.0, 20.6, 18.6, 16.6, 14.0, 11.2; **HRMS** (ESI$^+$) *m/z* calcd for $C_{29}H_{36}N_5O_7$ [M+H]$^+$, 566.2609; found, 566.2601.

4-((((*S*)-2-((*S*)-2-((*S*)-1-((2*S*,5*S*,*E*)-2-Benzyl-5-((*tert*-butoxycarbonyl)amino)-7-methyloct-3-enoyl)pyrrolidine-2-carboxamido)-3-methylbutanamido)-5-(((benzyloxy)carbonyl)amino)pentanoyl)-*L*-alanyl)oxy)benzyl 2-(4-carbamoyl-1*H*-benzo[*d*]imidazol-2-yl)-2-methylpyrrolidine-1-carboxylate (**4**). A solution of **7** (10 mg, 0.18 mmol) in CH_2Cl_2 (0.5 mL, 0.04 M) was treated dropwise at 0 °C with TFA (100 µL), stirred at room temperature for 1.5 h, concentrated in vacuo, and treated with acid **15** (14.2 mg, 0.18 mmol, 1 eq) followed by DMF (0.3 mL, 0.06 M), HATU (8.1 mg, 0.21 mmol, 1.2 eq), DIPEA (5.8 µL, 0.04 mmol, 2 eq) and HOAt (0.2 mg, 2 µmol, 10 mol%). The reaction mixture was stirred at room temperature for 1.5 h under nitrogen, diluted with ethyl acetate, washed with brine (5x), dried (Na_2SO_4), filtered, and concentrated in vacuo. The residue was purified by chromatography on SiO_2 (5% MeOH in CH_2Cl_2) to give **4** (11 mg, 9.0 µmol, 51%) as a white, solid mixture of rotamers: **Mp** 176.8–180.2 °C; **IR** (ATR, CH_2Cl_2) 3298, 2956, 1650, 1509, 1403, 1245, 1164 cm^{-1}; **^{1}H NMR** (400 MHz, DMSO-d_6, 60 °C) δ 12.53 (s, 1 H), 9.21 (s, 0.5 H), 8.34–8.20 (m, 1 H), 7.90 (d, *J* = 7.9 Hz, 0.5 H), 7.82 (d, *J* = 7.4 Hz, 1 H), 7.68–7.57 (m, 1 H), 7.53 (s, 0.5 H), 7.42 (s, 1 H), 7.37–7.21 (m, 7 H), 7.20–7.14 (s, 2.5 H), 7.13–7.05 (m, 1.5 H), 7.04–6.97 (m, 1.5 H), 7.02 (s, 2 H), 6.85–6.63 (m, 2 H), 6.41 (s, 0.5 H), 5.53–5.31 (m, 1.5 H), 5.16–5.06 (m, 0.5 H), 5.00 (s, 2.5 H), 4.86 (s, 0.5 H), 4.55–4.29 (m, 3 H), 4.27–4.12 (m, 1 H), 3.93–3.84 (m, 0.5 H), 3.83–3.73 (m, 1 H), 3.70–3.60 (m, 1 H), 3.53–3.42 (m, 1.5 H), 3.05–2.93 (m, 3 H), 2.65 (dd, *J* = 13.4, 7.9 Hz, 1 H), 2.35–2.22 (m, 1 H), 2.21–2.07 (m, 1.5 H), 2.05–1.93 (m, 4 H), 1.88 (s, 3 H), 1.84–1.64 (m, 4 H), 1.61–1.40 (m, 7.5 H), 1.37 (s, 9 H), 1.31–1.18 (m, 3.5 H), 1.17–1.00 (m, 2 H), 0.93–0.72 (m, 12 H); **^{13}C NMR** (151 MHz, DMSO-d_6) δ 171.6, 171.4, 171.2, 171.0, 170.8, 170.7, 166.3, 160.0, 159.7, 156.1, 154.8, 154.7, 153.5, 153.1, 150.0, 149.5, 139.3, 139.1, 137.2, 134.8, 134.6, 134.1, 129.1, 128.7, 128.3, 128.0, 127.8, 127.7, 127.3, 126.0, 125.8, 122.1, 121.5, 120.9, 114.9, 77.5, 77.4, 65.2, 65.1, 62.1, 61.6, 59.1, 58.9, 57.5, 50.1, 48.5, 48.3, 47.9, 47.7, 46.8, 46.6, 43.7, 42.9, 41.7, 38.5, 37.8, 34.2, 31.9, 30.6, 30.3, 29.4, 28.2, 26.4, 25.8, 24.2, 23.9, 22.7, 22.6, 22.4, 22.3, 22.0, 19.4, 19.2, 18.4, 17.8, 16.5; **HRMS** (ESI$^+$) *m/z* calcd for $C_{68}H_{89}N_{10}O_{13}$ $[M+H]^+$, 1253.6605; found, 1253.6559.

(3-(((2*S*)-1-(4-(((2-(7-Carbamoyl-1*H*-benzo[*d*]imidazol-2-yl)-2-methylpyrrolidine-1-carbonyl)oxy)methyl)phenoxy)-1-oxopropan-2-yl)amino)-3-oxopropyl)triphenylphosphonium chloride (**5**). A solution of **7** (10 mg, 0.18 mmol) in CH_2Cl_2 (0.5 mL, 0.04 M) was treated dropwise at 0 °C with TFA (100 µL), stirred for 1.5 h at room temperature, concentrated in vacuo, and treated sequentially with (2-carboxyethyl)triphenylphosphonium chloride **18** (6.7 mg, 0.18 mmol, 1 eq), DMF (0.3 mL, 0.06 M), HATU (8.1 mg, 0.21 mmol, 1.2 eq), DIPEA (5.8 µL, 0.04 mmol, 2 eq) and HOAt (0.2 mg, 2 µmol, 10 mol%). The reaction mixture was stirred at room temperature for 1.5 h under nitrogen, diluted with ethyl acetate, washed with brine (5x), dried (Na_2SO_4), filtered, and concentrated in vacuo. The residue was purified by chromatography on SiO_2 (6% MeOH in CH_2Cl_2) to give **5** (10 mg, 0.01

mmol, 66%) as a white, solid mixture of rotamers: **Mp** 163.5–166.1 °C; **IR** (ATR, CH_2Cl_2) 3408, 3283, 3062, 2937, 1662, 1607, 1529, 1509, 1439, 1405, 1195, 1165, 1113, 838 cm^{-1}; **^{1}H NMR** (400 MHz, DMSO-*d*$_6$) δ 12.75 (s, 0.5 H), 12.66 (s, 0.5 H), 9.26 (s, 1 H), 8.62 (dd, *J* = 15.2, 6.4 Hz, 1 H), 7.93–7.86 (m, 3.5 H), 7.86–7.73 (m, 15.5 H), 7.70–7.67 (m, 1 H), 7.63 (d, *J* = 8.0 Hz, 0.5 H), 7.58 (d, *J* = 7.9 Hz, 0.5 H), 7.41 (d, *J* = 8.2 Hz, 1 H), 7.27 (d, *J* = 7.7 Hz, 1 H), 7.07 (d, *J* = 8.4 Hz, 1 H), 6.75 (d, *J* = 8.1 Hz, 1 H), 6.61 (d, *J* = 8.5 Hz, 1 H), 5.03 (d, *J* = 10.1 Hz, 0.5 H), 4.93–4.78 (m, 0.5 H), 4.41–4.30 (m, 1 H), 3.87–3.73 (m, 2.5 H), 3.69–3.60 (m, 0.5 H), 2.69–2.65 (m, 0.5 H), 2.33 (dq, *J* = 3.3, 1.8 Hz, 0.5 H), 2.24–2.18 (m, 1 H), 2.01–1.92 (m, 2 H), 1.87 (d, *J* = 5.3 Hz, 3 H), 1.36 (dd, *J* = 14.4, 7.3 Hz, 3 H); **^{13}C NMR** (151 MHz, DMSO-*d*$_6$) δ 171.4, 171.2, 169.0, 168.86, 166.3, 160.0, 159.7, 153.5, 153.1, 149.8, 149.4, 140.6, 140.5, 135.0, 134.95, 134.8, 133.7, 133.7, 130.3, 130.2, 128.7, 127.8, 122.2, 121.7, 121.6, 121.5, 120.8, 118.6, 118.0, 114.9, 65.2, 62.1, 61.7, 48.5, 48.2, 47.7, 42.9, 41.7, 34.2, 30.9, 27.1, 23.9, 22.7, 22.6, 22.1, 16.8, 16.7, 16.5, 14.0; **HRMS** (ESI$^+$) *m/z* calcd for $C_{45}H_{45}PN_5O_6$ [M+H]$^+$, 782.3102; found, 782.3076.

(*S*)-2-((*S*)-2-((*S*)-1-((2*S*,5*S*,*E*)-2-Benzyl-5-((*tert*-butoxycarbonyl)amino)-7-methyloct-3-enoyl)pyrrolidine-2-carboxamido)-3-methylbutanamido)-5-(((benzyloxy)carbonyl)amino)pentanoic acid (**19**). A flame-dried 10-mL flask was charged with a solution of acid **15** (46.9 mg, 0.06 mmol, 1 eq), 4-aminobenzyl alcohol (15 mg, 0.12 mmol, 2 eq), and HOAt (8 mg, 0.06 mmol, 1 eq) in DMF (0.8 mL, 1 M), followed by the dropwise addition of DIPEA (15 µL, 0.09 mmol, 1.5 eq). The reaction mixture was cooled to 0 °C, treated with EDC (12 mg, 0.06 mmol, 1 eq), stirred at room temperature overnight under nitrogen, diluted with ethyl acetate, washed with aq. 1 N HCl (1x), saturated aq. $NaHCO_3$ (1x), brine (1x), dried ($MgSO_4$), and concentrated in vacuo. The crude residue was purified by chromatography on SiO_2 (50% ethyl acetate in hexanes to 100% ethyl acetate) to give **19** (49 mg, 0.05 mmol, 92%) as a pale-yellow, solid mixture of rotamers: **IR** (ATR, CH_2Cl_2) 3298, 2958, 1648, 1515, 1454, 1414, 1392, 1366, 1253, 1168 cm^{-1}; **^{1}H NMR** (601 MHz, DMSO-*d*$_6$) δ 9.97–9.66 (m, 1 H), 8.35-8.21 (m, 0.5 H), 7.61 (dd, *J* = 14.1, 8.2 Hz, 1 H), 7.55–7.49 (m, 1 H), 7.36–7.31 (m, 4 H), 7.29 (ddt, *J* = 11.1, 8.1, 3.7 Hz, 2 H), 7.23 (dt, *J* = 9.5, 2.6 Hz, 3 H), 7.16 (ddd, *J* = 11.2, 6.2, 2.1 Hz, 2.5 H), 7.11 (ddd, *J* = 14.2, 8.2, 6.1 Hz, 0.5 H), 6.99 (dd, *J* = 9.4, 7.4 Hz, 1 H), 6.73–6.64 (m, 1 H), 5.50–5.28 (m, 2 H), 5.17–5.04 (m, 1 H), 4.99 (d, *J* = 2.5 Hz, 2 H), 4.56–4.48 (m, 0.5 H), 4.43 (d, *J* = 4.0 Hz, 2 H), 4.40–4.30 (m, 1.5 H), 4.27 (dd, *J* = 8.6, 7.0 Hz, 0.5 H), 4.23– 4.06 (m, 1 H), 3.84 (p, *J* = 8.4, 7.7 Hz, 0.5 H), 3.80–3.72 (s, 0.5 H), 3.46 (dq, *J* = 14.6, 7.4 Hz, 2 H), 3.26–3.19 (m, 1 H), 3.01 (dq, *J* = 13.1, 6.6 Hz, 2 H), 2.92 (dd, *J* = 13.3, 7.4 Hz, 1 H), 2.85 (ddd, *J* = 13.7, 10.0, 4.1 Hz, 0.5 H), 2.68–2.59 (m, 1 H), 2.10 (qd, *J* = 8.8, 5.7 Hz, 0.5 H), 2.04–1.91 (m, 1.5 H), 1.89– 1.83 (m, 0.5 H), 1.82–1.64 (m, 4 H), 1.64–1.55 (m, 1 H), 1.54–1.46 (m, 1 H), 1.46–1.38 (m, 1 H), 1.38– 1.28 (m, 9.5 H), 1.27–1.16 (m, 1 H), 1.15–1.02 (m, 1.5 H), 0.93–0.79 (m, 6 H), 0.79–0.66 (m, 6 H); **^{13}C NMR** (151 MHz, DMSO-*d*$_6$) δ 172.0, 171.4, 171.3, 171.2, 171.0, 170.9, 170.86, 170.3, 170.2,

156.1, 154.8, 154.7, 139.2, 139.1, 137.6, 137.5, 137.5, 137.4, 137.4, 137.3, 137.2, 137.2, 129.2, 128.3, 128.0, 127.8, 127.7, 127.69, 127.3, 126.9, 126.8, 126.8, 126.0, 125.8, 118.9, 118.8, 77.4, 65.1, 65.1, 62.6, 58.9, 57.8, 57.6, 53.3, 53.2, 50.1, 48.3, 46.8, 46.6, 44.2, 44.1, 37.8, 30.5, 30.4, 29.4, 28.3, 28.2, 26.2, 26.1, 24.2, 23.9, 23.85, 22.5, 22.4, 22.1, 19.3, 19.2, 19.16, 18.8, 18.6, 18.4, 18.2, 17.9; **HRMS** (ESI$^+$) *m/z* calcd for $C_{51}H_{71}N_6O_9$ [M+H]$^+$, 911.5277; found, 911.5258.

4-((*S*)-2-((*S*)-2-((*S*)-1-((2*S*,5*S*,*E*)-2-Benzyl-5-((*tert*-butoxycarbonyl)amino)-7-methyloct-3-enoyl)pyrrolidine-2-carboxamido)-3-methylbutanamido)-5-(((benzyloxy)carbonyl)amino)pentanamido)benzyl 2-(7-carbamoyl-1*H*-benzo[*d*]imidazol-2-yl)-2-methylpyrrolidine-1-carboxylate (**3**). A solution of **19** (47 mg, 0.05 mmol) and bis(4-nitrophenyl)carbonate (77 mg, 0.07 mmol, 1.5 eq.) in CH_2Cl_2 (1.2 mL, 0.04 M) was treated at 0 °C with DIPEA (9 µL, 0.05 mmol, 1 eq), stirred at room temperature for 24 h, concentrated under reduced pressure and purified by chromatography on SiO_2 (2% MeOH in CH_2Cl_2 to 5 % MeOH in CH_2Cl_2) to give benzyl ((*S*)-4-((*S*)-2-((*S*)-1-((2*S*,5*S*,*E*)-2-benzyl-5-((*tert*-butoxycarbonyl)amino)-7-methyloct-3-enoyl)pyrrolidine-2-carboxamido)-3-methylbutanamido)-5-((4-((((4-nitrophenoxy)carbonyl)oxy)methyl)phenyl)amino)-5-oxopentyl)carbamate (46 mg, 0.04 mmol, 83%) as a white, solid mixture of rotamers: **Mp** 105.1–107.8 °C; **IR** (ATR, CH_2Cl_2) 3298, 2959, 1677, 1648, 1524, 1251, 1213 cm^{-1}; **^{1}H NMR** (601 MHz, DMSO-d_6) δ 10.17–9.83 (m, 1 H), 8.31 (d, *J* = 8.6 Hz, 2 H), 7.72 (t, *J* = 8.0 Hz, 1 H), 7.68–7.60 (m, 1 H), 7.56 (dd, *J* = 8.9, 4.8 Hz, 2H), 7.41 (d, *J* = 8.1 Hz, 2 H), 7.36–7.26 (m, 5 H), 7.22 (d, *J* = 7.2 Hz, 1 H), 7.17 (d, *J* = 7.8 Hz, 2 H), 6.99 (t, *J* = 8.0 Hz, 1 H), 6.74–6.64 (m, 1 H), 5.51–5.29 (m, 1.5 H), 5.24 (s, 2 H), 5.11–5.04 (m, 0.5 H), 4.99 (s, 2 H), 4.56–4.49 (m, 0.5 H), 4.42–4.26 (m, 2 H), 4.23–4.05 (m, 1 H), 3.90–3.81 (m, 0.5 H), 3.80–3.71 (m, 0.5 H), 3.53–3.41 (m, 2 H), 3.22 (dd, *J* = 13.3, 7.2 Hz, 1 H), 3.08–2.96 (m, 2 H), 2.96–2.89 (m, 1 H), 2.85 (t, *J* = 12.1 Hz, 0.5H), 2.63 (dt, *J* = 14.1, 7.2 Hz, 1H), 2.11 (t, *J* = 10.0 Hz, 0.5 H), 2.05–1.89 (m, 1.5 H), 1.88–1.82 (m, 1 H), 1.82–1.65 (m, 3.5 H), 1.60 (d, *J* = 11.4 Hz, 2 H), 1.55–1.46 (m, 1.5 H), 1.46–1.39 (m, 1 H), 1.38–1.28 (m, 9 H), 1.27–1.16 (m, 2.5 H), 1.15–1.00 (m, 1.5 H), 0.92–0.79 (m, 6.5 H), 0.79–0.64 (m, 5.5 H); **^{13}C NMR** (151 MHz, DMSO-d_6) δ 172.0, 171.5, 171.3, 171.0, 170.7, 170.6, 156.1, 155.3, 154.8, 154.7, 152.0, 145.2, 139.4, 139.3, 139.2, 139.1, 137.2, 134.5, 134.2, 134.0, 129.5, 129.4, 129.2, 128.3, 128.0, 127.8, 127.7, 127.3, 126.0, 125.8, 125.4, 122.6, 119.2, 119.1, 77.5, 77.4, 70.2, 65.1, 59.1, 58.9, 58.7, 57.6, 53.4, 53.3, 50.2, 50.16, 48.8, 48.3, 46.8, 46.6, 43.7, 43.7, 43.6, 38.5, 37.8, 34.2, 33.9, 31.9, 31.0, 30.6, 30.4, 29.2, 28.3, 28.2, 26.2, 24.8, 24.2, 23.9, 23.8, 22.5, 22.3, 22.1, 20.6, 19.3, 19.2, 18.4, 18.2, 17.9, 14.0, 11.3; **HRMS** (ESI$^+$) *m/z* calcd for $C_{58}H_{74}N_7O_{13}$ [M+H]$^+$, 1076.5339; found, 1076.5322.

A solution of benzyl ((*S*)-4-((*S*)-2-((*S*)-1-((2*S*,5*S*,*E*)-2-benzyl-5-((*tert*-butoxycarbonyl)amino)-7-methyloct-3-enoyl)pyrrolidine-2-carboxamido)-3-methylbutanamido)-5-((4-((((4-nitrophenoxy)carbonyl)oxy)methyl)phenyl)amino)-5-oxopentyl)carbamate (33 mg, 0.03 mmol, 1 eq) in

DMF (0.8 mL, 0.04 M) was treated with veliparib (11 mg, 0.05 mmol, 1.5 eq) and DIPEA (5 μL, 0.03 mmol, 1 eq), stirred at room temperature for 48 h, and partitioned between ethyl acetate and brine. The organic layer was washed with brine (4x), dried ($MgSO_4$), filtered, and concentrate in vacuo. The crude residue was purified by chromatography on SiO_2 (2% MeOH in CH_2Cl_2 to 4% MeOH in CH_2Cl_2) to give **3** (24 mg, 0.02 mmol, 67%) as a white, solid mixture of diastereomers: **Mp** 123.4–127.5 °C; **IR** (ATR, CH_2Cl_2) 3301, 2958, 2928, 2872, 1657, 1517, 1414, 1247, 1164 cm^{-1}; **^{1}H NMR** (400 MHz, DMSO-*d*$_6$, 80 °C) δ 12.39 (s, 1 H), 9.18 (s, 1 H), 8.04-7.87 (m, 0.5 H), 7.86-7.76 (m, 1.5 H), 7.64-7.54 (m, 1.5 H), 7.53-7.45 (m, 1 H), 7.37-7.30 (m, 4 H), 7.30–7.20 (m, 5 H), 7.20-7.09 (m, 3.5 H), 7.06-6.99 (m, 1 H), 6.97-6.86 (m, 1 H), 6.29-6.19 (m, 1 H), 5.56-5.32 (m, 1.5 H), 5.05-4.95 (m, 2.5 H), 4.93-4.79 (m, 1 H), 4.55-4.33 (m, 2 H), 4.29-4.09 (m, 1 H), 3.93-3.83 (m, 0.5 H), 3.82–3.73 (m, 1 H), 3.68-3.58 (m, 1 H), 3.54-3.35 (m, 2.5 H), 3.33-3.22 (m, 1 H), 2.67 (dd, *J* = 13.6, 8.1 Hz, 1 H), 2.34-2.21 (m, 1 H), 2.20-2.10 (m, 1 H), 2.08-1.91 (m, 4 H), 1.90-1.83 (m, 3.5 H), 1.82-1.71 (m, 3 H), 1.70-1.60 (m, 2 H), 1.58-1.40 (m, 3.5 H), 1.39-1.31 (m, 9.5 H), 1.29-1.21 (m, 3.5 H), 1.18-1.01 (m, 2 H), 0.95–0.83 (m, 6.5 H), 0.83–0.58 (m, 7.5 H); **^{13}C NMR** (151 MHz, DMSO-*d*$_6$) δ 171.4, 171.0, 170.4, 170.2, 166.3, 159.9, 159.7, 156.1, 154.8, 154.7, 153.6, 153.2, 140.6, 139.2, 139.1, 137.2, 134.8, 129.2, 128.3, 128.0, 127.8, 127.7, 127.69, 127.5, 127.3, 126.0, 125.8, 122.1, 121.6, 119.1, 118.6, 114.5, 77.5, 77.3, 65.6, 65.1, 62.1, 61.6, 58.9, 57.5, 50.2, 48.4, 48.3, 47.6, 46.6, 43.6, 43.0, 42.7, 37.8, 28.2, 26.1, 24.2, 23.9, 22.7, 22.6, 22.3, 22.0, 19.3, 19.2, 18.4, 17.9, 14.0; **HRMS** (ESI$^+$) *m/z* calcd for $C_{65}H_{85}N_{10}O_{11}$ $[M+H]^+$, 1181.6394; found, 1181.6389.

4-((*S*)-2-((*tert*-Butoxycarbonyl)amino)propanamido)benzyl 2-(4-carbamoyl-1*H*-benzo[*d*]imidazol-2-yl)-2-methylpyrrolidine-1-carboxylate (6). A flame-dried 10-mL flask was charged with a solution of 4-aminobenzyl alcohol (100 mg, 0.80 mmol, 1 eq), N-Boc-*L*-alanine (0.15 g, 0.80 mmol, 1 eq), and 1-hydroxy-7-azabenzotriazole (0.12 g, 0.87 mmol, 1.1 eq) in DMF (1.6 mL, 0.5 M), followed by dropwise addition of DIPEA (0.2 mL, 1.19 mmol, 1.5 eq). The reaction mixture was cooled to 0°C and EDCI (0.168 g, 0.87 mmol, 1.1 eq) was added portion wise. The solution was allowed to warm to room temperature, stirred overnight under nitrogen, diluted with ethyl acetate, washed with aq. 1 N HCl (1x), saturated aq. $NaHCO_3$ (1x), and brine (1x), dried ($MgSO_4$), filtered, and concentrated in vacuo to give crude **20** as a colorless oil that was directly used in the next step: **^{1}H NMR** (500 MHz, Chloroform-*d*) δ 8.50 (s, 1 H), 8.01 (s, 1 H), 7.49 (d, *J* = 8.5 Hz, 2H), 7.29 (d, *J* = 8.1 Hz, 2 H), 4.63 (s, 2 H), 4.37–4.25 (m, 1 H), 1.82 (s, 1 H), 1.46 (s, 9 H), 1.42 (d, *J* = 5.6 Hz, 3 H).

A solution of crude **20** (0.2 g, 0.68 mmol, 1 eq) and bis(4-nitrophenyl)carbonate (0.83 g, 2.72 mmol, 4 eq) in CH_2Cl_2 (8 mL, 0.08 M) was treated at 0 °C with DIPEA (355 μL, 2.04 mmol, 3 eq), stirred at room temperature for 48 h under nitrogen, concentrated in vacuo, and purified by chromatography on SiO_2 (25% ethyl acetate in hexanes) to afford *tert*-butyl (*S*)-(1-((4-((((4-nitrophenoxy)carbonyl)oxy)methyl)phenyl)amino)-1-oxopropan-2-yl)carbamate (42 mg, 0.09 mmol, 13

% (two steps) as a white solid: **^{1}H NMR** (500 MHz, Chloroform-*d*) δ 8.76 (s, 1 H), 8.26 (d, *J* = 9.2 Hz, 2 H), 7.53 (d, *J* = 8.1 Hz, 2 H), 7.38–7.32 (m, 4 H), 5.22 (s, 2 H), 5.13 (d, *J* = 7.4 Hz, 1 H), 4.40–4.30 (m, 1 H), 1.46 (s, 9 H), 1.44 (d, *J* = 7.1 Hz, 3 H).

A flame-dried 10-mL flask was charged with a solution of *tert*-butyl (*S*)-(1-((4-((((4-nitrophenoxy)carbonyl)oxy)methyl)phenyl)amino)-1-oxopropan-2-yl)carbamate (20 mg, 0.04 mmol, 1 eq) in DMF (0.5 mL, 0.1 M) was treated with veliparib (10.6 mg, 0.04 mmol, 1 eq) and DIPEA (7.3 µL, 0.04 mmol, 1 eq). The reaction mixture was stirred at room temperature under nitrogen overnight, and partitioned between ethyl acetate and brine. The organic layer washed with brine (4x), dried ($MgSO_4$), filtered, and concentrated in vacuo. The crude residue was purified by chromatography on SiO_2 (1% MeOH in CH_2Cl_2 to 5 % MeOH in CH_2Cl_2) to give **6** (16 mg, 0.03 mmol, 62%) as a white, solid mixture of rotamers: **IR** (ATR, CH_2Cl_2) 3285, 2982, 1672, 1606, 1518, 1413, 1353, 1247, 1164 cm^{-1}; **^{1}H NMR** (500 MHz, DMSO-d_6) δ 12.67 (s, 0.5 H), 12.63 (s, 0. 5H), 9.94 (s, 0.5 H), 9.77 (s, 0.5 H), 9.27 (d, *J* = 4.0 Hz, 1 H), 7.82 (ddd, *J* = 8.8, 7.5, 1.1 Hz, 1 H), 7.69–7.62 (m, 1.5 H), 7.62–7.52 (m, 1.5 H), 7.30 (dd, *J* = 8.0, 5.8 Hz, 2 H), 7.19 (d, *J* = 8.3 Hz, 1 H), 7.08–7.00 (m, 1 H), 6.63 (dd, *J* = 8.5, 3.5 Hz, 1 H), 4.99 (d, *J* = 12.7 Hz, 0.5 H), 4.90 (d, *J* = 12.6 Hz, 0.5 H), 4.85–4.74 (m, 1 H), 4.15–4.03 (m, 1 H), 3.80–3.74 (m, 1 H), 3.63 (dt, *J* = 10.8, 6.3 Hz, 1 H), 2.20 (dq, *J* = 12.8, 6.5, 5.9 Hz, 1 H), 2.12 (dt, *J* = 12.8, 6.6 Hz, 0.5 H), 1.96 (tq, *J* = 13.7, 7.1 Hz, 2 H), 1.87 (s, 1.5 H), 1.84 (t, *J* = 4.2 Hz, 1 H), 1.38 (s, 9 H), 1.32–1.20 (m, 4 H); **^{13}C NMR** (151 MHz, DMSO-d_6) δ 171.9, 166.3, 160.0, 159.8, 155.2, 153.6, 153.2, 140.6, 138.7, 134.8, 131.5, 128.3, 127.5, 122.1, 121.6, 119.1, 118.6, 114.8, 78.0, 65.7, 62.1, 61.6, 50.4, 48.4, 47.6, 43.0, 41.7, 34.2, 31.0, 28.2, 24.8, 24.0, 22.7, 22.6, 22.0, 20.6, 18.0, 14.0; **HRMS** (ESI$^+$) *m/z* calcd for $C_{29}H_{37}N_6O_6$ [M+H]$^+$, 565.2769; found, 565.2757.

Assays

PARP-1 Inhibitory Activity. PARP-1 inhibition was determined using a PARP-1 colorimetric assay Kit from BPS Bioscience (catalog #80580) and the microplate reader BioTek Synergy H1 capable of reading absorbance at 450 nm. A 5 μM solution of each compound in 10% aqueous DMSO was prepared and tested by adding this solution to histone-coated wells containing PARP1 enzyme, biotinylated NAD^+, and activated DNA template as specified in the manufacturer's direction (final DMSO concentration: 1%, final drug concentration = 0.5 μM). The experiments were performed in triplicate. The control was prepared by adding a 10% DMSO solution in water to the mixture instead of a solution of the drug. The blank was prepared in the same manner of the control but without adding the solution of the PARP-1 enzyme. The percentage of PARP-1 inhibition was determined as described: after subtracting the mean absorbance of the blank to each well, the mean absorbance of the wells containing the PARP-1 inhibitor was subtracted to the control absorbance (100 % of PARP-1 activity) as shown in the Equation 1. Then, the percentage of PARP-1 activity left was calculated as shown if Equation 2. To determine the percentage of PARP-1 inhibition, the Equation 3 was used. The standard deviation was calculated using N = 3.

Equation 1 : $\text{Absorbance}_{(\text{CONTROL})} - \text{Absorbance}_{(\text{PARP-1 INHIBITOR})} = \text{Absorbance}_{(\text{N})}$

Equation 2 : $\text{Absorbance}_{(\text{N})} : x = \text{Absorbance}_{(\text{CONTROL})} : 100$

Equation 3 : $100 - x = \%$ of PARP-1 inhibition

Metabolic Studies and Related PARP-1 Inhibitory Activity. The drug metabolism was performed using mixed gender human liver lysosomes (0.25 mL at 2 mg/mL, catalog #H0610.L) and 10x catabolic buffer (catalog #K5200) purchased from Sekisui Xenotech. The human liver lysosomes were diluted before their use 1:10 in water to obtain a concentration of 0.2 mg/mL. 100 μL of this diluted solution was withdrawn and added to a 0.5-mL Eppendorf tube containing 78 μL of water and 20 μL of 10x catabolic buffer. To this mixture was added 2 μL of a 0.5 mM solution of the drug in 10% aq. DMSO to obtain 200 μL of a 5 μM final drug concentration. The mixture was vortexed and incubated at 37 °C with gentle shaking. As a control, 2 μL of the PARP-1 inhibitor solution were replaced with the same volume of a 10% DMSO solution for the preparation of the mixture with human liver lysosomes. After 30 min, 2 h, and 24 h, an aliquot of 50 μL was withdrawn from this mixture and poured into a 0.5-mL Eppendorf tube containing 100 μL of cold acetonitrile. The mixture was centrifuged for 15 min at 4400 rpm and the supernatant removed via syringe and transferred to a new 0.5-mL Eppendorf tube. The remaining pellet was rinsed with additional 100 μL of cold acetonitrile and the supernatant was transferred to the Eppendorf tube and mixed with the previous one. This process was repeated another time (three times total) before the

solvents were evaporated under high vacuum in a desiccator containing drierite®. The residue was redissolved in 50 μL of 10% aq. DMSO (final drug concentration if no metabolism occurred: 5 μM) and used to evaluate PARP-1 inhibition as described above. The experiments were performed in triplicate.

Copies of NMR Spectra

^{1}H NMR (300 MHz, Chloroform-d)

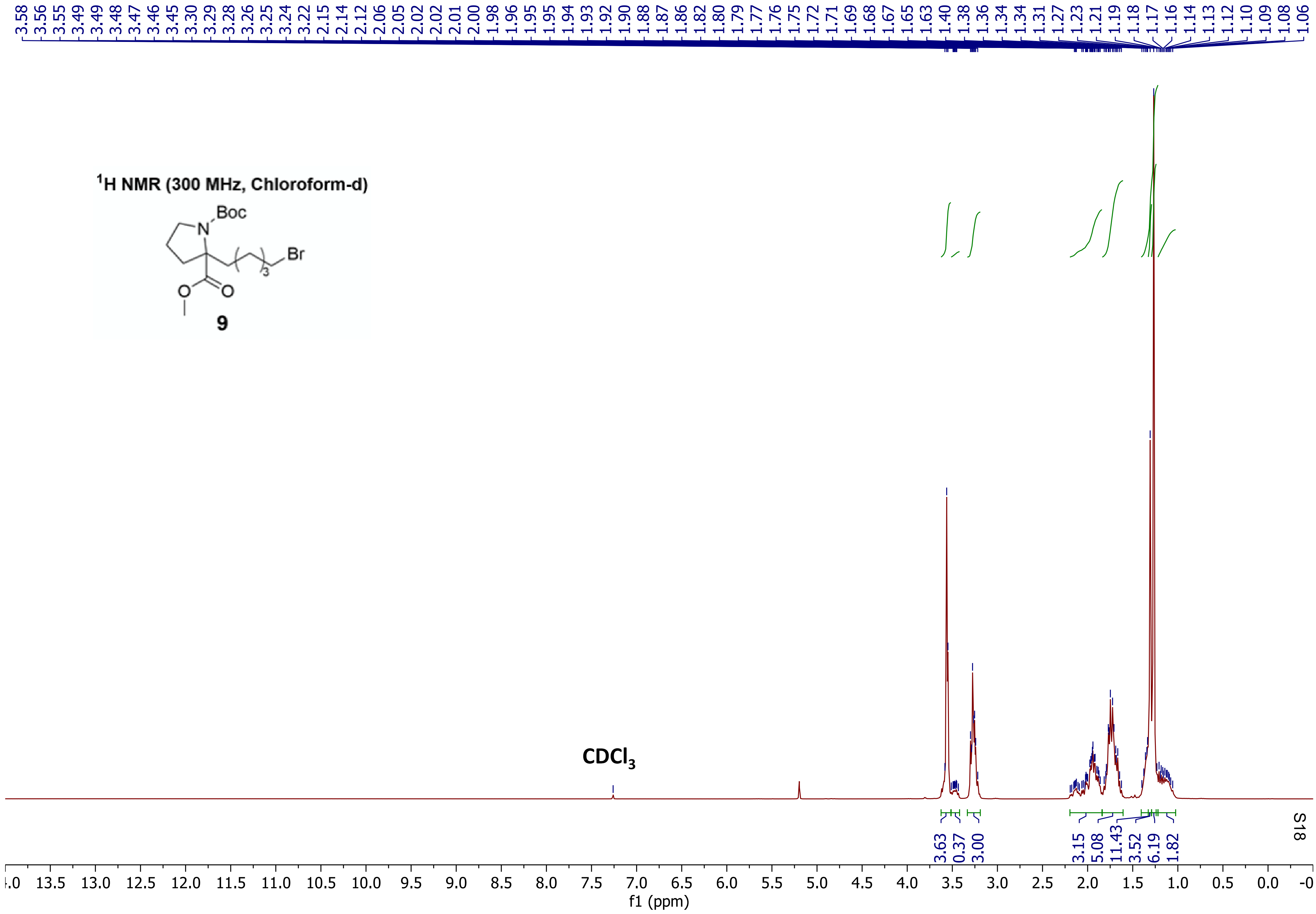

^{13}C NMR (76 MHz, Chloroform-d)

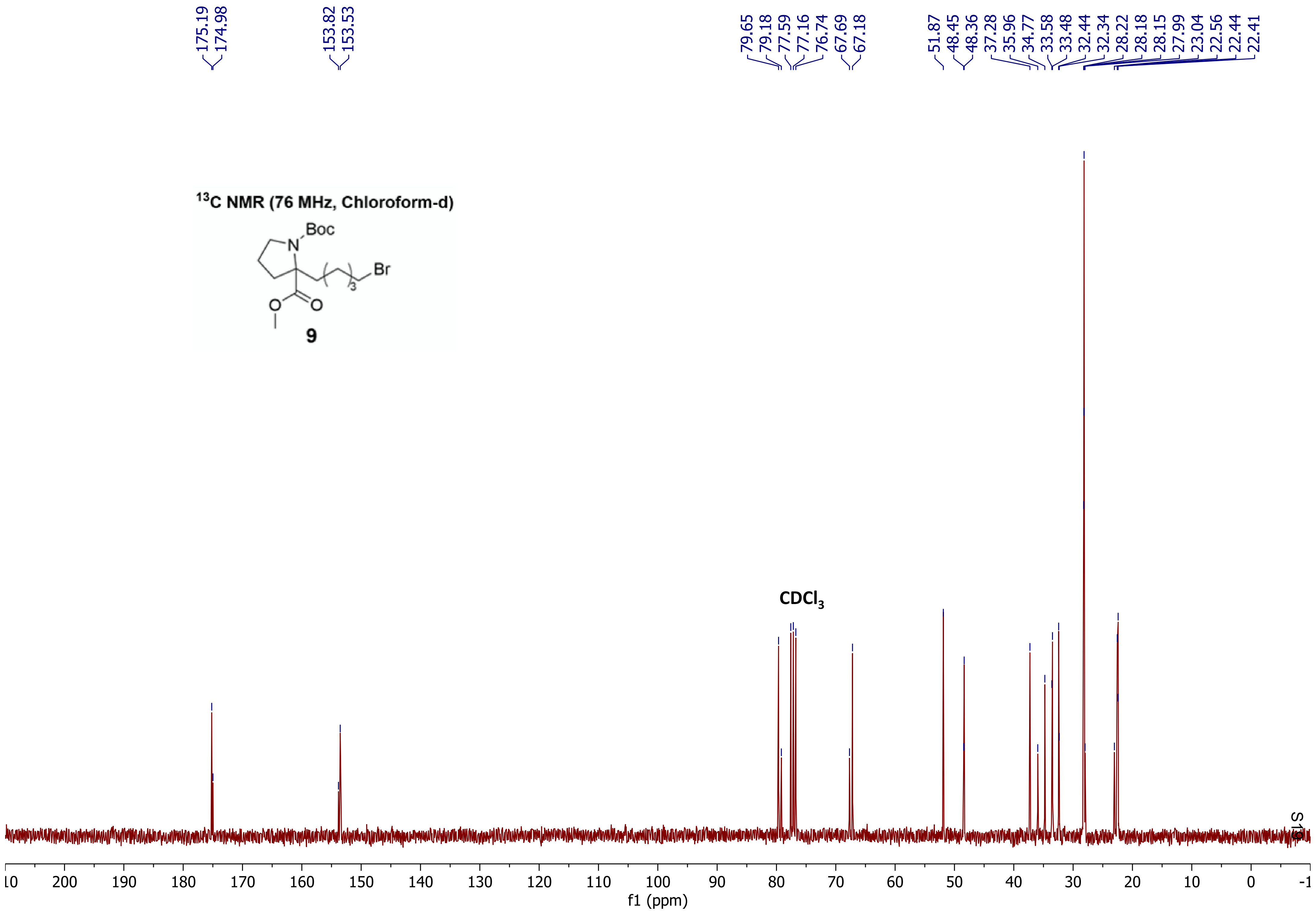

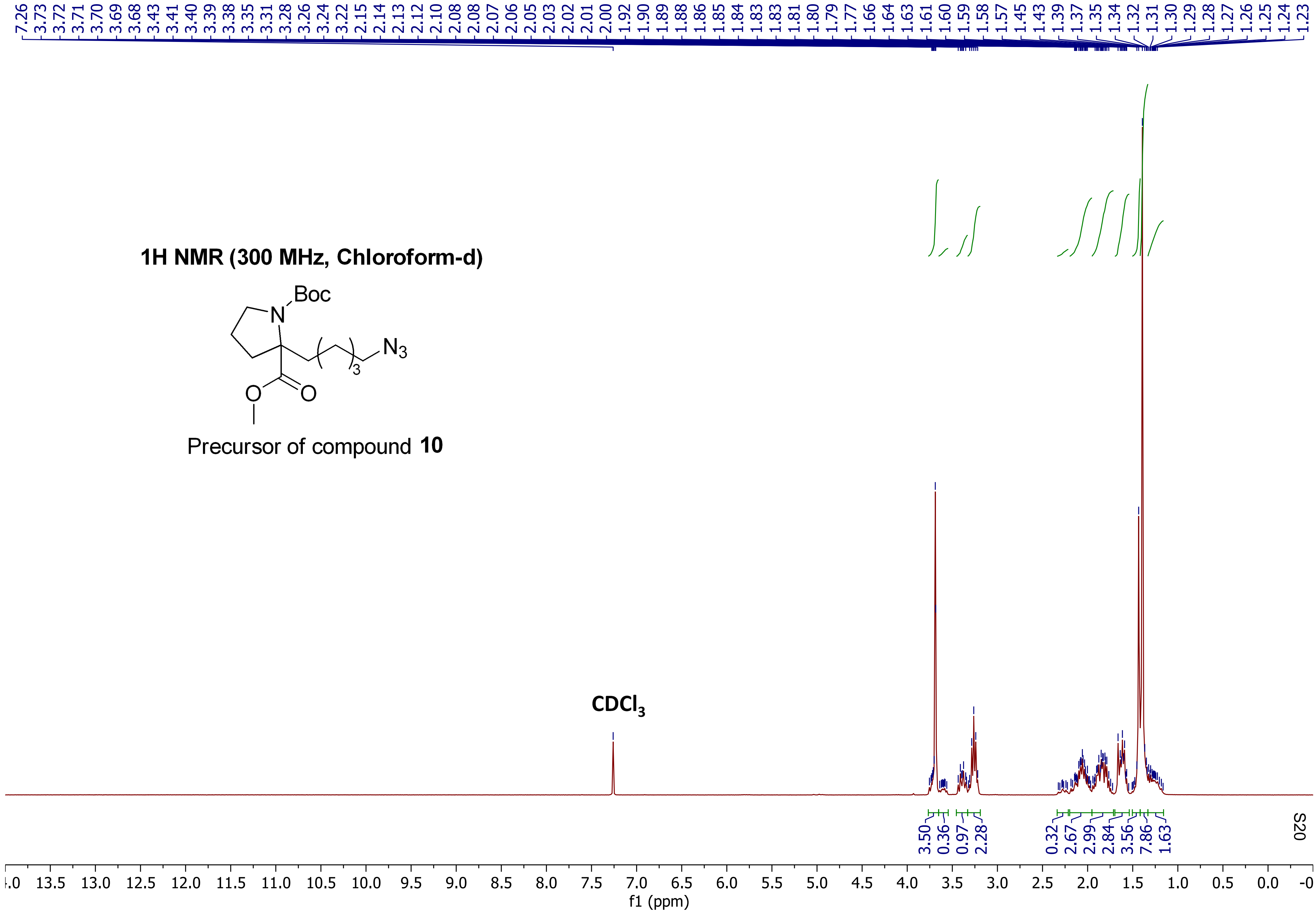
7.26
3.73
3.72
3.71
3.70
3.69
3.68
3.43
3.41
3.40
3.39
3.38
3.35
3.31
3.28
3.26
3.24
3.22
2.15
2.14
2.13
2.12
2.10
2.08
2.08
2.07
2.06
2.05
2.03
2.02
2.01
2.00
1.92
1.90
1.89
1.88
1.86
1.85
1.84
1.83
1.83
1.81
1.80
1.79
1.77
1.66
1.64
1.63
1.61
1.60
1.59
1.58
1.57
1.45
1.43
1.39
1.37
1.35
1.34
1.32
1.31
1.30
1.29
1.28
1.27
1.26
1.25
1.24
1.23
1H NMR (300 MHz, Chloroform-d)
Boc
N
N3
3
O
O
Precursor of compound 10
CDCl3
3.50
0.36
0.97
2.28
0.32
2.67
2.99
2.84
3.56
7.86
1.63
13.5 13.0 12.5 12.0 11.5 11.0 10.5 10.0 9.5 9.0 8.5 8.0 7.5 7.0 6.5 6.0 5.5 5.0 4.5 4.0 3.5 3.0 2.5 2.0 1.5 1.0 0.5 0.0
f1 (ppm)

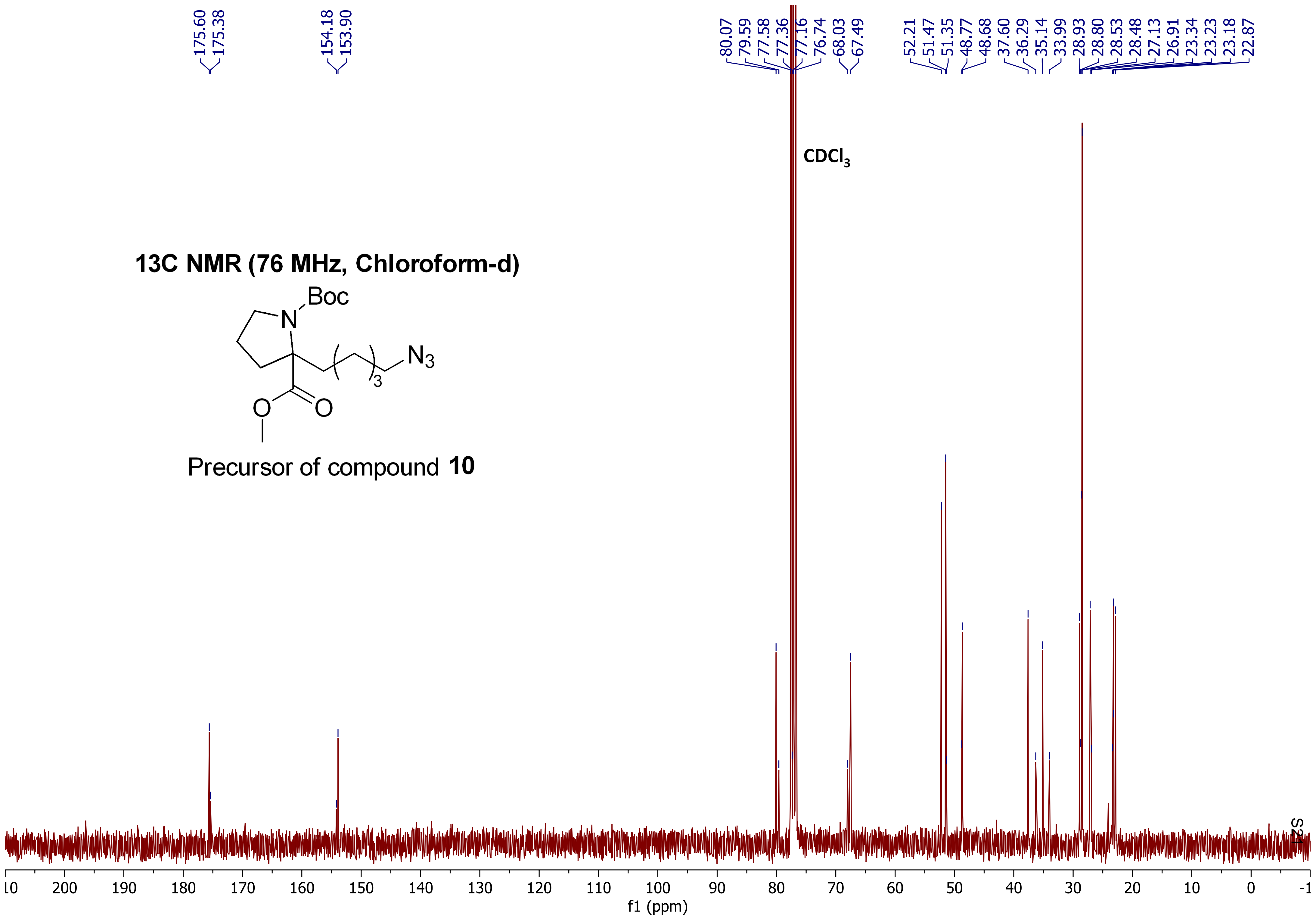

175.60
175.38
154.18
153.90
80.07
79.59
77.58
77.36
77.16
76.74
68.03
67.49
52.21
51.47
51.35
48.77
48.68
37.60
36.29
35.14
33.99
28.93
28.80
28.53
28.48
27.13
26.91
23.34
23.23
23.18
22.87
CDCl3
13C NMR (76 MHz, Chloroform-d)
Boc
N
N3
3
O
O
Precursor of compound 10
f1 (ppm)

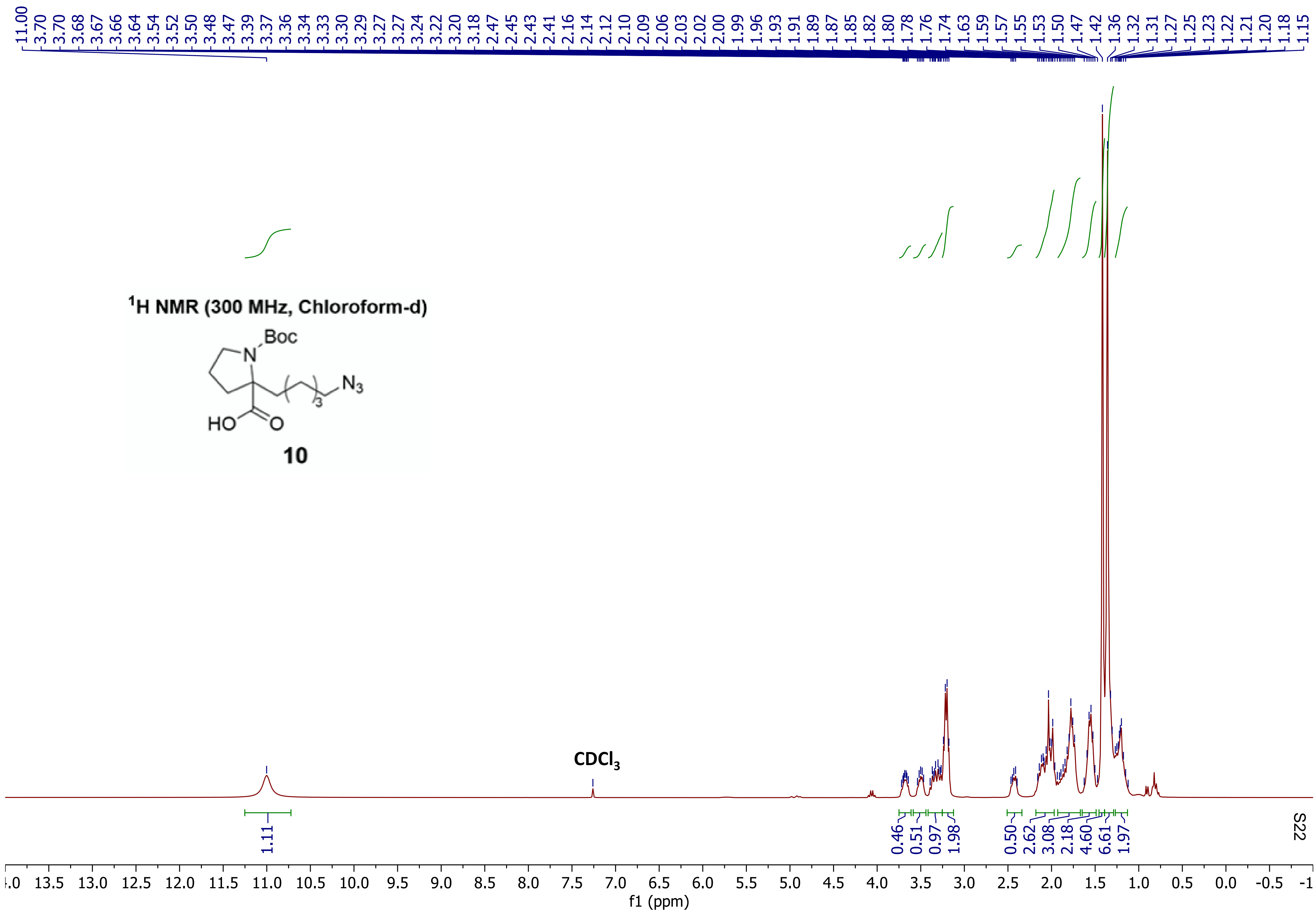
11.00 3.70 3.70 3.68 3.67 3.66 3.64 3.54 3.52 3.50 3.48 3.47 3.39 3.37 3.36 3.34 3.33 3.30 3.29 3.27 3.27 3.24 3.22 3.20 3.18 2.47 2.45 2.43 2.41 2.16 2.14 2.12 2.10 2.09 2.06 2.03 2.02 2.00 1.99 1.96 1.93 1.91 1.89 1.87 1.85 1.82 1.80 1.78 1.76 1.74 1.63 1.59 1.57 1.55 1.53 1.50 1.47 1.42 1.36 1.32 1.31 1.27 1.25 1.23 1.22 1.21 1.20 1.18 1.15
1H NMR (300 MHz, Chloroform-d)
Boc
N
N3
3
HO
O
10
CDCl3
1.11
0.46 0.51 0.97 1.98
0.50 2.62 3.08 2.18 4.60 6.61 1.97
14.0 13.5 13.0 12.5 12.0 11.5 11.0 10.5 10.0 9.5 9.0 8.5 8.0 7.5 7.0 6.5 6.0 5.5 5.0 4.5 4.0 3.5 3.0 2.5 2.0 1.5 1.0 0.5 0.0 -0.5 -1
f1 (ppm)

^{13}C NMR (76 MHz, Chloroform-d)

10

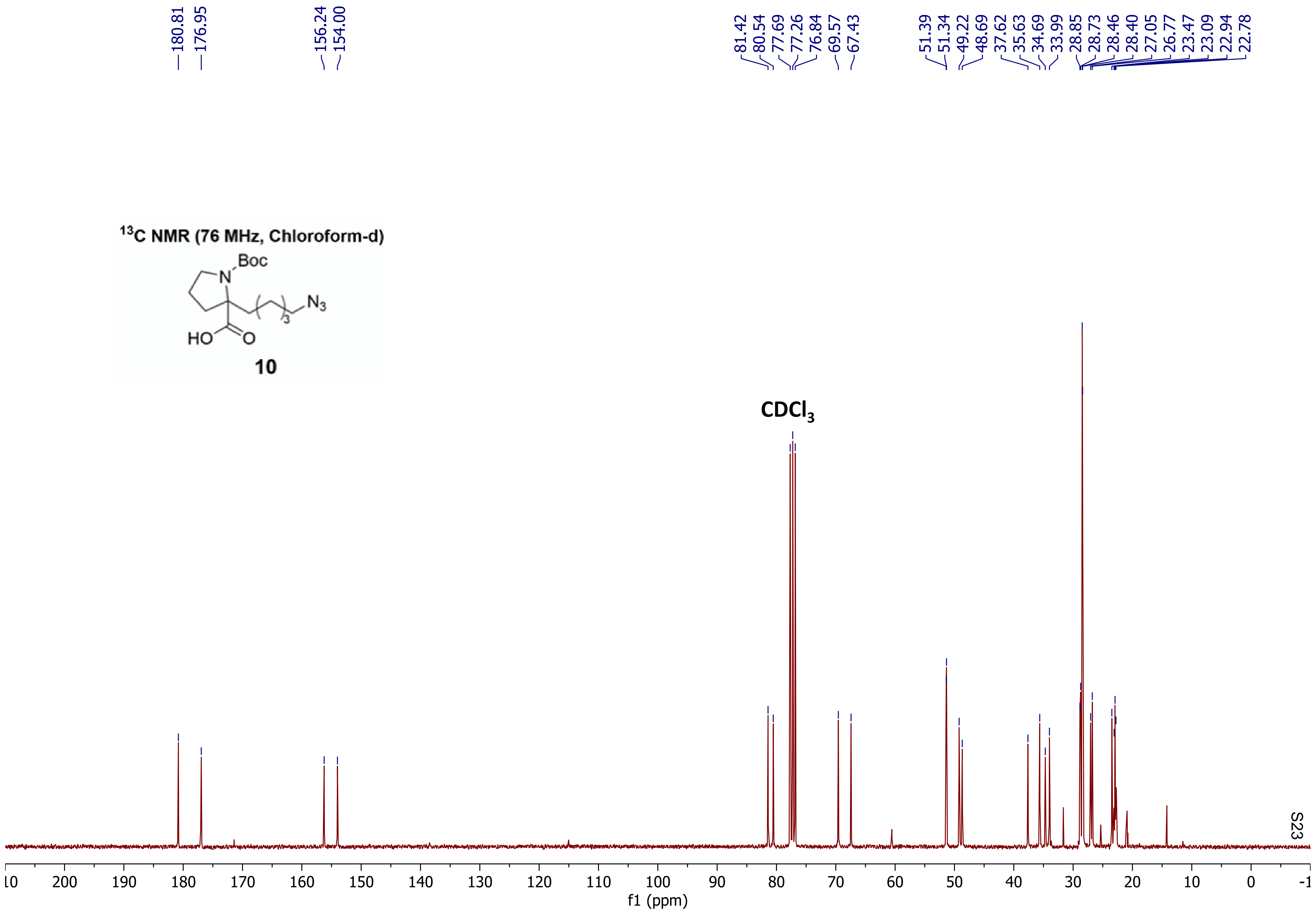

S23

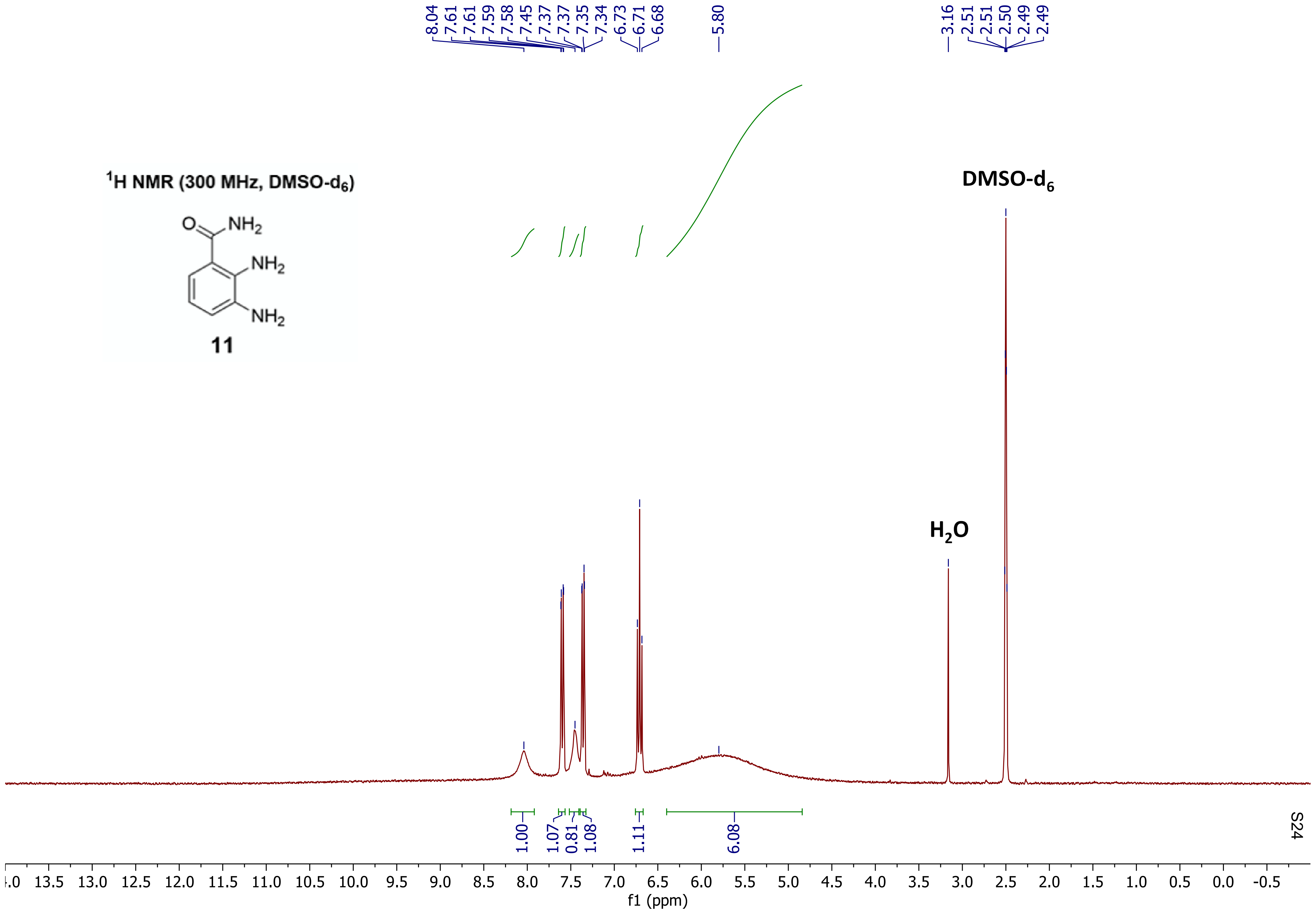

8.04
7.61
7.61
7.59
7.58
7.45
7.37
7.37
7.35
7.34
6.73
6.71
6.68
5.80
3.16
2.51
2.51
2.50
2.49
2.49
¹H NMR (300 MHz, DMSO-d6)
NH2
O
NH2
NH2
11
DMSO-d6
H2O
1.00
1.07
0.81
1.08
1.11
6.08
f1 (ppm)

¹H NMR (300 MHz, Chloroform-d)

12

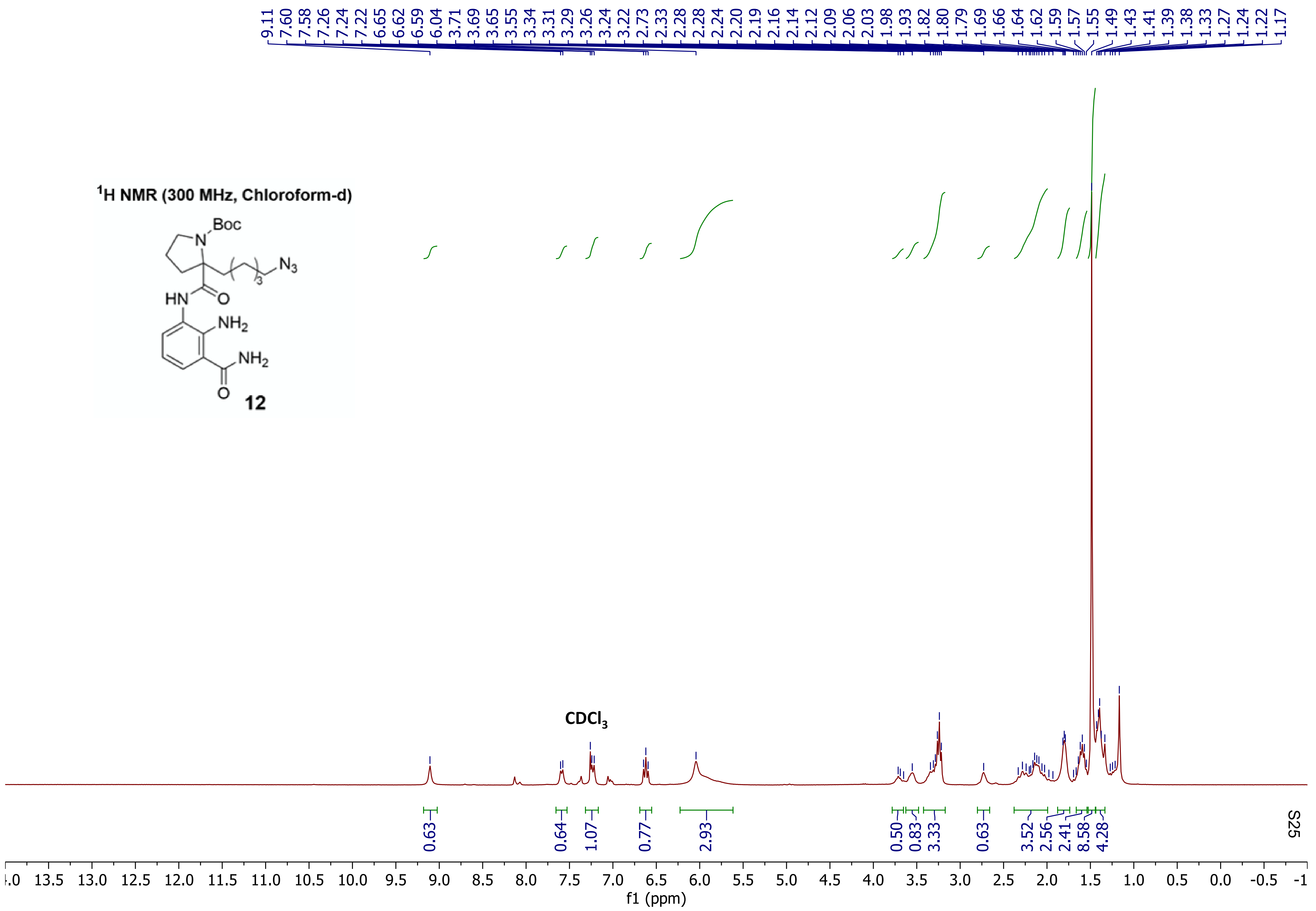

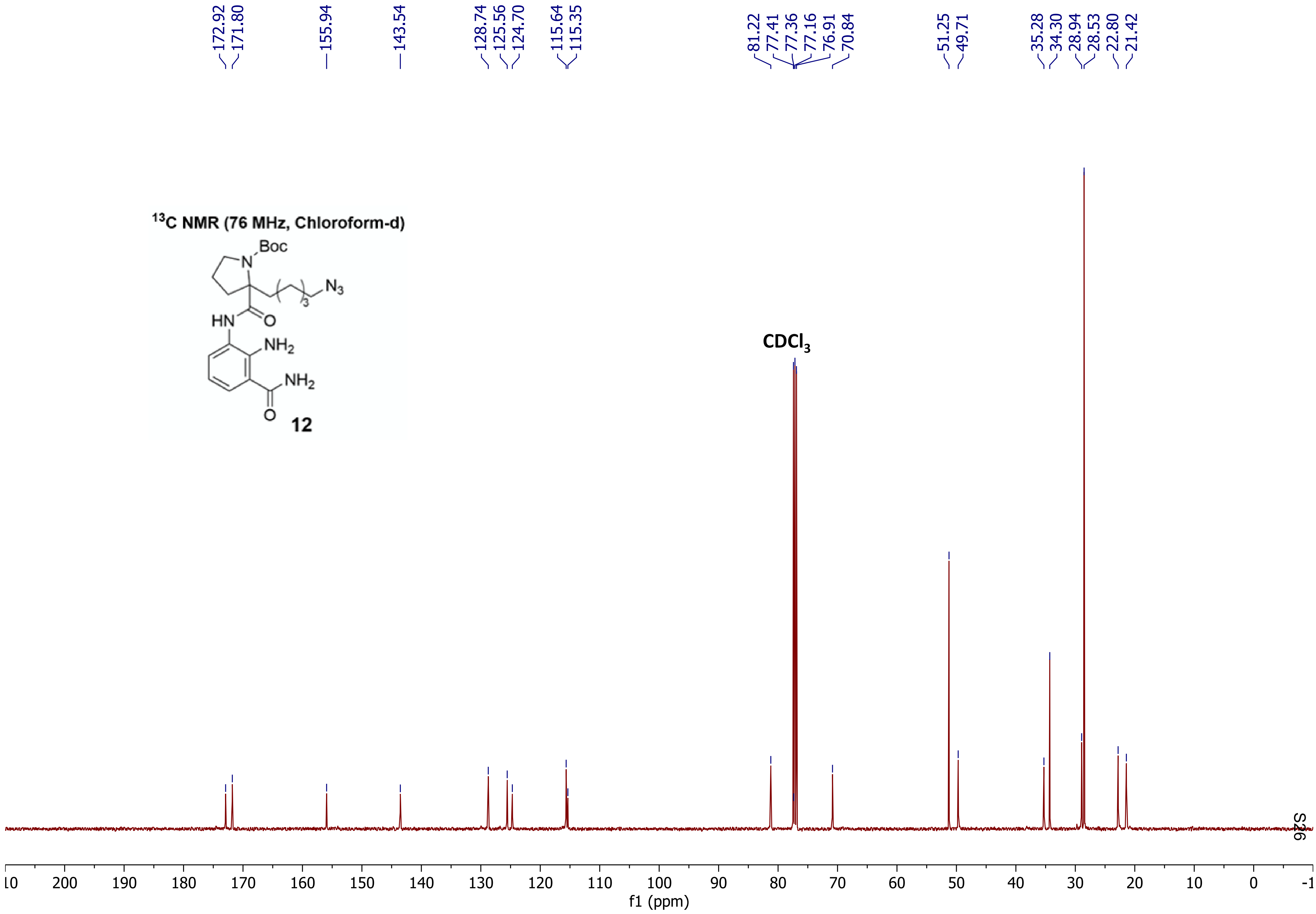
172.92
171.80
155.94
143.54
128.74
125.56
124.70
115.64
115.35
81.22
77.41
77.36
77.16
76.91
70.84
51.25
49.71
35.28
34.30
28.94
28.53
22.80
21.42
^{13}C NMR (76 MHz, Chloroform-d)
Boc
N
N_3
3
HN
O
NH_2
NH_2
O
12
$CDCl_3$
f1 (ppm)

^{1}H NMR (500 MHz, Chloroform-d)

13

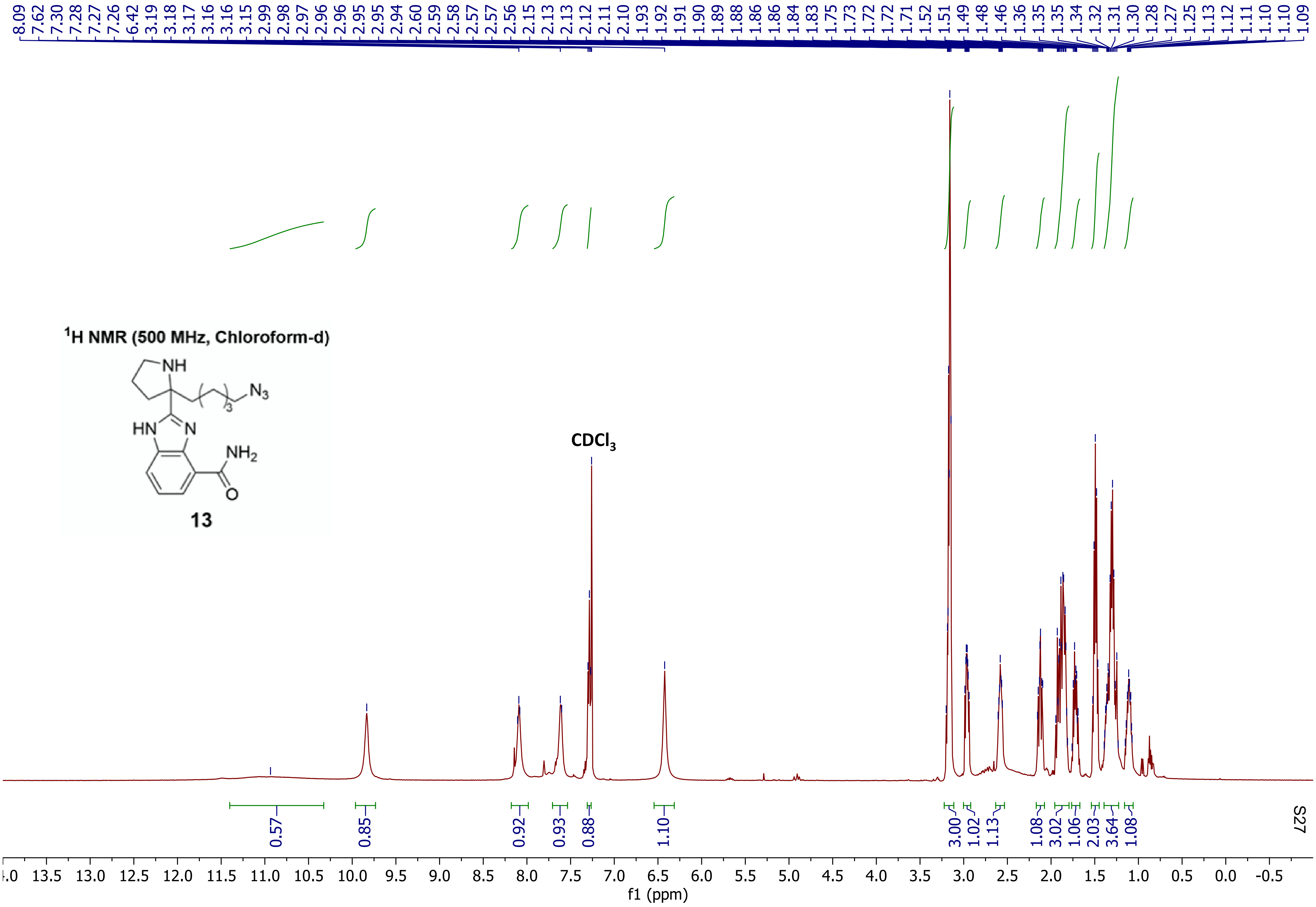

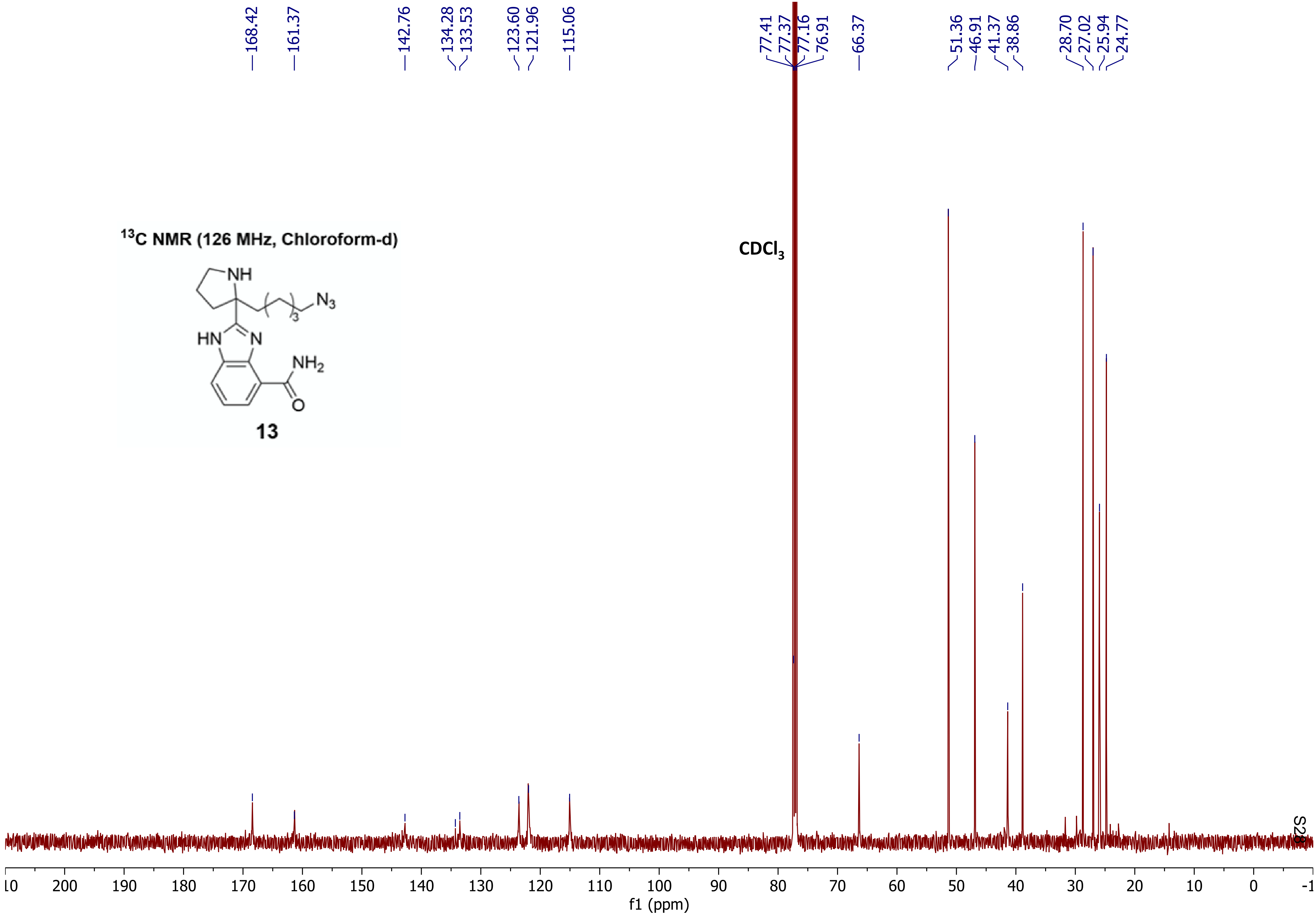
168.42
161.37
142.76
134.28
133.53
123.60
121.96
115.06
77.41
77.37
77.16
76.91
66.37
51.36
46.91
41.37
38.86
28.70
27.02
25.94
24.77
^{13}C NMR (126 MHz, Chloroform-d)
NH
N_3
3
HN
N
NH_2
O
13
$CDCl_3$
210
200
190
180
170
160
150
140
130
120
110
100
90
80
70
60
50
40
30
20
10
0
-1
f1 (ppm)

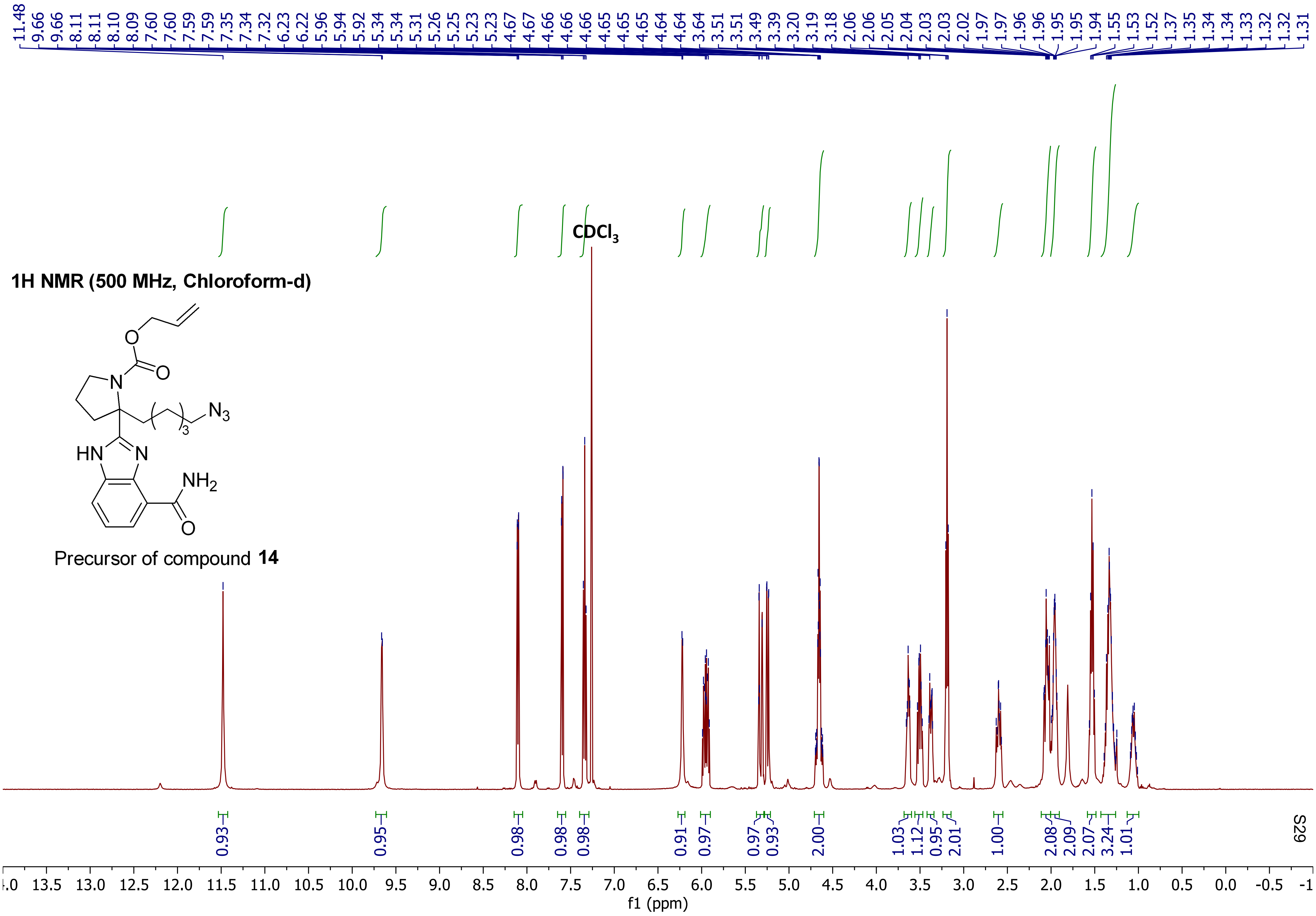

Precursor of compound **14**

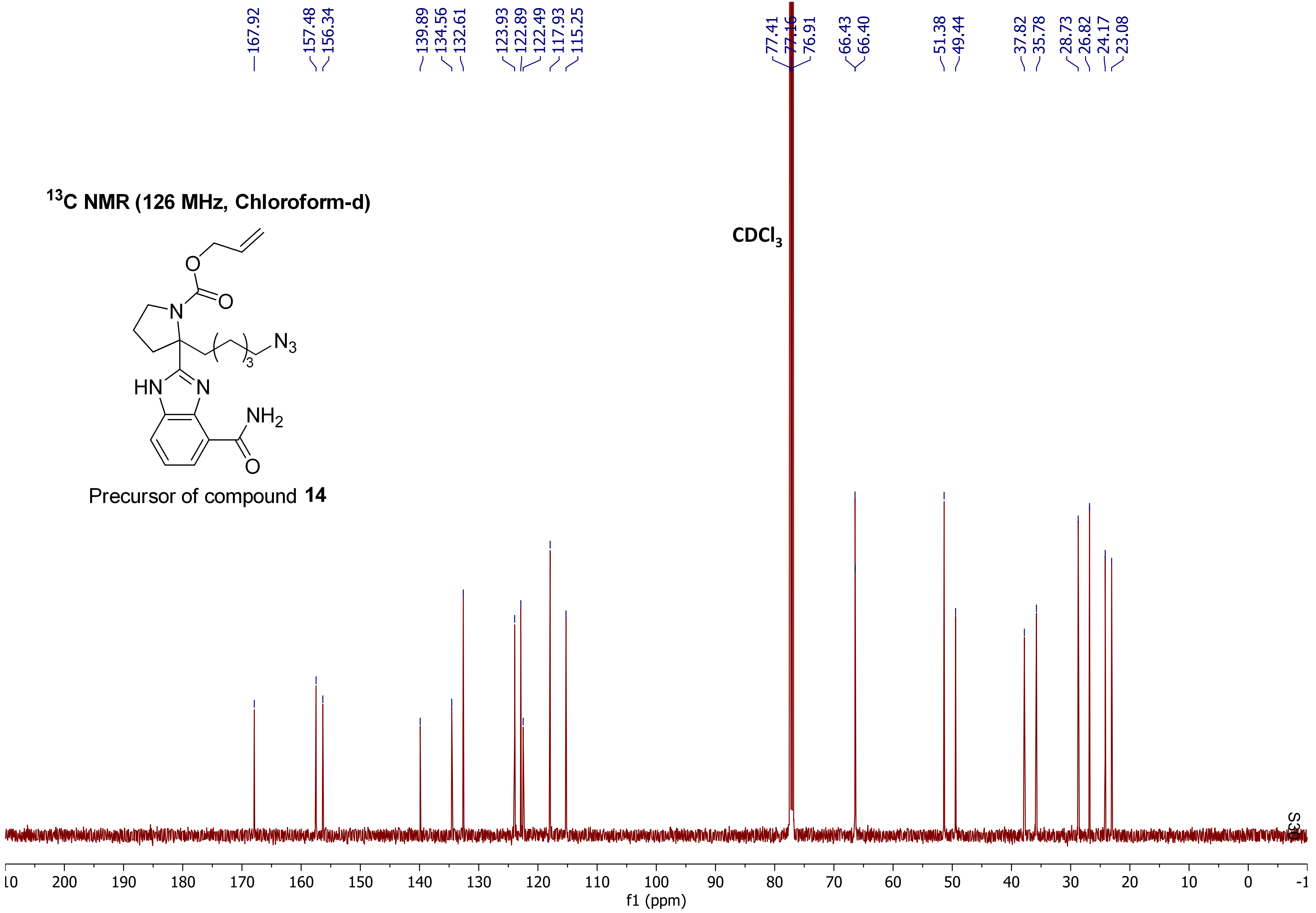

Precursor of compound **14**

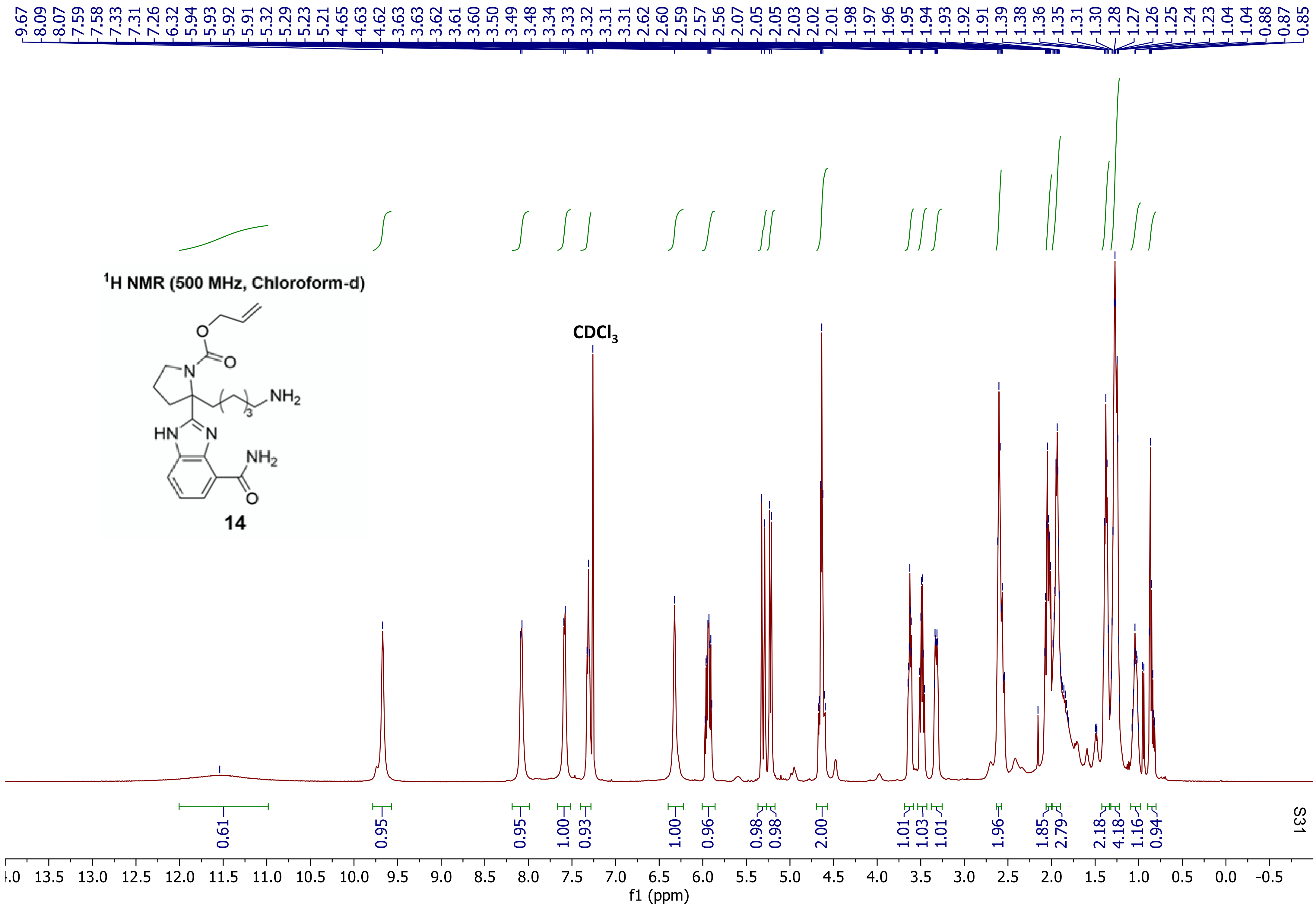

^{1}H NMR (500 MHz, Chloroform-d)
14
CDCl$_3$
f1 (ppm)

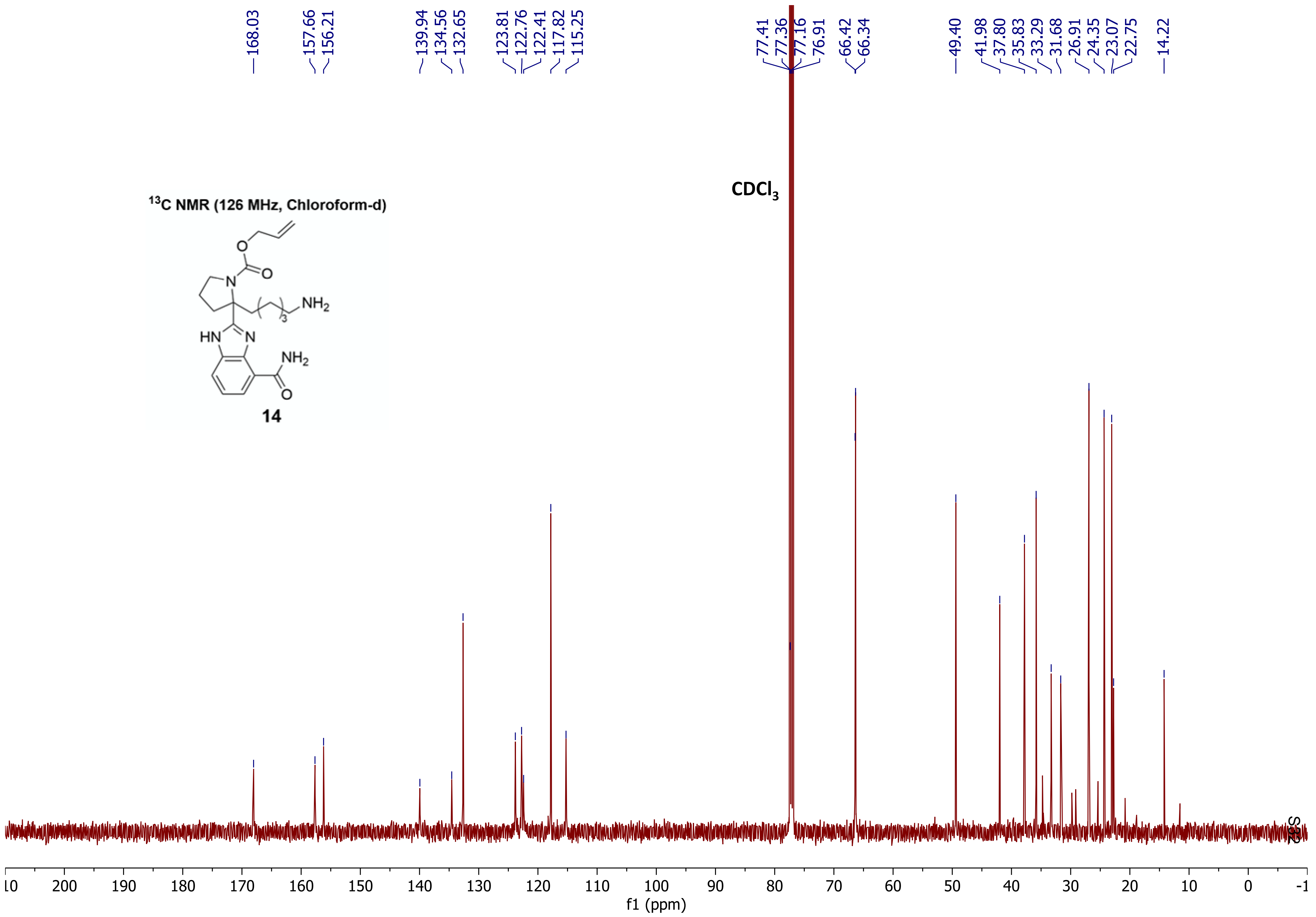

13C NMR (126 MHz, Chloroform-d)
14
CDCl3
168.03
157.66
156.21
139.94
134.56
132.65
123.81
122.76
122.41
117.82
115.25
77.41
77.36
77.16
76.91
66.42
66.34
49.40
41.98
37.80
35.83
33.29
31.68
26.91
24.35
23.07
22.75
14.22
f1 (ppm)

^{1}H NMR (500 MHz, Chloroform-d)

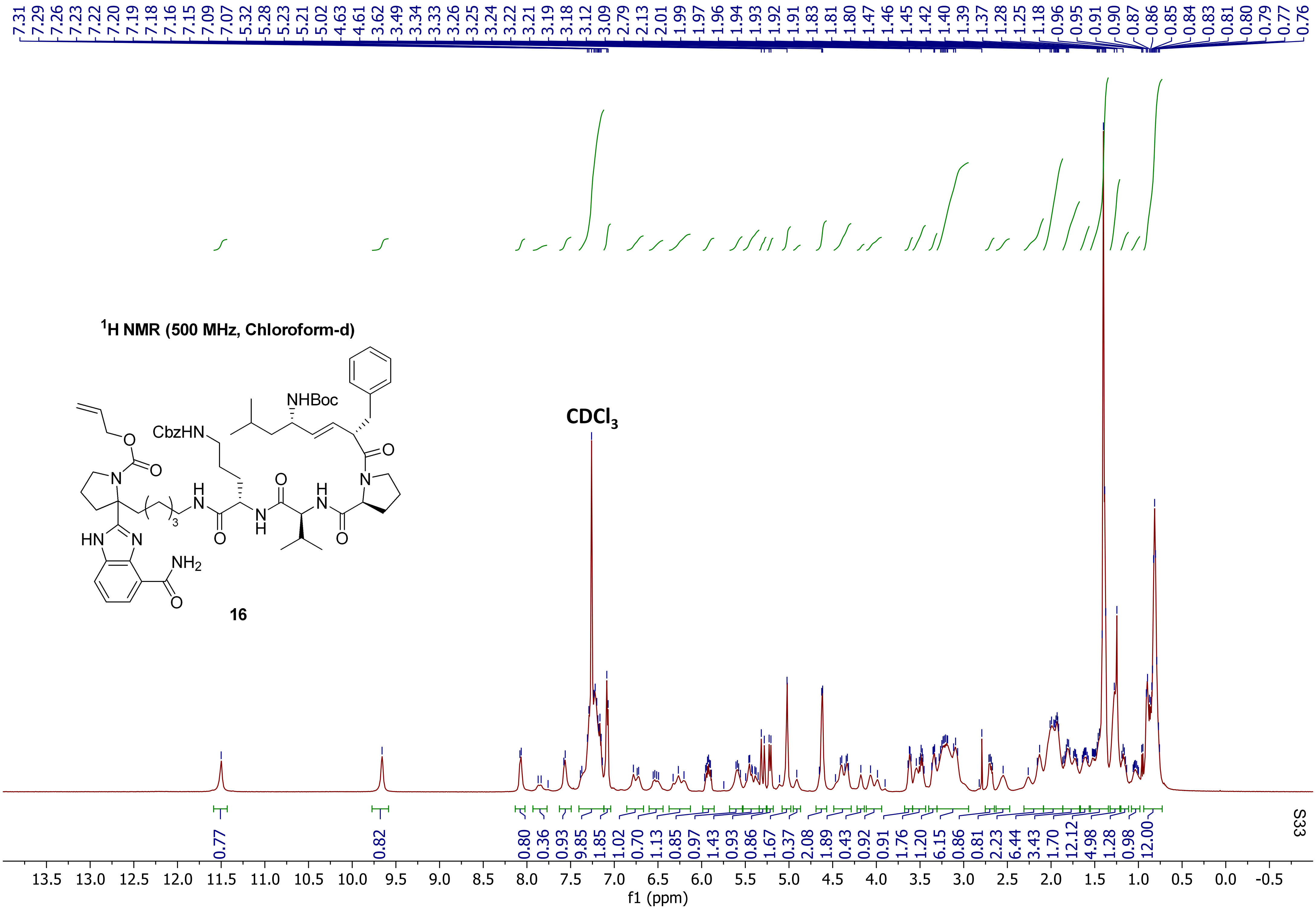

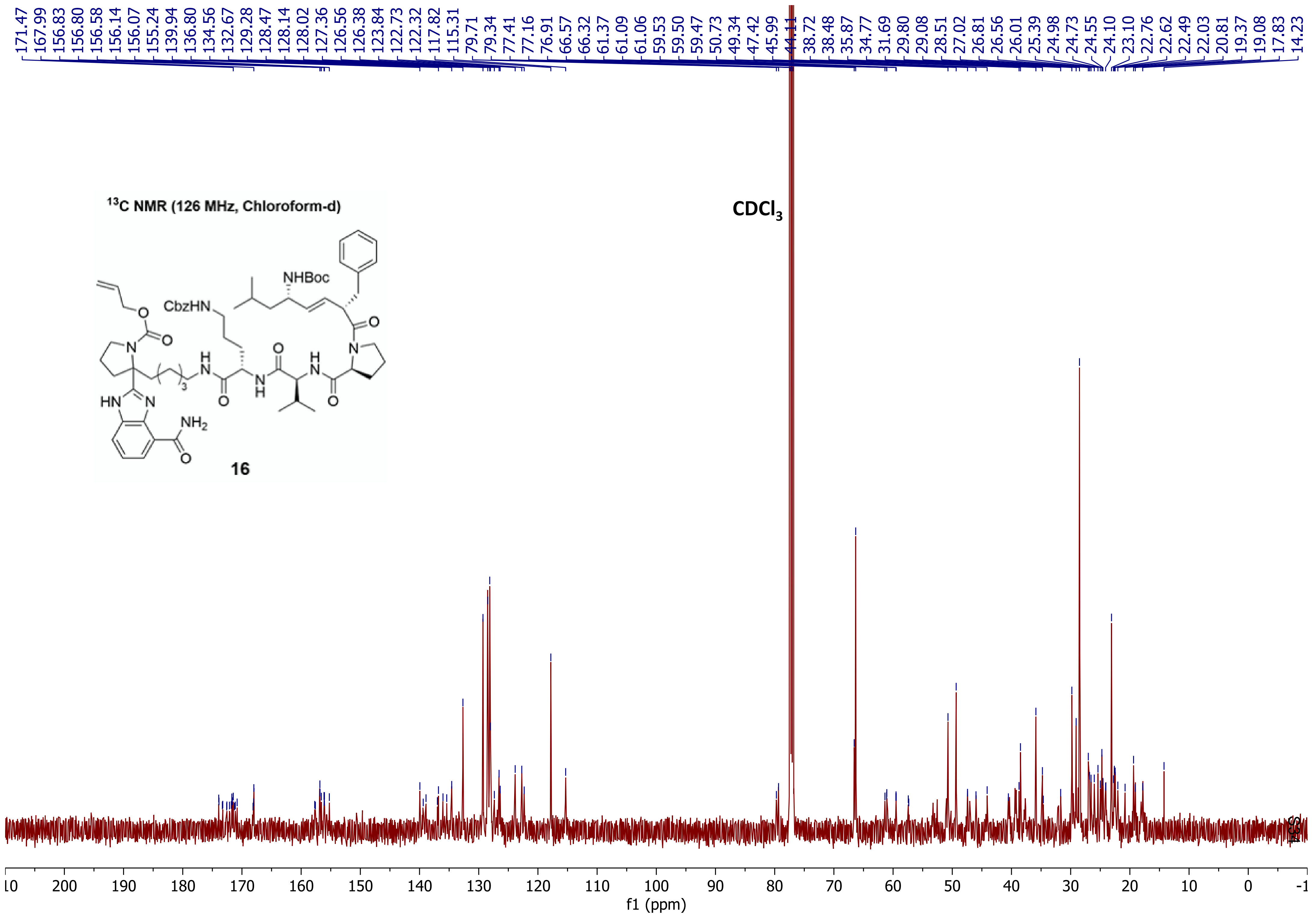
171.47
167.99
156.83
156.80
156.58
156.14
156.07
155.24
139.94
136.80
134.56
132.67
129.28
128.47
128.14
128.02
127.36
126.56
126.38
123.84
122.73
122.32
117.82
115.31
79.71
79.34
77.41
77.16
76.91
66.57
66.32
61.37
61.09
61.06
59.53
59.50
59.47
50.73
49.34
47.42
45.99
44.11
38.72
38.48
35.87
34.77
31.69
29.80
29.08
28.51
27.02
26.81
26.56
26.01
25.39
24.98
24.73
24.55
24.10
23.10
22.76
22.62
22.49
22.03
20.81
19.37
19.08
17.83
14.23
^{13}C NMR (126 MHz, Chloroform-d)
CbzHN
NHBoc
NH$_2$
16
CDCl$_3$
f1 (ppm)

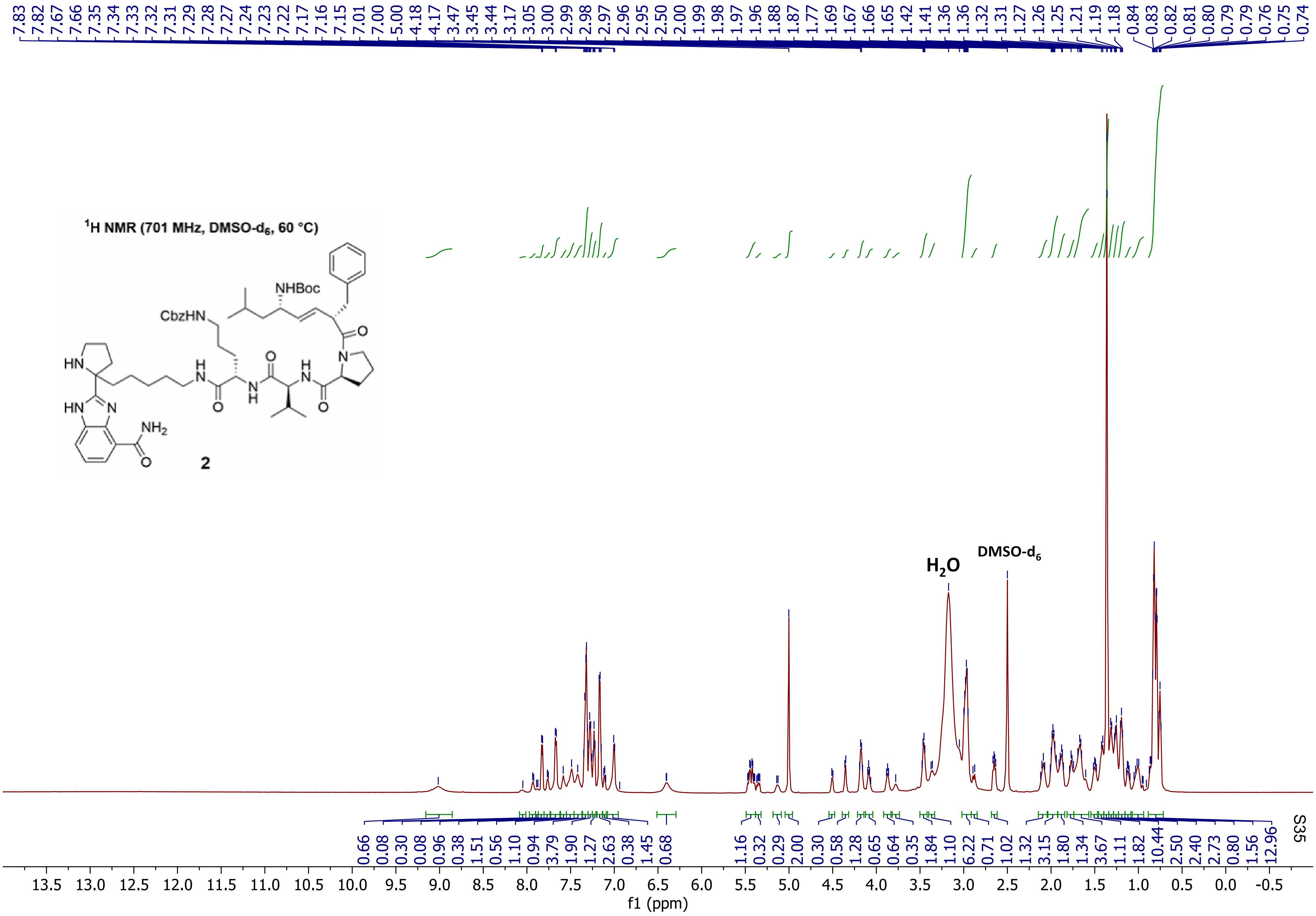
¹H NMR (701 MHz, DMSO-d6, 60 °C)
2
CbzHN
NHBoc
HN
NH2
H2O
DMSO-d6
f1 (ppm)
7.83 7.82 7.67 7.66 7.35 7.34 7.33 7.32 7.31 7.29 7.28 7.27 7.24 7.23 7.22 7.17 7.16 7.15 7.01 7.00 5.00 4.18 4.17 3.47 3.45 3.44 3.17 3.05 3.00 2.99 2.98 2.97 2.96 2.95 2.50 2.00 1.99 1.98 1.97 1.96 1.88 1.87 1.77 1.69 1.67 1.66 1.65 1.42 1.41 1.36 1.36 1.32 1.31 1.27 1.26 1.25 1.21 1.19 1.18 0.84 0.83 0.82 0.81 0.80 0.79 0.79 0.76 0.75 0.74
0.66 0.08 0.30 0.08 0.96 0.38 1.51 0.56 1.10 0.94 3.79 1.90 1.27 2.63 0.38 1.45 0.68 1.16 0.32 0.29 2.00 0.30 0.58 1.28 0.65 0.64 0.35 1.84 1.10 6.22 0.71 1.02 1.32 3.15 1.80 1.34 3.67 1.11 1.82 10.44 2.50 2.40 2.73 0.80 1.56 12.96

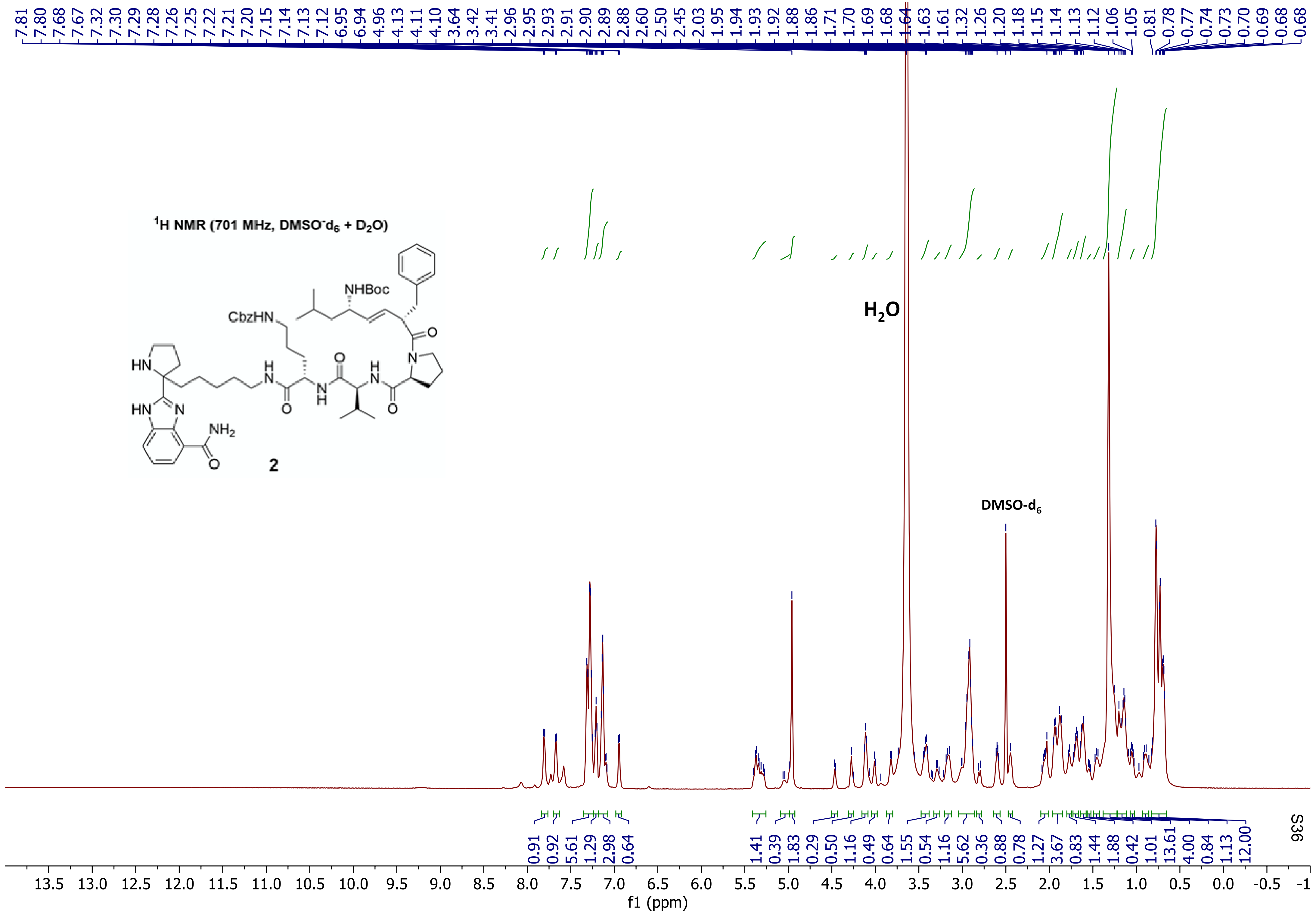
^1H NMR (701 MHz, DMSO-d_6 + D_2O)
2
H_2O
DMSO-d_6
f1 (ppm)

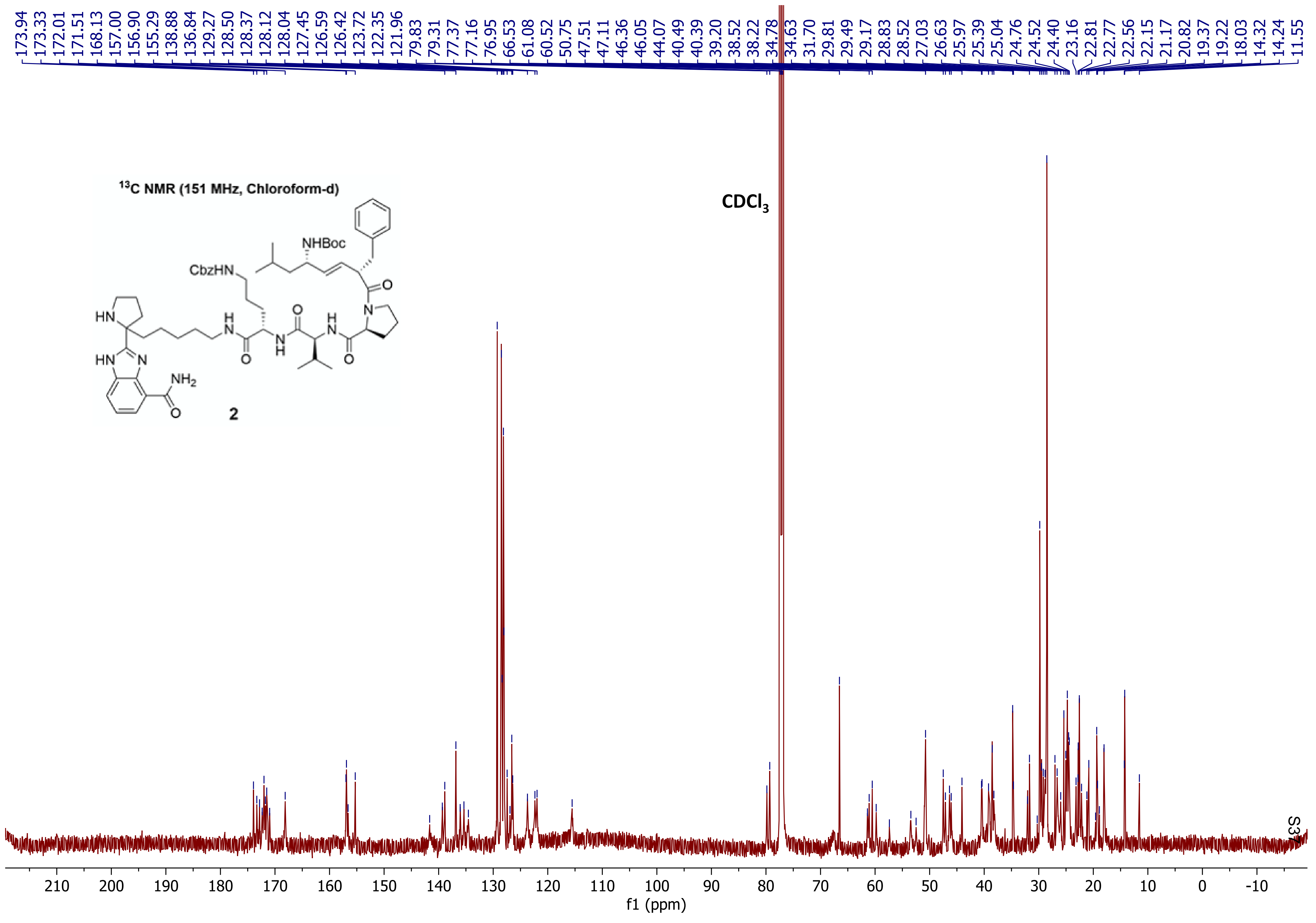
173.94
173.33
172.01
171.51
168.13
157.00
156.90
155.29
138.88
136.84
129.27
128.50
128.37
128.12
128.04
127.45
126.59
126.42
123.72
122.35
121.96
79.83
79.31
77.37
77.16
76.95
66.53
61.08
60.52
50.75
47.51
47.11
46.36
46.05
44.07
40.49
40.39
39.20
38.52
38.22
34.78
34.63
31.70
29.81
29.49
29.17
28.83
28.52
27.03
26.63
25.97
25.39
25.04
24.76
24.52
24.40
23.16
22.81
22.77
22.56
22.15
21.17
20.82
19.37
19.22
18.03
14.32
14.24
11.55
^{13}C NMR (151 MHz, Chloroform-d)
NHBoc
CbzHN
HN
HN
N
NH$_2$
2
CDCl$_3$
f1 (ppm)

S37

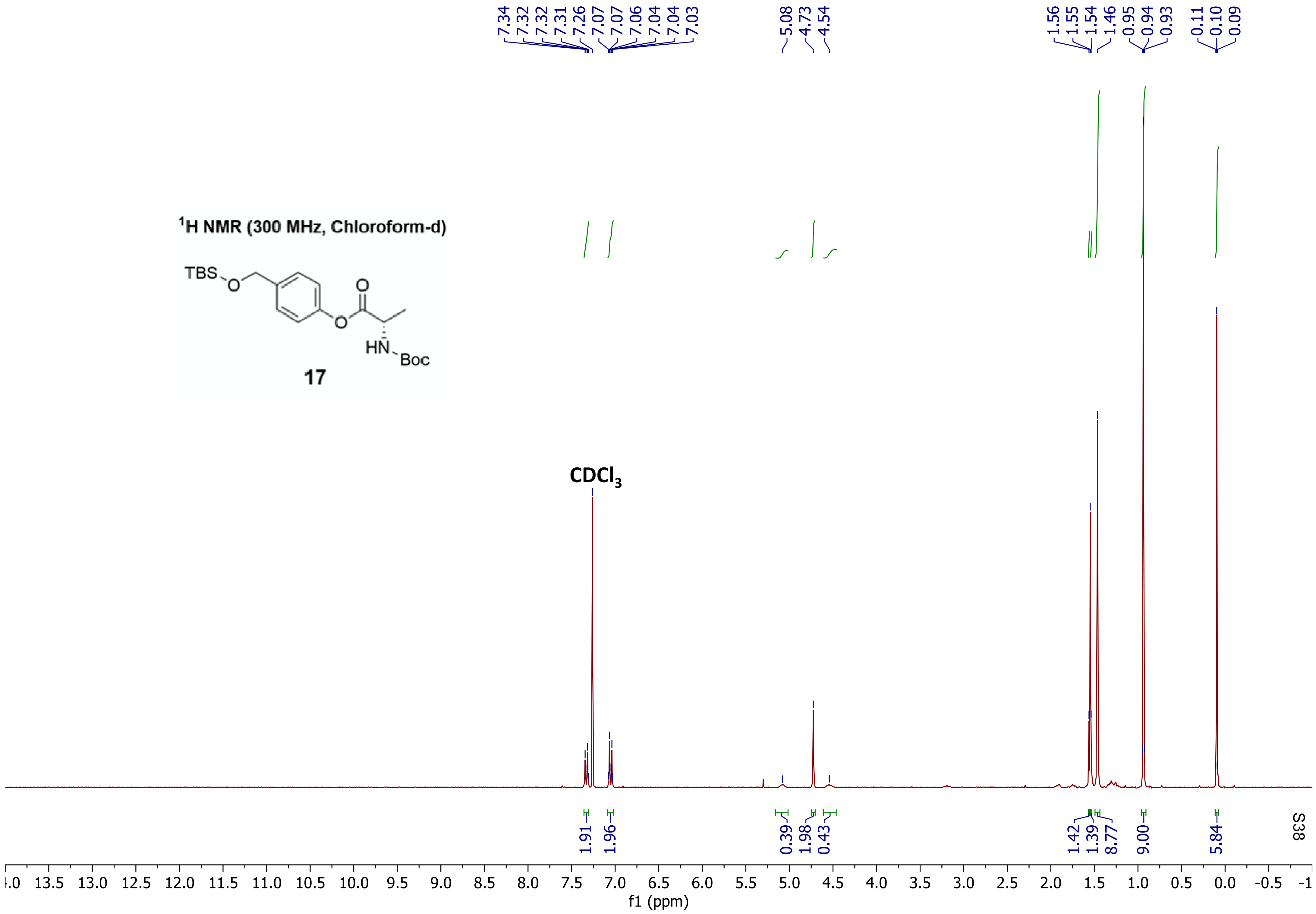

1H NMR (300 MHz, Chloroform-d)
TBS
O
O
O
HN
Boc
17
CDCl3
7.34
7.32
7.32
7.31
7.26
7.07
7.07
7.06
7.04
7.04
7.03
5.08
4.73
4.54
1.56
1.55
1.54
1.46
0.95
0.94
0.93
0.11
0.10
0.09
1.91
1.96
0.39
1.98
0.43
1.42
1.39
8.77
9.00
5.84
f1 (ppm)

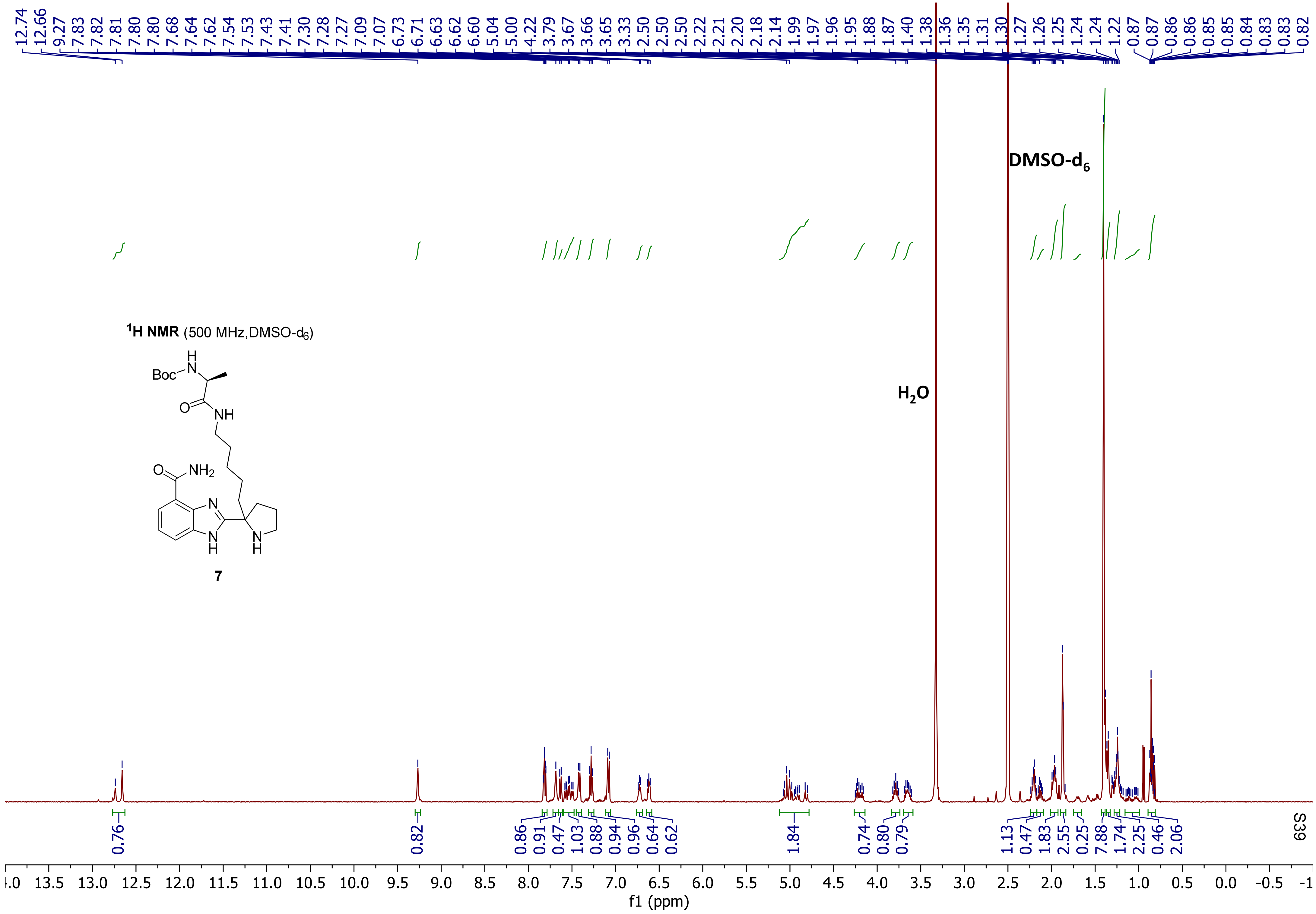
12.74
12.66
9.27
7.83
7.82
7.81
7.80
7.80
7.68
7.64
7.62
7.54
7.53
7.43
7.41
7.30
7.28
7.27
7.09
7.07
6.73
6.71
6.63
6.62
6.60
5.04
5.00
4.22
3.79
3.67
3.66
3.65
3.33
2.50
2.50
2.50
2.22
2.21
2.20
2.18
2.14
1.99
1.97
1.96
1.95
1.88
1.87
1.40
1.38
1.36
1.35
1.31
1.30
1.27
1.26
1.25
1.24
1.24
1.22
0.87
0.87
0.86
0.86
0.85
0.85
0.84
0.83
0.83
0.82
DMSO-d6
H2O
1H NMR (500 MHz,DMSO-d6)
Boc
H
N
O
NH
O
NH2
N
N
H
N
H
7
0.76
0.82
0.86
0.91
0.47
1.03
0.88
0.94
0.96
0.64
0.62
1.84
0.74
0.80
0.79
1.13
0.47
1.83
2.55
0.25
7.88
1.74
2.25
0.46
2.06
14.0 13.5 13.0 12.5 12.0 11.5 11.0 10.5 10.0 9.5 9.0 8.5 8.0 7.5 7.0 6.5 6.0 5.5 5.0 4.5 4.0 3.5 3.0 2.5 2.0 1.5 1.0 0.5 0.0 -0.5 -1
f1 (ppm)

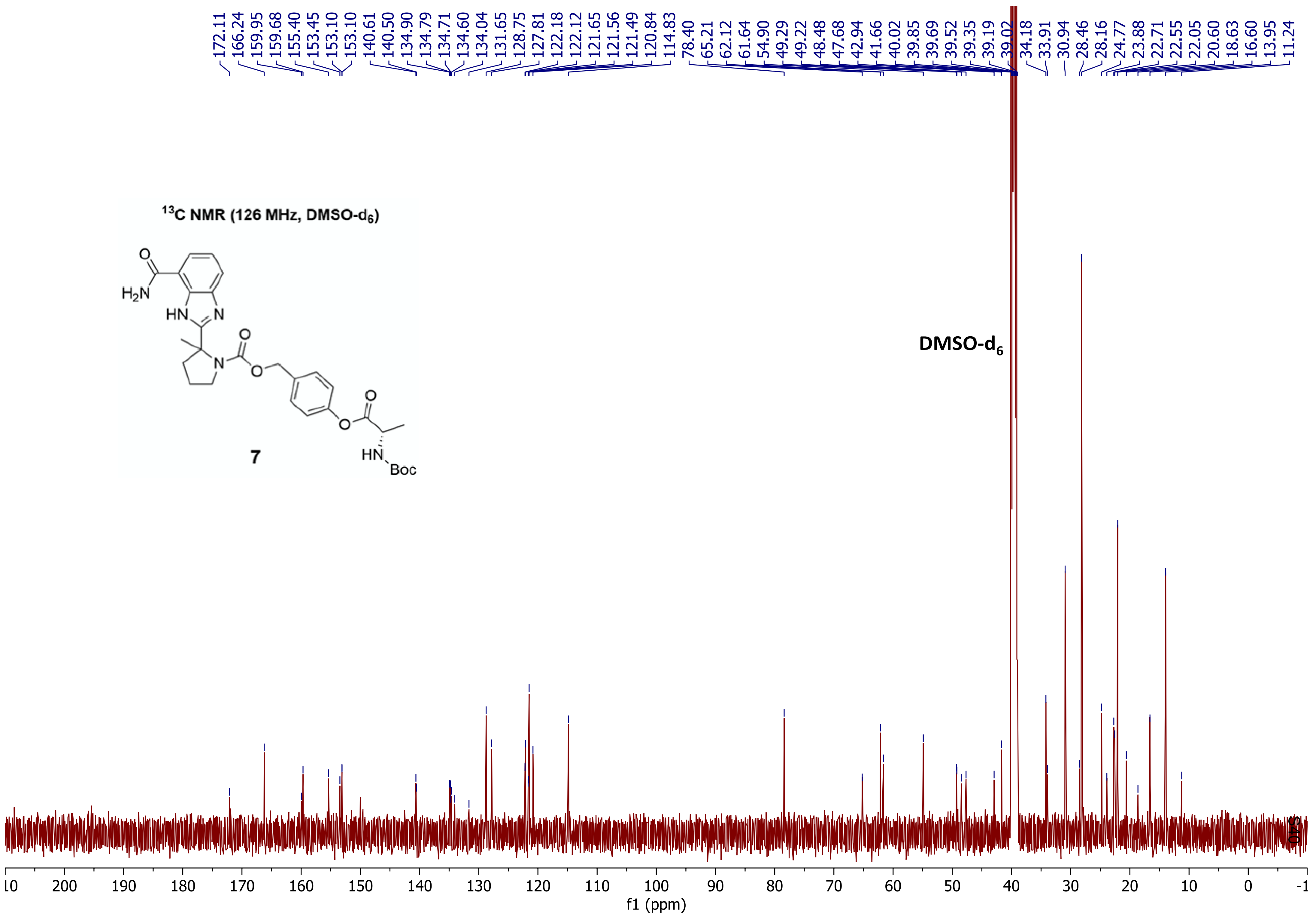

S40
13C NMR (126 MHz, DMSO-d6)
H2N
O
HN
N
N
O
O
O
O
HN
Boc
7
DMSO-d6
172.11
166.24
159.95
159.68
155.40
153.45
153.10
153.10
140.61
140.50
134.90
134.79
134.71
134.60
134.04
131.65
128.75
127.81
122.18
122.12
121.65
121.56
121.49
120.84
114.83
78.40
65.21
62.12
61.64
54.90
49.29
49.22
48.48
47.68
42.94
41.66
40.02
39.85
39.69
39.52
39.35
39.19
39.02
34.18
33.91
30.94
28.46
28.16
24.77
23.88
22.71
22.55
22.05
20.60
18.63
16.60
13.95
11.24
f1 (ppm)

^1H NMR (400 MHz, DMSO-d$_6$, 60°C)

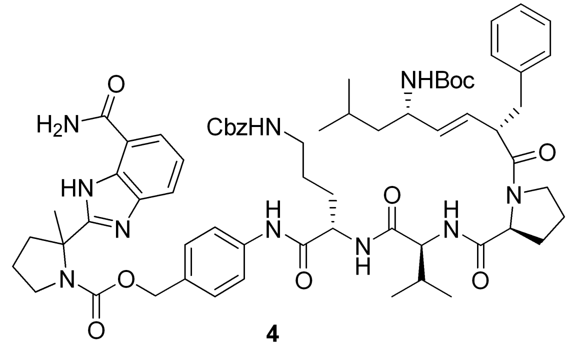

4

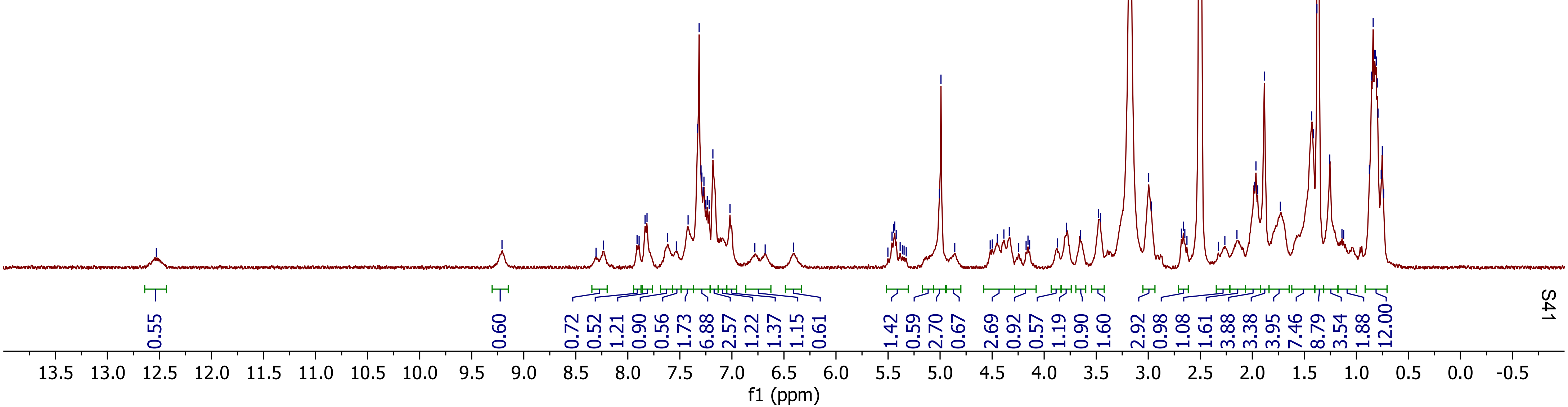

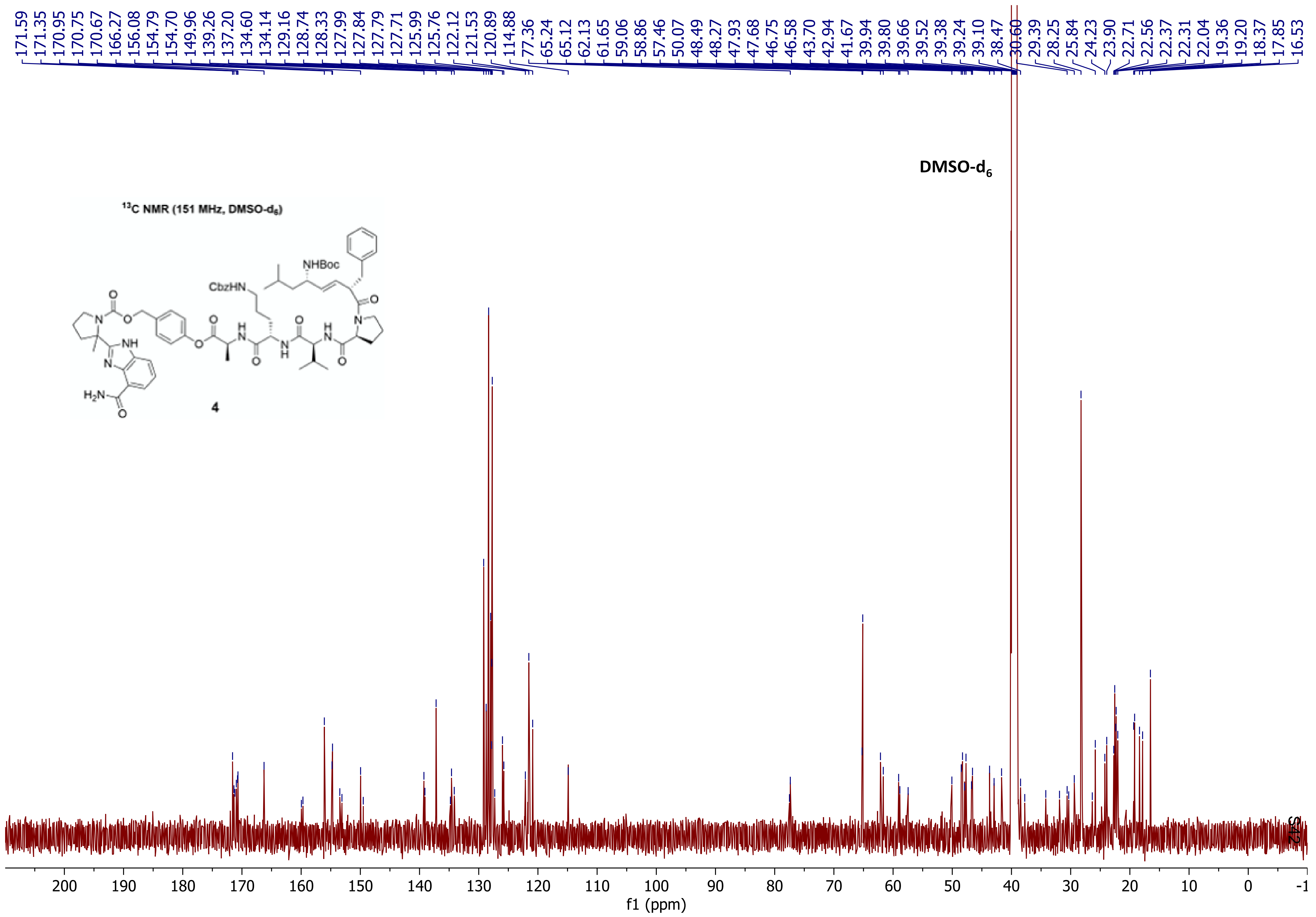
171.59
171.35
170.95
170.75
170.67
166.27
156.08
154.79
154.70
149.96
139.26
137.20
134.60
134.14
129.16
128.74
128.33
127.99
127.84
127.79
127.71
125.99
125.76
122.12
121.53
120.89
114.88
77.36
65.24
65.12
62.13
61.65
59.06
58.86
57.46
50.07
48.49
48.27
47.93
47.68
46.75
46.58
43.70
42.94
41.67
39.94
39.80
39.66
39.52
39.38
39.24
39.10
38.47
30.60
29.39
28.25
25.84
24.23
23.90
22.71
22.56
22.37
22.31
22.04
19.36
19.20
18.37
17.85
16.53
DMSO-d6
13C NMR (151 MHz, DMSO-d6)
NHBoc
CbzHN
H2N
NH
4
f1 (ppm)

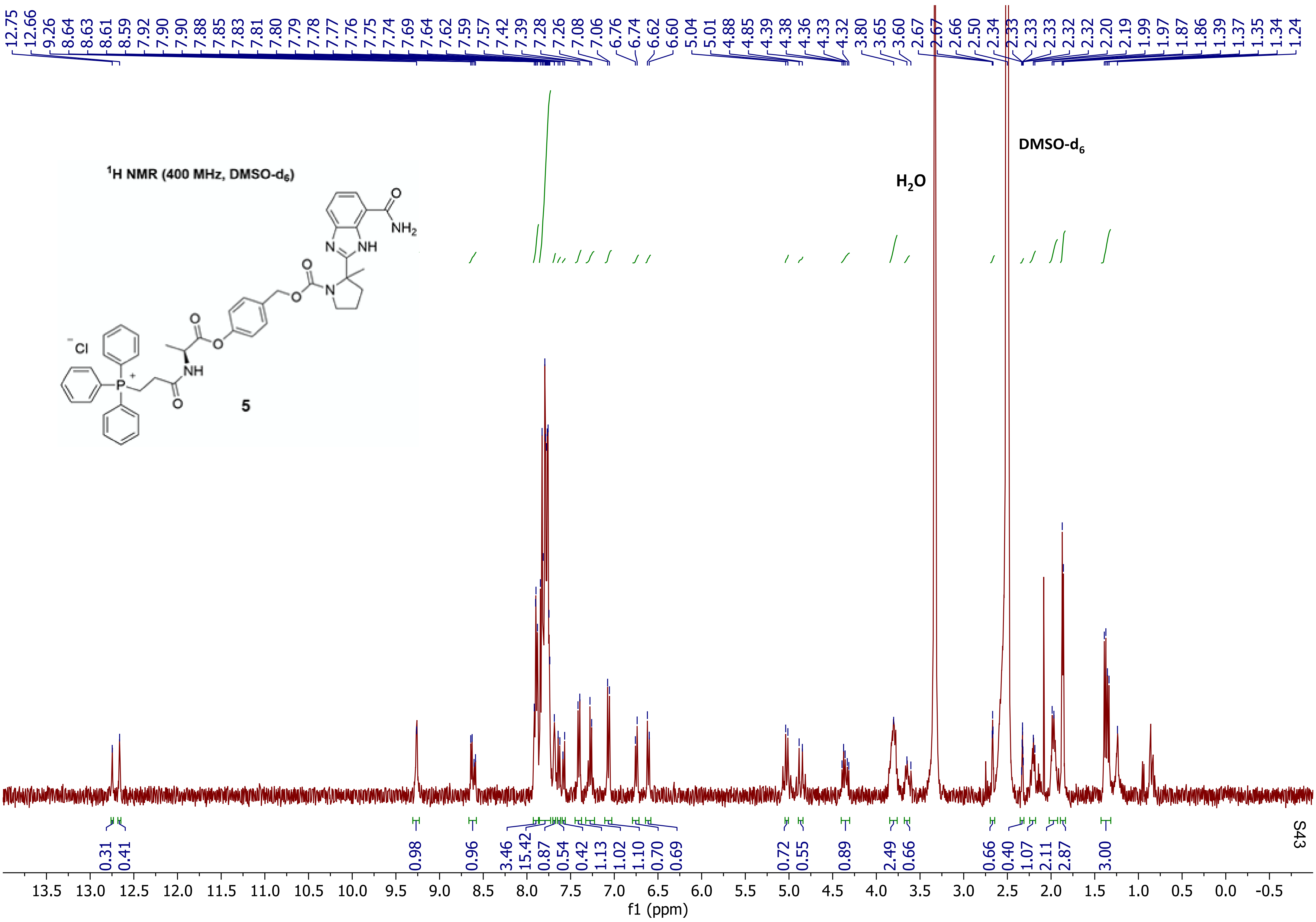

12.75 12.66 9.26 8.64 8.63 8.61 8.59 7.92 7.90 7.90 7.88 7.85 7.83 7.81 7.80 7.79 7.78 7.77 7.76 7.75 7.74 7.69 7.64 7.62 7.59 7.57 7.42 7.39 7.28 7.26 7.08 7.06 6.76 6.74 6.62 6.60 5.04 5.01 4.88 4.85 4.39 4.38 4.36 4.33 4.32 3.80 3.65 3.60 2.67 2.67 2.66 2.50 2.34 2.33 2.33 2.33 2.32 2.32 2.20 2.19 1.99 1.97 1.87 1.86 1.39 1.37 1.35 1.34 1.24
1H NMR (400 MHz, DMSO-d6)
5
H2O
DMSO-d6
0.31 0.41 0.98 0.96 3.46 15.42 0.87 0.54 0.42 1.13 1.02 1.10 0.70 0.69 0.72 0.55 0.89 2.49 0.66 0.66 0.40 1.07 2.11 2.87 3.00
13.5 13.0 12.5 12.0 11.5 11.0 10.5 10.0 9.5 9.0 8.5 8.0 7.5 7.0 6.5 6.0 5.5 5.0 4.5 4.0 3.5 3.0 2.5 2.0 1.5 1.0 0.5 0.0 -0.5
f1 (ppm)

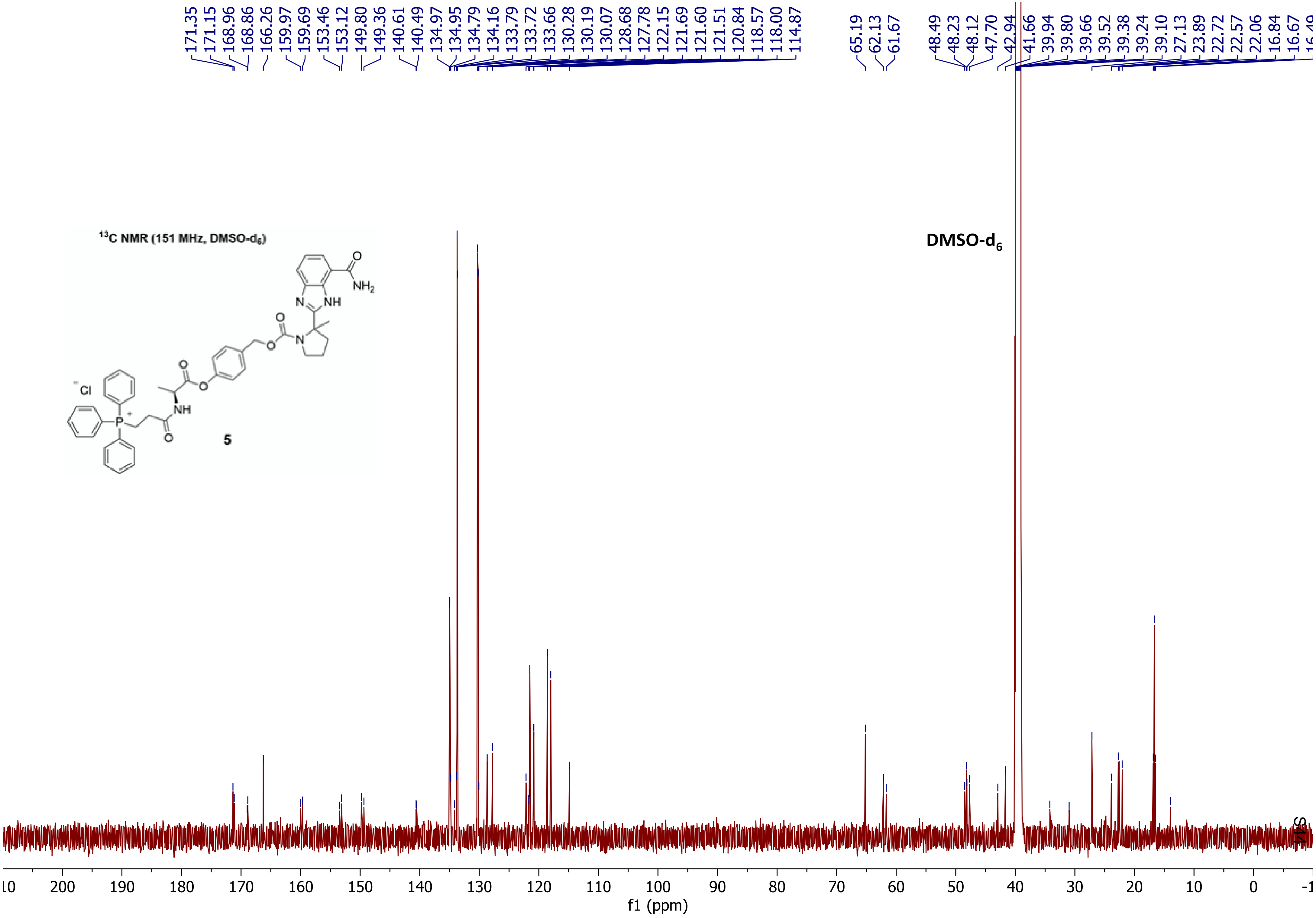
171.35
171.15
168.96
168.86
166.26
159.97
159.69
153.46
153.12
149.80
149.36
140.61
140.49
134.97
134.95
134.79
134.16
133.79
133.72
133.66
130.28
130.19
130.07
128.68
127.78
122.15
121.69
121.60
121.51
120.84
118.57
118.00
114.87
65.19
62.13
61.67
48.49
48.23
48.12
47.70
42.94
41.66
39.94
39.80
39.66
39.52
39.38
39.24
39.10
27.13
23.89
22.72
22.57
22.06
16.84
16.67
^{13}C NMR (151 MHz, DMSO-d_6)
DMSO-d_6
5
NH2
NH
N
O
Cl
P
f1 (ppm)

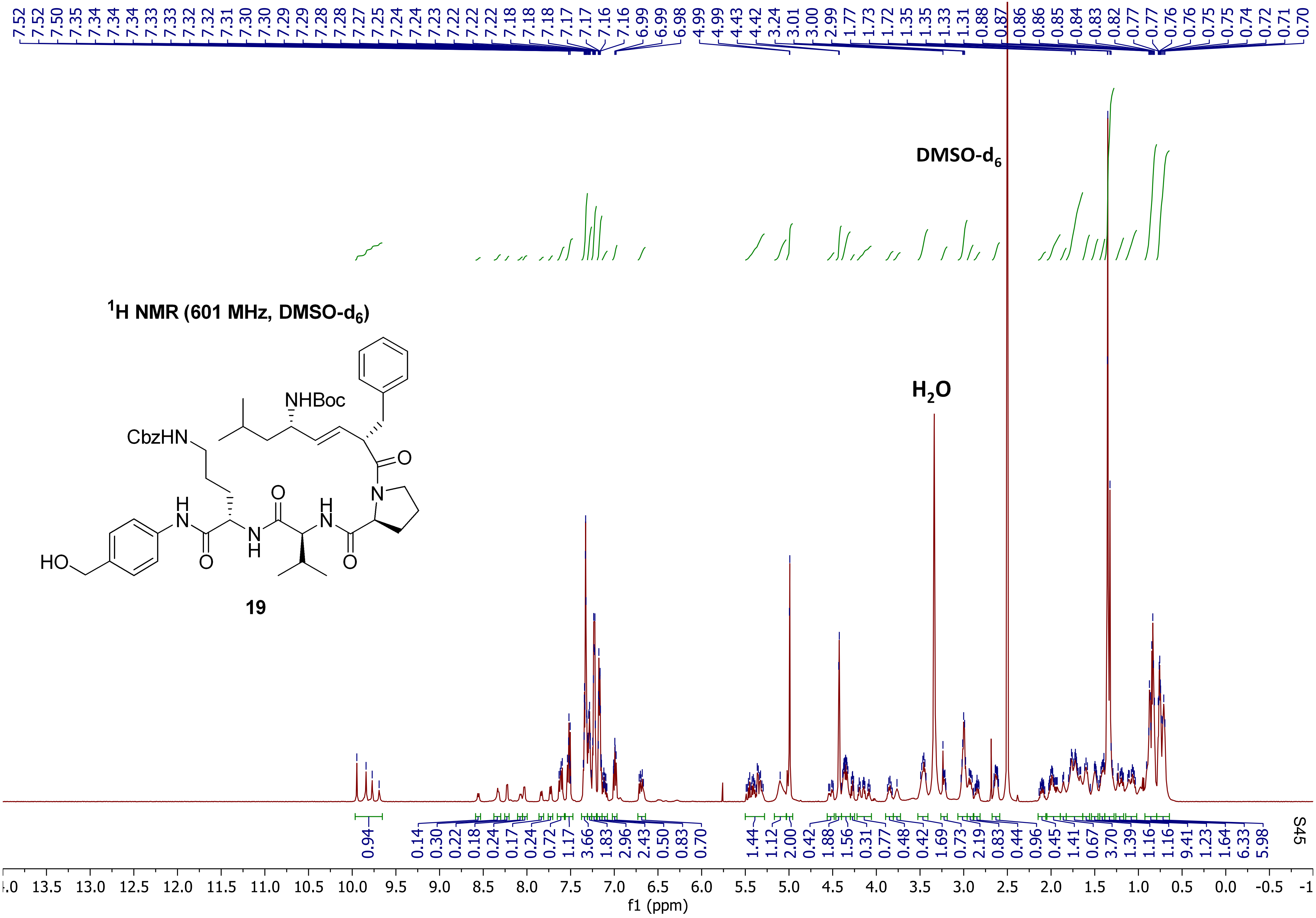
7.52 7.52 7.50 7.35 7.34 7.34 7.33 7.33 7.32 7.32 7.31 7.30 7.30 7.29 7.29 7.28 7.28 7.27 7.25 7.24 7.24 7.23 7.22 7.22 7.22 7.18 7.18 7.18 7.17 7.17 7.16 7.16 6.99 6.99 6.98 4.99 4.99 4.43 4.42 3.24 3.01 3.00 2.99 1.77 1.73 1.72 1.35 1.35 1.33 1.31 0.88 0.87 0.86 0.86 0.85 0.84 0.83 0.82 0.77 0.77 0.76 0.76 0.75 0.75 0.74 0.72 0.71 0.70
DMSO-d6
H2O
1H NMR (601 MHz, DMSO-d6)
CbzHN
NHBoc
HO
19
0.94 0.14 0.30 0.22 0.18 0.24 0.17 0.24 0.72 1.17 3.66 1.83 2.96 2.43 0.50 0.83 0.70 1.44 1.12 2.00 0.42 1.88 1.56 0.31 0.77 0.48 0.42 1.69 0.73 2.19 0.83 0.44 0.96 0.45 1.41 0.67 3.70 1.39 1.16 1.16 9.41 1.23 1.64 6.33 5.98
f1 (ppm)

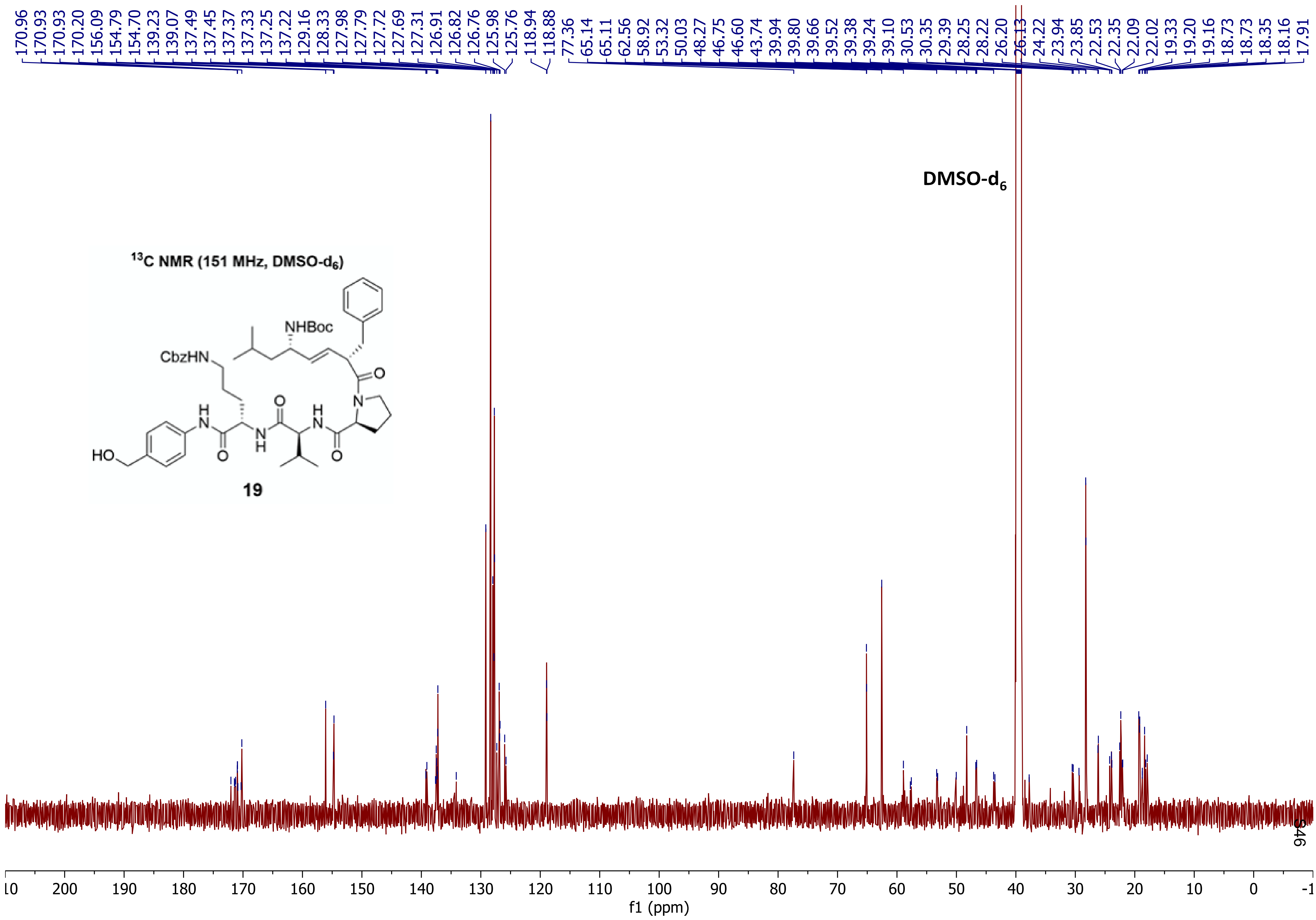
170.96
170.93
170.93
170.20
156.09
154.79
154.70
139.23
139.07
137.49
137.45
137.37
137.33
137.25
137.22
129.16
128.33
127.98
127.79
127.72
127.69
127.31
126.91
126.82
126.76
125.98
125.76
118.94
118.88
77.36
65.14
65.11
62.56
58.92
53.32
50.03
48.27
46.75
46.60
43.74
39.94
39.80
39.66
39.52
39.38
39.24
39.10
30.53
30.35
29.39
28.25
28.22
26.20
26.13
24.22
23.94
23.85
22.53
22.35
22.09
22.02
19.33
19.20
19.16
18.73
18.73
18.35
18.16
17.91
DMSO-d6
13C NMR (151 MHz, DMSO-d6)
NHBoc
CbzHN
HO
19
f1 (ppm)

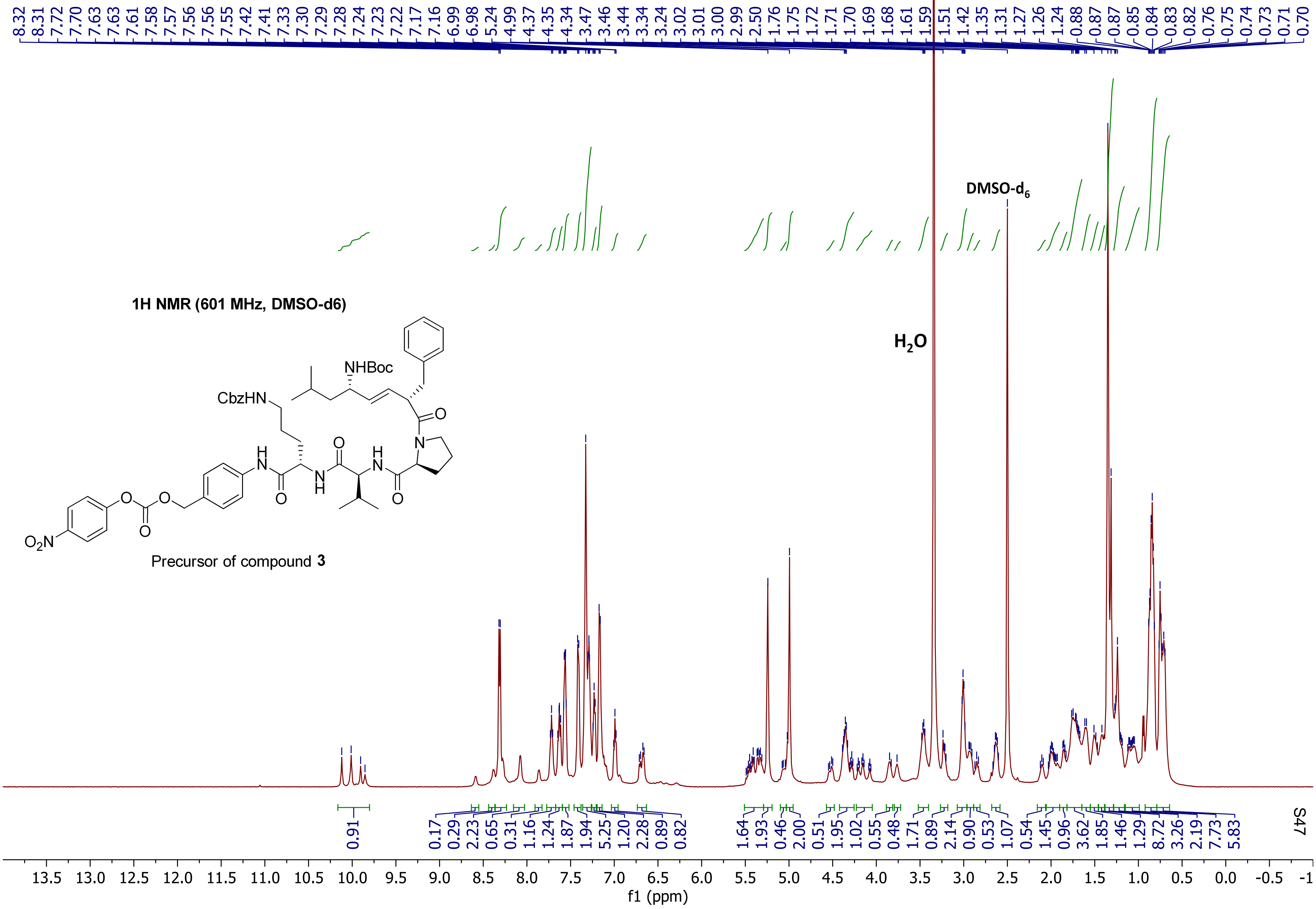
8.32 8.31 7.72 7.70 7.63 7.63 7.61 7.58 7.57 7.56 7.56 7.55 7.42 7.41 7.33 7.30 7.29 7.28 7.24 7.23 7.22 7.17 7.16 6.99 6.98 5.24 4.99 4.37 4.35 4.34 3.47 3.46 3.44 3.34 3.24 3.02 3.01 3.00 2.99 2.50 1.76 1.75 1.72 1.71 1.70 1.69 1.68 1.61 1.59 1.51 1.42 1.35 1.31 1.27 1.26 1.24 0.88 0.87 0.87 0.85 0.84 0.83 0.82 0.76 0.75 0.74 0.73 0.71 0.70
1H NMR (601 MHz, DMSO-d6)
NHBoc
CbzHN
O₂N
Precursor of compound 3
H₂O
DMSO-d₆
0.91
0.17 0.29 2.23 0.65 0.31 1.16 1.24 1.87 1.94 5.25 1.20 2.28 0.89 0.82
1.64 1.93 0.46 2.00 0.51 1.95 1.02 0.55 0.48 1.71 0.89 2.14 0.90 0.53 1.07 0.54 1.45 0.96 3.62 1.85 1.46 1.29 8.72 3.26 2.19 7.73 5.83
13.5 13.0 12.5 12.0 11.5 11.0 10.5 10.0 9.5 9.0 8.5 8.0 7.5 7.0 6.5 6.0 5.5 5.0 4.5 4.0 3.5 3.0 2.5 2.0 1.5 1.0 0.5 0.0 -0.5 -1
f1 (ppm)

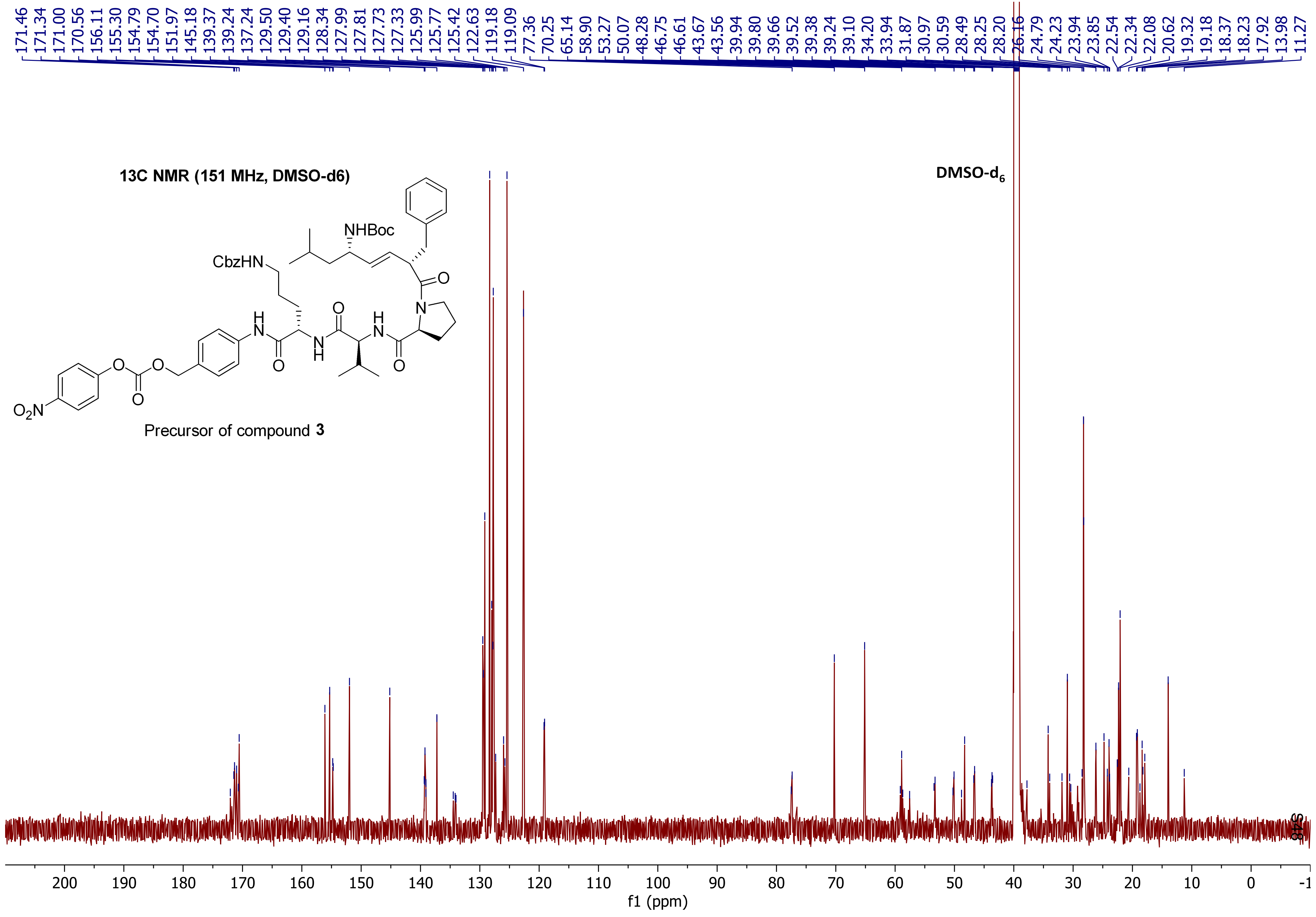
171.46
171.34
171.00
170.56
156.11
155.30
154.79
154.70
151.97
145.18
139.37
139.24
137.24
129.50
129.40
129.16
128.34
127.99
127.81
127.73
127.33
125.99
125.77
125.42
122.63
119.18
119.09
77.36
70.25
65.14
58.90
53.27
50.07
48.28
46.75
46.61
43.67
43.56
39.94
39.80
39.66
39.52
39.38
39.24
39.10
34.20
33.94
31.87
30.97
30.59
28.49
28.25
28.20
26.16
24.79
24.23
23.94
23.85
22.54
22.34
22.08
20.62
19.32
19.18
18.37
18.23
17.92
13.98
11.27
13C NMR (151 MHz, DMSO-d6)
DMSO-d_6
NHBoc
CbzHN
O_2N
Precursor of compound 3
S48
200
190
180
170
160
150
140
130
120
110
100
90
80
70
60
50
40
30
20
10
0
-1
f1 (ppm)

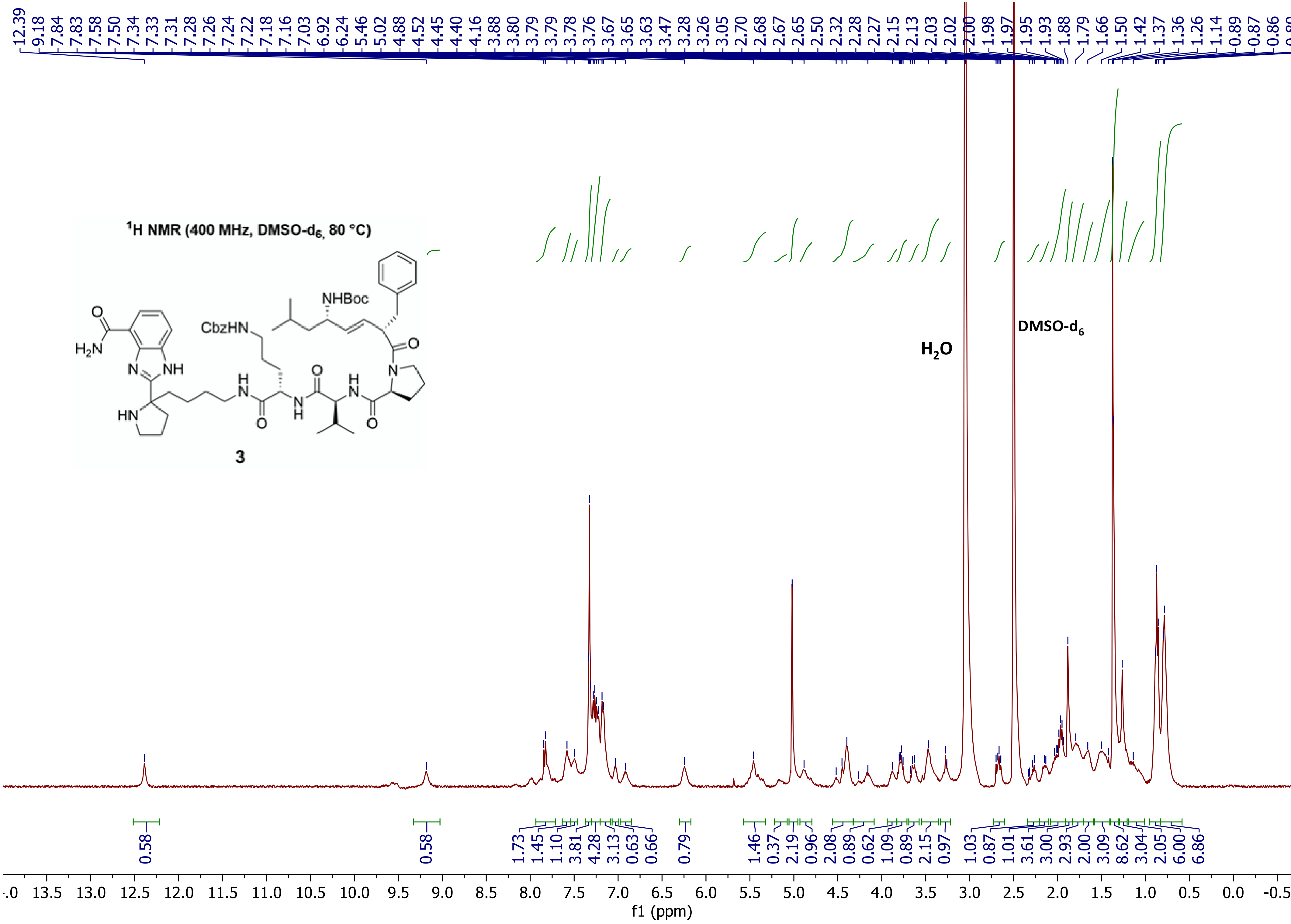
¹H NMR (400 MHz, DMSO-d6, 80 °C)
3
H2O
DMSO-d6
f1 (ppm)

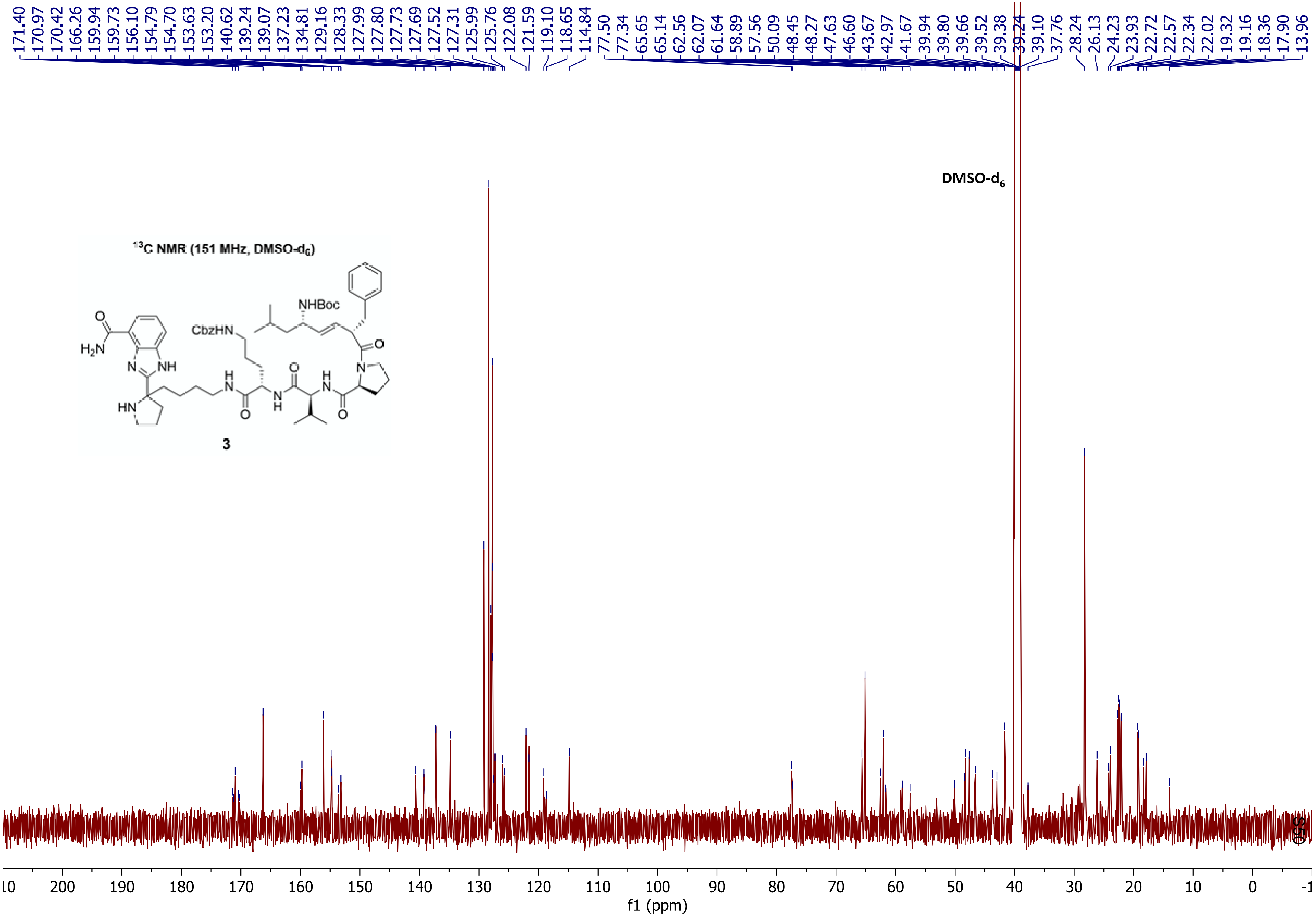
171.40
170.97
170.42
166.26
159.94
159.73
156.10
154.79
154.70
153.63
153.20
140.62
139.24
139.07
137.23
134.81
129.16
128.33
127.99
127.80
127.73
127.69
127.52
127.31
125.99
125.76
122.08
121.59
119.10
118.65
114.84
77.50
77.34
65.65
65.14
62.56
62.07
61.64
58.89
57.56
50.09
48.45
48.27
47.63
46.60
43.67
42.97
41.67
39.94
39.80
39.66
39.52
39.38
39.24
39.10
37.76
28.24
26.13
24.23
23.93
22.72
22.57
22.34
22.02
19.32
19.16
18.36
17.90
13.96
DMSO-d6
13C NMR (151 MHz, DMSO-d6)
CbzHN
NHBoc
H2N
NH
HN
3
f1 (ppm)

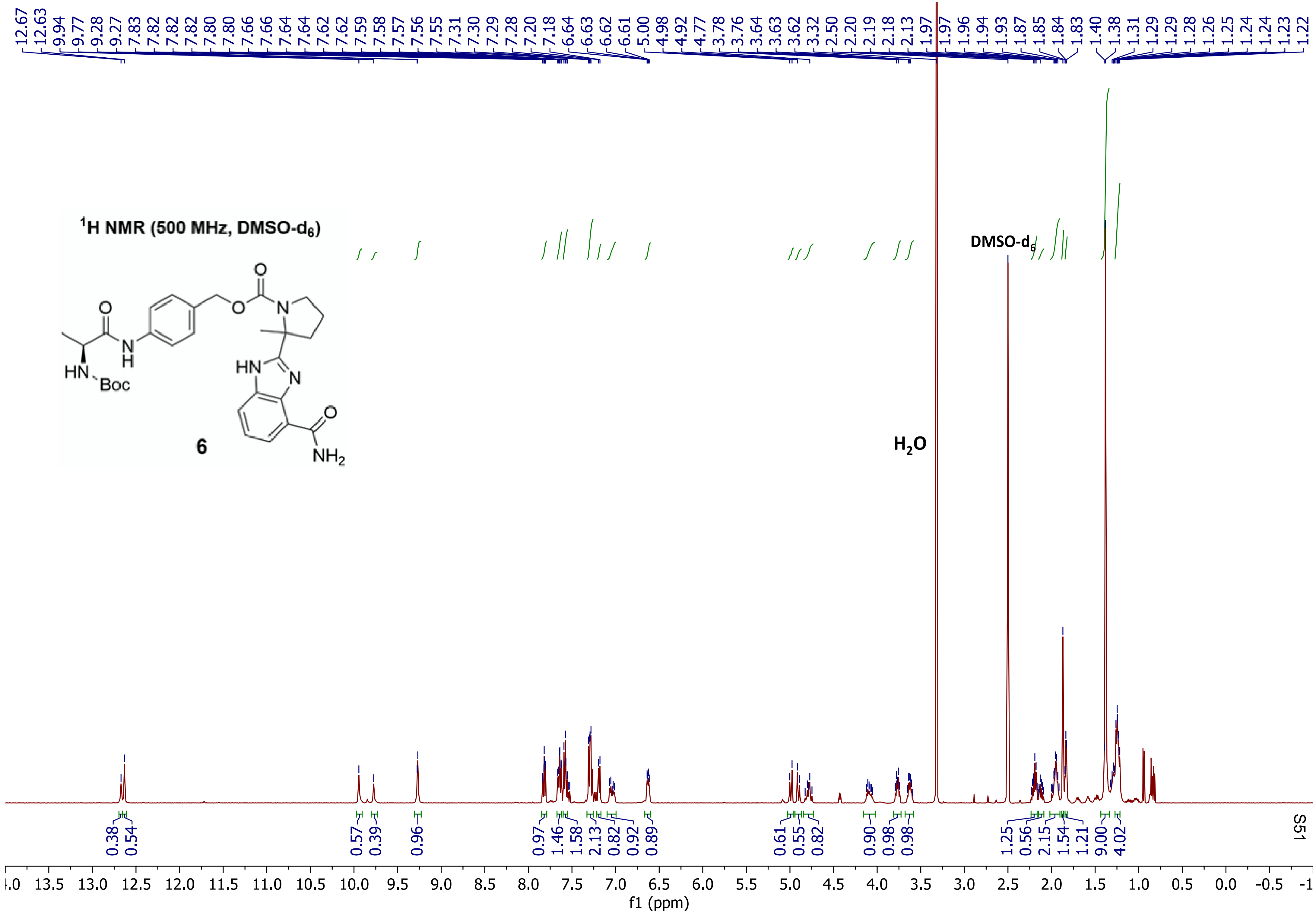

12.67 12.63 9.94 9.77 9.28 9.27 7.83 7.82 7.82 7.82 7.80 7.80 7.66 7.66 7.64 7.64 7.62 7.62 7.59 7.58 7.57 7.56 7.55 7.31 7.30 7.29 7.28 7.20 7.18 6.64 6.63 6.62 6.61 5.00 4.98 4.92 4.77 3.78 3.76 3.64 3.63 3.62 3.32 2.50 2.20 2.19 2.18 2.13 1.97 1.97 1.96 1.94 1.93 1.87 1.85 1.84 1.83 1.40 1.38 1.31 1.29 1.29 1.28 1.26 1.25 1.24 1.24 1.23 1.22
¹H NMR (500 MHz, DMSO-d6)
HN
Boc
N
H
O
O
N
HN
N
O
NH2
6
DMSO-d6
H2O
0.38 0.54 0.57 0.39 0.96 0.97 1.46 1.58 2.13 0.82 0.92 0.89 0.61 0.55 0.82 0.90 0.98 0.98 1.25 0.56 2.15 1.54 1.21 9.00 4.02
4.0 13.5 13.0 12.5 12.0 11.5 11.0 10.5 10.0 9.5 9.0 8.5 8.0 7.5 7.0 6.5 6.0 5.5 5.0 4.5 4.0 3.5 3.0 2.5 2.0 1.5 1.0 0.5 0.0 -0.5 -1
f1 (ppm)

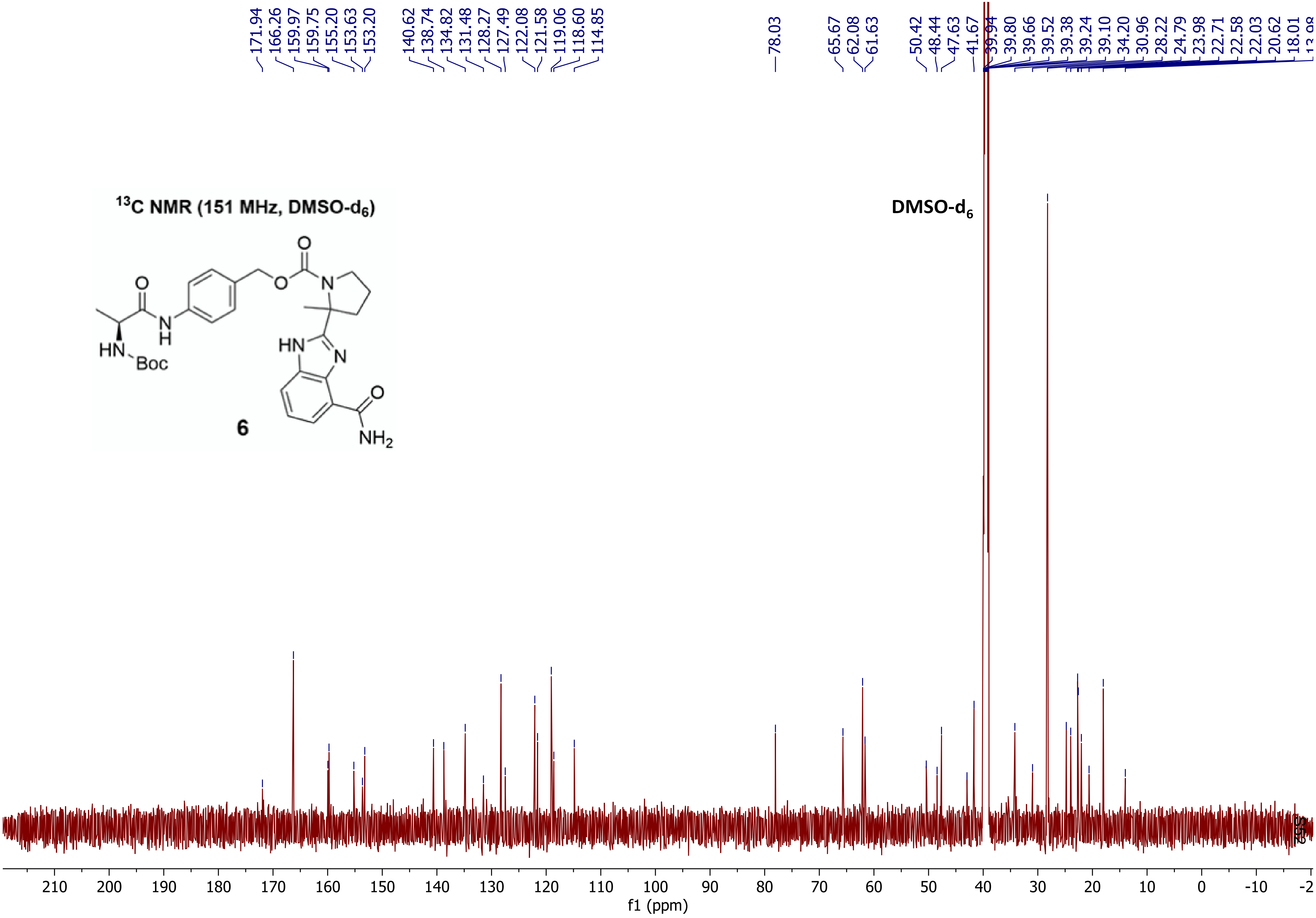
171.94
166.26
159.97
159.75
155.20
153.63
153.20
140.62
138.74
134.82
131.48
128.27
127.49
122.08
121.58
119.06
118.60
114.85
78.03
65.67
62.08
61.63
50.42
48.44
47.63
41.67
39.94
39.80
39.66
39.52
39.38
39.24
39.10
34.20
30.96
28.22
24.79
23.98
22.71
22.58
22.03
20.62
18.01
[illegible]
^{13}C NMR (151 MHz, DMSO-d_6)
O
N
H
HN
Boc
O
O
N
HN
N
O
NH$_2$
6
DMSO-d_6
210
200
190
180
170
160
150
140
130
120
110
100
90
80
70
60
50
40
30
20
10
0
-10
-2
f1 (ppm)
